# Solving olympiad geometry without human demonstrations

**DOI:** 10.1038/s41586-023-06747-5

**Published:** 2024-01-17

**Authors:** Trieu H. Trinh, Yuhuai Wu, Quoc V. Le, He He, Thang Luong

**Affiliations:** 1 Google Deepmind, Mountain View, CA USA; 2 Computer Science Department, New York University, New York, NY USA

**Keywords:** Computer science, Computational science

## Abstract

Proving mathematical theorems at the olympiad level represents a notable milestone in human-level automated reasoning^1–4^, owing to their reputed difficulty among the world’s best talents in pre-university mathematics. Current machine-learning approaches, however, are not applicable to most mathematical domains owing to the high cost of translating human proofs into machine-verifiable format. The problem is even worse for geometry because of its unique translation challenges^1,5^, resulting in severe scarcity of training data. We propose AlphaGeometry, a theorem prover for Euclidean plane geometry that sidesteps the need for human demonstrations by synthesizing millions of theorems and proofs across different levels of complexity. AlphaGeometry is a neuro-symbolic system that uses a neural language model, trained from scratch on our large-scale synthetic data, to guide a symbolic deduction engine through infinite branching points in challenging problems. On a test set of 30 latest olympiad-level problems, AlphaGeometry solves 25, outperforming the previous best method that only solves ten problems and approaching the performance of an average International Mathematical Olympiad (IMO) gold medallist. Notably, AlphaGeometry produces human-readable proofs, solves all geometry problems in the IMO 2000 and 2015 under human expert evaluation and discovers a generalized version of a translated IMO theorem in 2004.

## Abstract (web summary)

A new neuro-symbolic theorem prover for Euclidean plane geometry trained from scratch on millions of synthesized theorems and proofs outperforms the previous best method and reaches the performance of an olympiad gold medallist.

## Main

Proving theorems showcases the mastery of logical reasoning and the ability to search through an infinitely large space of actions towards a target, signifying a remarkable problem-solving skill. Since the 1950s (refs. ^6,7^), the pursuit of better theorem-proving capabilities has been a constant focus of artificial intelligence (AI) research^8^. Mathematical olympiads are the most reputed theorem-proving competitions in the world, with a similarly long history dating back to 1959, playing an instrumental role in identifying exceptional talents in problem solving. Matching top human performances at the olympiad level has become a notable milestone of AI research^2–4^.

Theorem proving is difficult for learning-based methods because training data of human proofs translated into machine-verifiable languages are scarce in most mathematical domains. Geometry stands out among other olympiad domains because it has very few proof examples in general-purpose mathematical languages such as Lean^9^ owing to translation difficulties unique to geometry^1,5^. Geometry-specific languages, on the other hand, are narrowly defined and thus unable to express many human proofs that use tools beyond the scope of geometry, such as complex numbers (Extended Data Figs. 3 and 4). Overall, this creates a data bottleneck, causing geometry to lag behind in recent progress that uses human demonstrations^2–4^. Current approaches to geometry, therefore, still primarily rely on symbolic methods and human-designed, hard-coded search heuristics^10–14^.

We present an alternative method for theorem proving using synthetic data, thus sidestepping the need for translating human-provided proof examples. We focus on Euclidean plane geometry and exclude topics such as geometric inequalities and combinatorial geometry. By using existing symbolic engines on a diverse set of random theorem premises, we extracted 100 million synthetic theorems and their proofs, many with more than 200 proof steps, four times longer than the average proof length of olympiad theorems. We further define and use the concept of dependency difference in synthetic proof generation, allowing our method to produce nearly 10 million synthetic proof steps that construct auxiliary points, reaching beyond the scope of pure symbolic deduction. Auxiliary construction is geometry’s instance of exogenous term generation, representing the infinite branching factor of theorem proving, and widely recognized in other mathematical domains as the key challenge to proving many hard theorems^1,2^. Our work therefore demonstrates a successful case of generating synthetic data and learning to solve this key challenge. With this solution, we present a general guiding framework and discuss its applicability to other domains in Methods section ‘AlphaGeometry framework and applicability to other domains’.

**Fig. 1:**
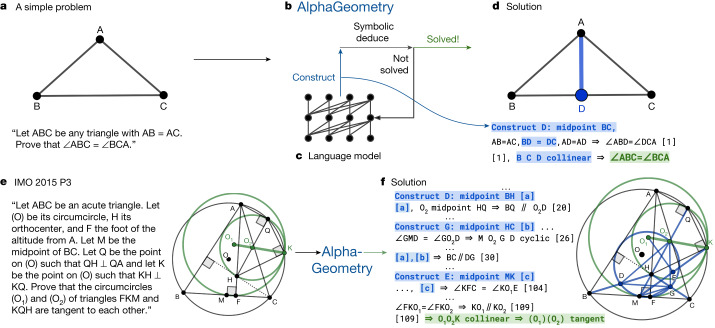
Overview of our neuro-symbolic AlphaGeometry and how it solves both a simple problem and the IMO 2015 Problem 3. The top row shows how AlphaGeometry solves a simple problem. **a**, The simple example and its diagram. **b**, AlphaGeometry initiates the proof search by running the symbolic deduction engine. The engine exhaustively deduces new statements from the theorem premises until the theorem is proven or new statements are exhausted. **c**, Because the symbolic engine fails to find a proof, the language model constructs one auxiliary point, growing the proof state before the symbolic engine retries. The loop continues until a solution is found. **d**, For the simple example, the loop terminates after the first auxiliary construction “D as the midpoint of BC”. The proof consists of two other steps, both of which make use of the midpoint properties: “BD = DC” and “B, D, C are collinear”, highlighted in blue. The bottom row shows how AlphaGeometry solves the IMO 2015 Problem 3 (IMO 2015 P3). **e**, The IMO 2015 P3 problem statement and diagram. **f**, The solution of IMO 2015 P3 has three auxiliary points. In both solutions, we arrange language model outputs (blue) interleaved with symbolic engine outputs to reflect their execution order. Note that the proof for IMO 2015 P3 in **f** is greatly shortened and edited for illustration purposes. Its full version is in the Supplementary Information.

**Fig. 2:**
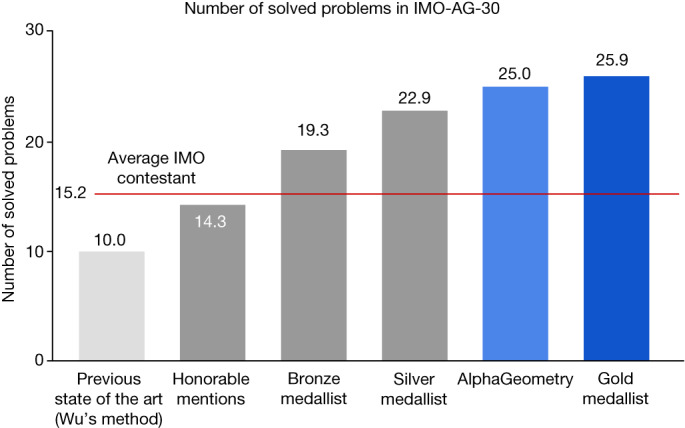
AlphaGeometry advances the current state of geometry theorem prover from below human level to near gold-medallist level. The test benchmark includes official IMO problems from 2000 to the present that can be represented in the geometry environment used in our work. Human performance is estimated by rescaling their IMO contest scores between 0 and 7 to between 0 and 1, to match the binary outcome of failure/success of the machines. For example, a contestant’s score of 4 out of 7 will be scaled to 0.57 problems in this comparison. On the other hand, the score for AlphaGeometry and other machine solvers on any problem is either 0 (not solved) or 1 (solved). Note that this is only an approximate comparison with humans on classical geometry, who operate on natural-language statements rather than narrow, domain-specific translations. Further, the general IMO contest also includes other types of problem, such as geometric inequality or combinatorial geometry, and other domains of mathematics, such as algebra, number theory and combinatorics. Source Data

We pretrain a language model on all generated synthetic data and fine-tune it to focus on auxiliary construction during proof search, delegating all deduction proof steps to specialized symbolic engines. This follows standard settings in the literature, in which language models such as GPT-f (ref. ^15^), after being trained on human proof examples, can generate exogenous proof terms as inputs to fast and accurate symbolic engines such as nlinarith or ring^2,3,16^, using the best of both worlds. Our geometry theorem prover AlphaGeometry, illustrated in Fig. 1, produces human-readable proofs, substantially outperforms the previous state-of-the-art geometry-theorem-proving computer program and approaches the performance of an average IMO gold medallist on a test set of 30 classical geometry problems translated from the IMO as shown in Fig. 2.

## Synthetic theorems and proofs generation

**Fig. 3:**
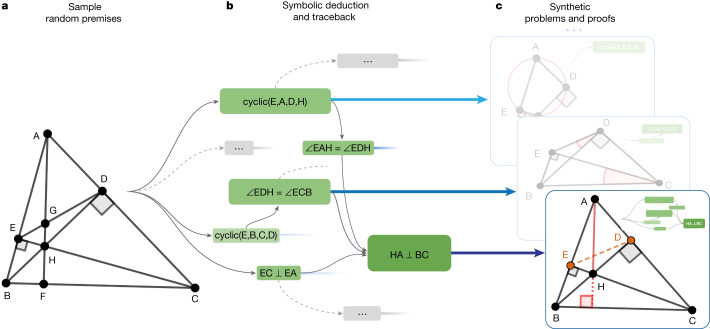
AlphaGeometry synthetic-data-generation process. **a**, We first sample a large set of random theorem premises. **b**, We use the symbolic deduction engine to obtain a deduction closure. This returns a directed acyclic graph of statements. For each node in the graph, we perform traceback to find its minimal set of necessary premise and dependency deductions. For example, for the rightmost node ‘HA ⊥ BC’, traceback returns the green subgraph. **c**, The minimal premise and the corresponding subgraph constitute a synthetic problem and its solution. In the bottom example, points E and D took part in the proof despite being irrelevant to the construction of HA and BC; therefore, they are learned by the language model as auxiliary constructions.

Our method for generating synthetic data is shown in Fig. 3. We first sample a random set of theorem premises, serving as the input to the symbolic deduction engine to generate its derivations. A full list of actions used for this sampling can be found in Extended Data Table 1. In our work, we sampled nearly 1 billion of such premises in a highly parallelized setting, described in Methods. Note that we do not make use of any existing theorem premises from human-designed problem sets and sampled the eligible constructions uniformly randomly.

Next we use a symbolic deduction engine on the sampled premises. The engine quickly deduces new true statements by following forward inference rules as shown in Fig. 3b. This returns a directed acyclic graph of all reachable conclusions. Each node in the directed acyclic graph is a reachable conclusion, with edges connecting to its parent nodes thanks to the traceback algorithm described in Methods. This allows a traceback process to run recursively starting from any node *N*, at the end returning its dependency subgraph *G*(*N*), with its root being *N* and its leaves being a subset of the sampled premises. Denoting this subset as *P*, we obtained a synthetic training example (premises, conclusion, proof) = (*P*, *N*, *G*(*N*)).

In geometry, the symbolic deduction engine is deductive database (refs. ^10,17^), with the ability to efficiently deduce new statements from the premises by means of geometric rules. DD follows deduction rules in the form of definite Horn clauses, that is, *Q*(*x*) ← *P*_1_(*x*),…, *P*_*k*_(*x*), in which *x* are points objects, whereas *P*_1_,…, *P*_*k*_ and *Q* are predicates such as ‘equal segments’ or ‘collinear’. A full list of deduction rules can be found in ref. ^10^. To widen the scope of the generated synthetic theorems and proofs, we also introduce another component to the symbolic engine that can deduce new statements through algebraic rules (AR), as described in Methods. AR is necessary to perform angle, ratio and distance chasing, as often required in many olympiad-level proofs. We included concrete examples of AR in Extended Data Table 2. The combination DD + AR, which includes both their forward deduction and traceback algorithms, is a new contribution in our work and represents a new state of the art in symbolic reasoning in geometry.

## Generating proofs beyond symbolic deduction

So far, the generated proofs consist purely of deduction steps that are already reachable by the highly efficient symbolic deduction engine DD + AR. To solve olympiad-level problems, however, the key missing piece is generating new proof terms. In the above algorithm, it can be seen that such terms form the subset of *P* that *N* is independent of. In other words, these terms are the dependency difference between the conclusion statement and the conclusion objects. We move this difference from *P* to the proof so that a generative model that learns to generate the proof can learn to construct them, as illustrated in Fig. 3c. Such proof steps perform auxiliary constructions that symbolic deduction engines are not designed to do. In the general theorem-proving context, auxiliary construction is an instance of exogenous term generation, a notable challenge to all proof-search algorithms because it introduces infinite branching points to the search tree. In geometry theorem proving, auxiliary constructions are the longest-standing subject of study since inception of the field in 1959 (refs. ^6,7^). Previous methods to generate them are based on hand-crafted templates and domain-specific heuristics^8–12^, and are, therefore, limited by a subset of human experiences expressible in hard-coded rules. Any neural solver trained on our synthetic data, on the other hand, learns to perform auxiliary constructions from scratch without human demonstrations.

## Training a language model on synthetic data

The transformer^18^ language model is a powerful deep neural network that learns to generate text sequences through next-token prediction, powering substantial advances in generative AI technology. We serialize (*P*, *N*, *G*(*N*)) into a text string with the structure ‘<premises><conclusion><proof>’. By training on such sequences of symbols, a language model effectively learns to generate the proof, conditioning on theorem premises and conclusion.

## Combining language modelling and symbolic engines

On a high level, proof search is a loop in which the language model and the symbolic deduction engine take turns to run, as shown in Fig. 1b,c. Proof search terminates whenever the theorem conclusion is found or when the loop reaches a maximum number of iterations. The language model is seeded with the problem statement string and generates one extra sentence at each turn, conditioning on the problem statement and past constructions, describing one new auxiliary construction such as “construct point X so that ABCX is a parallelogram”. Each time the language model generates one such construction, the symbolic engine is provided with new inputs to work with and, therefore, its deduction closure expands, potentially reaching the conclusion. We use beam search to explore the top *k* constructions generated by the language model and describe the parallelization of this proof-search algorithm in Methods.

## Empirical evaluation

### An olympiad-level benchmark for geometry

Existing benchmarks of olympiad mathematics do not cover geometry because of a focus on formal mathematics in general-purpose languages^1,9^, whose formulation poses great challenges to representing geometry. Solving these challenges requires deep expertise and large research investment that are outside the scope of our work, which focuses on a methodology for theorem proving. For this reason, we adapted geometry problems from the IMO competitions since 2000 to a narrower, specialized environment for classical geometry used in interactive graphical proof assistants^13,17,19^, as discussed in Methods. Among all non-combinatorial geometry-related problems, 75% can be represented, resulting in a test set of 30 classical geometry problems. Geometric inequality and combinatorial geometry, for example, cannot be translated, as their formulation is markedly different to classical geometry. We include the full list of statements and translations for all 30 problems in the Supplementary Information. The final test set is named IMO-AG-30, highlighting its source, method of translation and its current size.

## Geometry theorem prover baselines

Geometry theorem provers in the literature fall into two categories. The first category is computer algebra methods, which treats geometry statements as polynomial equations of its point coordinates. Proving is accomplished with specialized transformations of large polynomials. Gröbner bases^20^ and Wu’s method^21^ are representative approaches in this category, with theoretical guarantees to successfully decide the truth value of all geometry theorems in IMO-AG-30, albeit without a human-readable proof. Because these methods often have large time and memory complexity, especially when processing IMO-sized problems, we report their result by assigning success to any problem that can be decided within 48 h using one of their existing implementations^17^.

AlphaGeometry belongs to the second category of solvers, often described as search/axiomatic or sometime*s* ‘synthetic’ methods. These methods treat the problem of theorem proving as a step-by-step search problem using a set of geometry axioms. Thanks to this, they typically return highly interpretable proofs accessible to human readers. Baselines in this category generally include symbolic engines equipped with human-designed heuristics. For example, Chou et al. provided 18 heuristics such as “If OA ⊥ OB and OA = OB, construct C on the opposite ray of OA such that OC = OA*”*, besides 75 deduction rules for the symbolic engine. Large language models^22–24^ such as GPT-4 (ref. ^25^) can be considered to be in this category. Large language models have demonstrated remarkable reasoning ability on a variety of reasoning tasks^26–29^. When producing full natural-language proofs on IMO-AG-30, however, GPT-4 has a success rate of 0%, often making syntactic and semantic errors throughout its outputs, showing little understanding of geometry knowledge and of the problem statements itself. Note that the performance of GPT-4 performance on IMO problems can also be contaminated by public solutions in its training data. A better GPT-4 performance is therefore still not comparable with other solvers. In general, search methods have no theoretical guarantee in their proving performance and are known to be weaker than computer algebra methods^13^.

### Synthetic data generation rediscovers known theorems and beyond

**Fig. 4:**
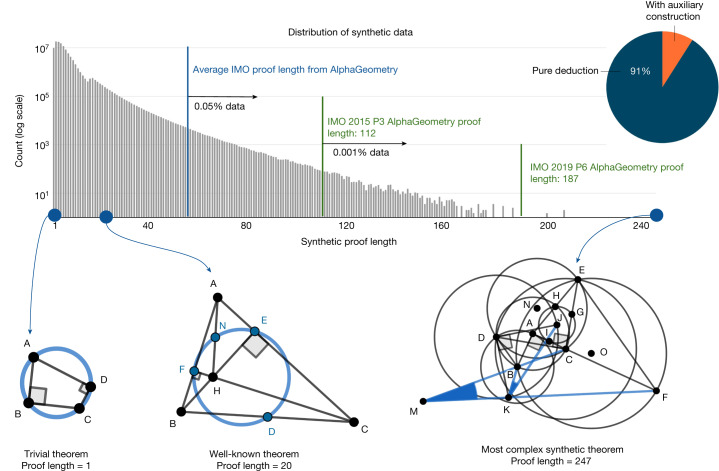
Analysis of the generated synthetic data. Of the generated synthetic proofs, 9% are with auxiliary constructions. Only roughly 0.05% of the synthetic training proofs are longer than the average AlphaGeometry proof for the test-set problems. The most complex synthetic proof has an impressive length of 247 with two auxiliary constructions. Most synthetic theorem premises tend not to be symmetrical like human-discovered theorems, as they are not biased towards any aesthetic standard. Source Data

We find that our synthetic data generation can rediscover some fairly complex theorems and lemmas known to the geometry literature, as shown in Fig. 4, despite starting from randomly sampled theorem premises. This can be attributed to the use of composite actions described in Extended Data Table 1, such as ‘taking centroid’ or ‘taking excentre’, which—by chance—sampled a superset of well-known theorem premises, under our large-scale exploration setting described in Methods. To study the complexity of synthetic proofs, Fig. 4 shows a histogram of synthetic proof lengths juxtaposed with proof lengths found on the test set of olympiad problems. Although the synthetic proof lengths are skewed towards shorter proofs, a small number of them still have lengths up to 30% longer than the hardest problem in the IMO test set. We find that synthetic theorems found by this process are not constrained by human aesthetic biases such as being symmetrical, therefore covering a wider set of scenarios known to Euclidean geometry. We performed deduplication as described in Methods, resulting in more than 100 millions unique theorems and proofs, and did not find any IMO-AG-30 theorems, showing that the space of possible geometry theorems is still much larger than our discovered set.

### Language model pretraining and fine-tuning

We first pretrained the language model on all 100 million synthetically generated proofs, including ones of pure symbolic deduction. We then fine-tuned the language model on the subset of proofs that requires auxiliary constructions, accounting for roughly 9% of the total pretraining data, that is, 9 million proofs, to better focus on its assigned task during proof search.

## Proving results on IMO-AG-30

The performance of ten different solvers on the IMO-AG-30 benchmark is reported in Table 1, of which eight, including AlphaGeometry, are search-based methods. Besides prompting GPT-4 to produce full proofs in natural language with several rounds of reflections and revisions, we also combine GPT-4 with DD + AR as another baseline to enhance its deduction accuracy. To achieve this, we use detailed instructions and few-shot examples in the prompt to help GPT-4 successfully interface with DD + AR, providing auxiliary constructions in the correct grammar. Prompting details of baselines involving GPT-4 is included in the Supplementary Information.

**Table 1:** Main results on our IMO-AG-30 test benchmark

Method		Problems solved (out of 30)
Computer algebra	Wu’s method^21^ (previous state of the art)	10
	Gröbner basis^20^	4
Search (human-like)	GPT-4 (ref. ^25^)	0
	Full-angle method^30^	2
	Deductive database (DD)^10^	7
	DD + human-designed heuristics^17^	9
	DD + AR (ours)	14
	DD + AR + GPT-4 auxiliary constructions	15
	DD + AR + human-designed heuristics	18
	AlphaGeometry	25
	• Without pretraining	21
	• Without fine-tuning	23

We compare AlphaGeometry to other state-of-the-art methods (computer algebra and search approaches), most notably Wu’s method. We also show the results of DD + AR (our contribution) and its variants, resulting in the strongest baseline DD + AR + human-designed heuristics. Finally, we include ablation settings for AlphaGeometry without pretraining and fine-tuning.

AlphaGeometry achieves the best result, with 25 problems solved in total. The previous state of the art (Wu’s method) solved ten problems, whereas the strongest baseline (DD + AR + human-designed heuristics) solved 18 problems, making use of the algebraic reasoning engine developed in this work and the human heuristics designed by Chou et al.^17^. To match the test time compute of AlphaGeometry, this strongest baseline makes use of 250 parallel workers running for 1.5 h, each attempting different sets of auxiliary constructions suggested by human-designed heuristics in parallel, until success or timeout. Other baselines such as Wu’s method or the full-angle method are not affected by parallel compute resources as they carry out fixed, step-by-step algorithms until termination.

Measuring the improvements made on top of the base symbolic deduction engine (DD), we found that incorporating algebraic deduction added seven solved problems to a total of 14 (DD + AR), whereas the language model’s auxiliary construction remarkably added another 11 solved problems, resulting in a total of 25. As reported in Extended Data Fig. 6, we find that, using only 20% of the training data, AlphaGeometry still achieves state-of-the-art results with 21 problems solved. Similarly, using less than 2% of the search budget (beam size of 8 versus 512) during test time, AlphaGeometry can still solve 21 problems. On a larger and more diverse test set of 231 geometry problems, which covers textbook exercises, regional olympiads and famous theorems, we find that baselines in Table 1 remain at the same performance rankings, with AlphaGeometry solving almost all problems (98.7%), whereas Wu’s method solved 75% and DD + AR + human-designed heuristics solved 92.2%, as reported in Extended Data Fig. 6b.

**Fig. 5:**
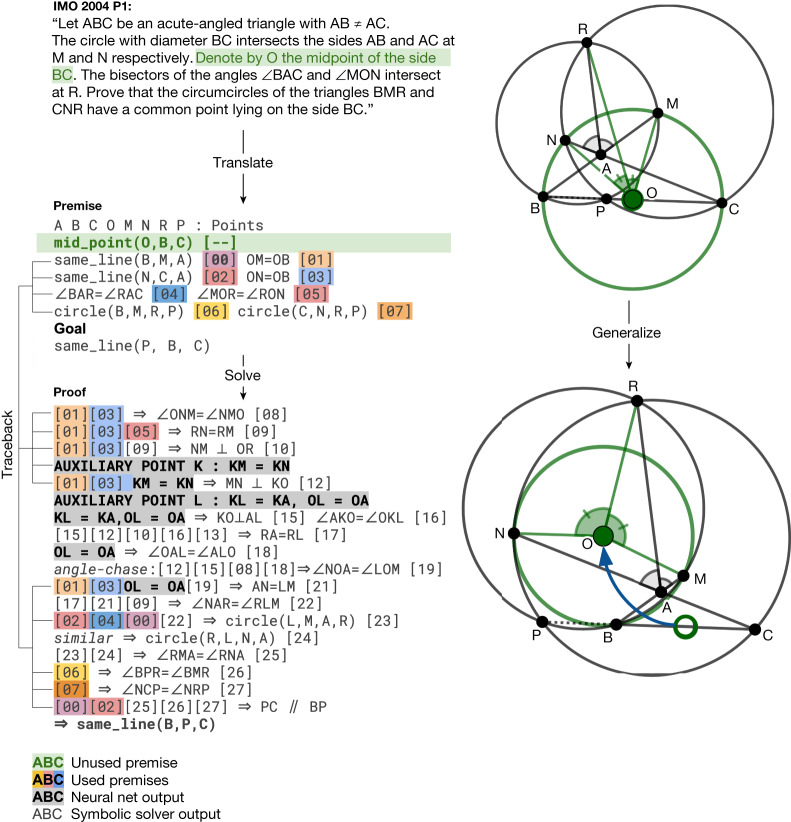
AlphaGeometry discovers a more general theorem than the translated IMO 2004 P1. Left, top to bottom, the IMO 2004 P1 stated in natural language, its translated statement and AlphaGeometry solution. Thanks to the traceback algorithm necessary to extract the minimal premises, AlphaGeometry identifies a premise unnecessary for the proof to work: O does not have to be the midpoint of BC for P, B, C to be collinear. Right, top, the original theorem diagram; bottom, the generalized theorem diagram, in which O is freed from its midpoint position and P still stays on line BC. Note that the original problem requires P to be between B and C, a condition where the generalized theorem and solution does not guarantee.

Notably, AlphaGeometry solved both geometry problems of the same year in 2000 and 2015, a threshold widely considered difficult to the average human contestant at the IMO. Further, the traceback process of AlphaGeometry found an unused premise in the translated IMO 2004 P1, as shown in Fig. 5, therefore discovering a more general version of the translated IMO theorem itself. We included AlphaGeometry solutions to all problems in IMO-AG-30 in the Supplementary Information and manually analysed some notable AlphaGeometry solutions and failures in Extended Data Figs. 2–5. Overall, we find that AlphaGeometry operates with a much lower-level toolkit for proving than humans do, limiting the coverage of the synthetic data, test-time performance and proof readability.

### Human expert evaluation of AlphaGeometry outputs

**Extended Data Fig. 1:**
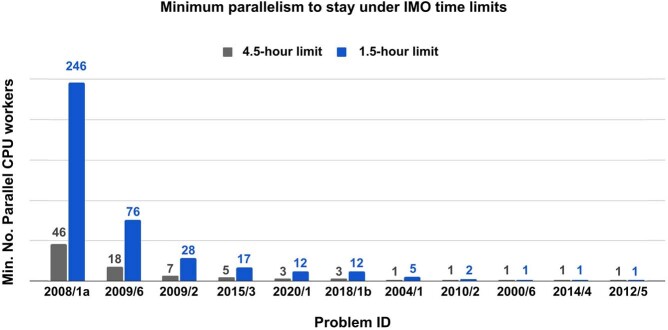
The minimum number of parallel CPU workers to solve all 25 problems and stay under the time limit, given four parallel copies of the GPU V100-accelerated language model. Each problem has a different running time resulting from their unique size of the deduction closure. We observed that running time does not correlate with the difficulty of the problem. For example, IMO 2019 P6 is much harder than IMO 2008 P1a, yet it requires far less parallelization to reach a solution within IMO time limits. Source Data

**Extended Data Fig. 2:**
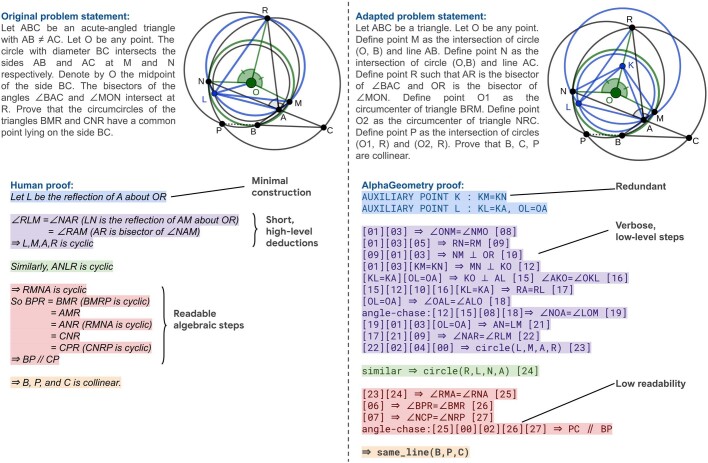
Side-by-side comparison of AlphaGeometry proof versus human proof on the translated IMO 2004 P1. Both the AlphaGeometry and human solutions recognize the axis of symmetry between M and N through O. AlphaGeometry constructs point K to materialize this axis, whereas humans simply use the existing point R for the same purpose. This is a case in which proof pruning itself cannot remove K and a sign of similar redundancy in our synthetic data. To prove five-point concyclicity, AlphaGeometry outputs very lengthy, low-level steps, whereas humans use a high-level insight (OR is the symmetrical axis of both LN and AM) to obtain a broad set of conclusions all at once. For algebraic deductions, AlphaGeometry cannot flesh out its intermediate derivations, which is implicitly carried out by Gaussian elimination, therefore leading to low readability. Overall, this comparison points to the use of higher-level tools to improve the synthetic data, proof search and readability of AlphaGeometry. Note that in the original IMO 2004 P1, the point P is proven to be between B and C. The generalized version needs further contraints on the position of O to satisfy this betweenness requirement.

**Extended Data Fig. 3:**
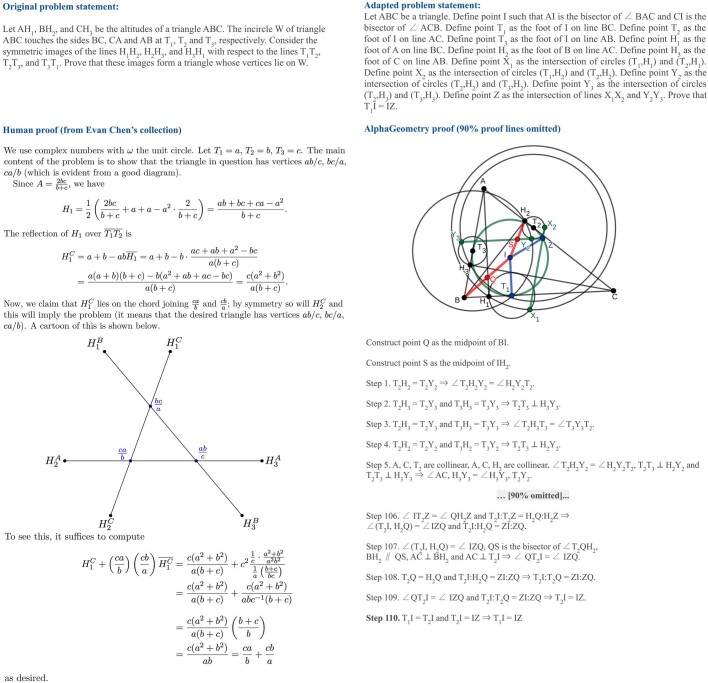
Side-by-side comparison of human proof and AlphaGeometry proof for the IMO 2000 P6. This is a harder problem (average human score = 1.05/7), with a large number of objects in the problem statements, resulting in a very crowded diagram. Left, the human solution uses complex numbers. With a well-chosen coordinate system, the problem is greatly simplified and a solution follows naturally through algebraic manipulation. Right, AlphaGeometry solution involves two auxiliary constructions and more than 100 deduction steps, with many low-level steps that are extremely tedious to a human reader. This is a case in which the search-based solution is much less readable and much less intuitive than coordinate bashing. A more structural organization, that is, a high-level proof outline, can improve readability of the AlphaGeometry solution substantially. Again, this suggests building into AlphaGeometry many higher-level deduction rules to encapsulate large groups of low-level deductions into fewer proof steps.

**Extended Data Fig. 4:**
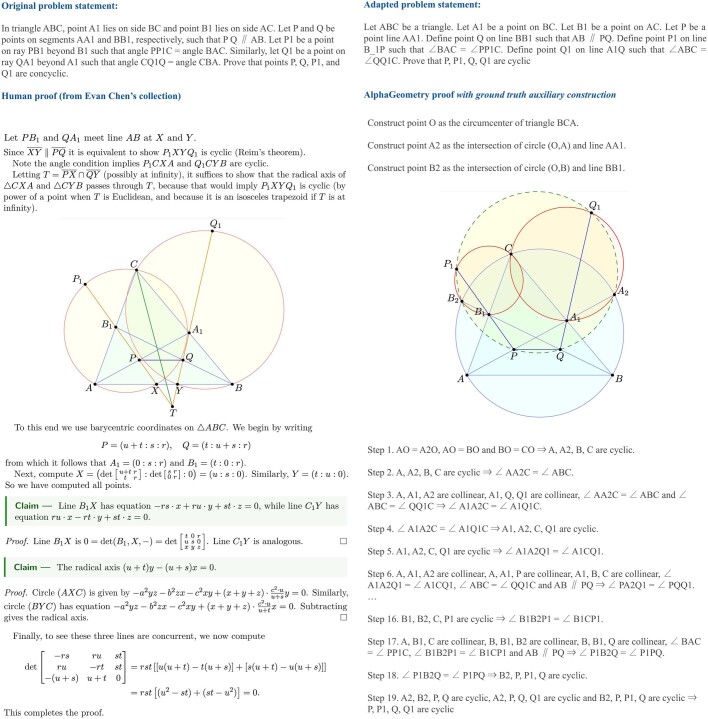
Side-by-side comparison of human proof and AlphaGeometry proof for the IMO 2019 P2. This is one out of five unsolved problems by AlphaGeometry. Left, the human solution uses both auxiliary constructions and barycentric coordinates. With a well-chosen coordinate system, a solution becomes available through advanced algebraic manipulation. Right, AlphaGeometry solution when provided with the ground-truth auxiliary construction for a synthetic proof. This auxiliary construction can be found quickly with the knowledge of Reim’s theorem, which is not included in the deduction rule list used by the symbolic engine during synthetic data generation. Including such high-level theorems into the synthetic data generation can greatly improve the coverage of synthetic data and thus improve auxiliary construction capability. Further, higher-level steps using Reim’s theorem also cut down the current proof length by a factor of 3.

**Extended Data Fig. 5:**
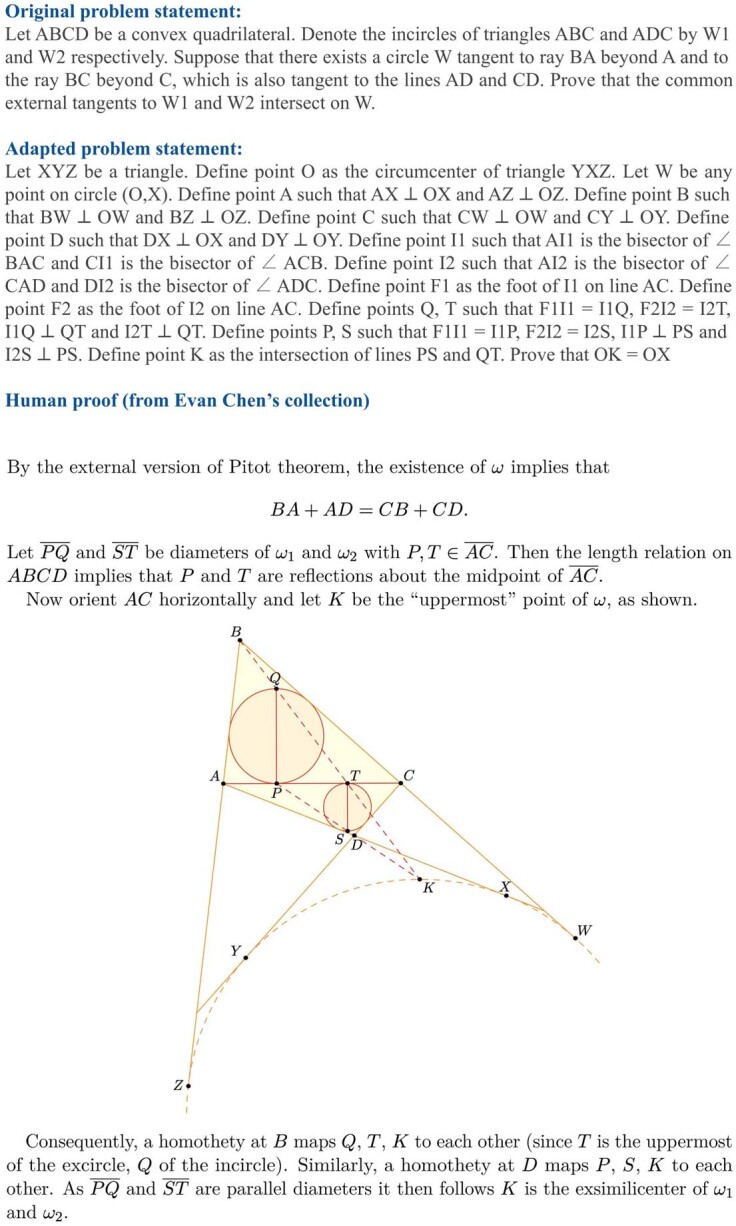
Human proof for the IMO 2008 P6. This is an unsolved problem by AlphaGeometry and also the hardest one among all 30 problems, with an average human score of only 0.28/7. This human proof uses four auxiliary constructions (diameters of circles W1 and W2) and high-level theorems such as the Pitot theorem and the notion of homothety. These high-level concepts are not available to our current version of the symbolic deduction engine both during synthetic data generation and proof search. Supplying AlphaGeometry with the auxiliary constructions used in this human proof also does not yield any solution. There is also no guarantee that a synthetic solution exists for AlphaGeometry, across all possible auxiliary constructions, without enhancing its symbolic deduction with more powerful rules. Again, this suggests that enhancing the symbolic engine with more powerful tools that IMO contestants are trained to use can improve both the synthetic data and the test-time performance of AlphaGeometry.

Because AlphaGeometry outputs highly interpretable proofs, we used a simple template to automatically translate its solutions to natural language. To obtain an expert evaluation in 2000 and 2015, during which AlphaGeometry solves all geometry problems and potentially passes the medal threshold, we submit these solutions to the USA IMO team coach, who is experienced in grading mathematical olympiads and has authored books for olympiad geometry training. AlphaGeometry solutions are recommended to receive full scores, thus passing the medal threshold of 14/42 in the corresponding years. We note that IMO tests also evaluate humans under three other mathematical domains besides geometry and under human-centric constraints, such as no calculator use or 4.5-h time limits. We study time-constrained settings with 4.5-h and 1.5-h limits for AlphaGeometry in Methods and report the results in Extended Data Fig. 1.

### Learning to predict the symbolic engine’s output improves the language model’s auxiliary construction

In principle, auxiliary construction strategies must depend on the details of the specific deduction engine they work with during proof search. We find that a language model without pretraining only solves 21 problems. This suggests that pretraining on pure deduction proofs generated by the symbolic engine DD + AR improves the success rate of auxiliary constructions. On the other hand, a language model without fine-tuning also degrades the performance but not as severely, with 23 problems solved compared with AlphaGeometry’s full setting at 25.

### Hard problems are reflected in AlphaGeometry proof length

**Fig. 6:**
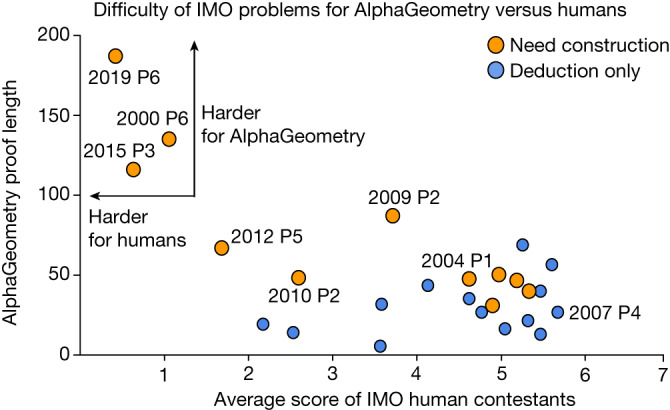
AlphaGeometry proof length versus the average score of IMO participants on different problems. Among the solved problems, 2000 P6, 2015 P3 and 2019 P6 are the hardest for IMO participants. They also require the longest proofs from AlphaGeometry. For easier problems, however, there is little correlation between AlphaGeometry proof length and human score. Source Data

Figure 6 measures the difficulty of solved problems using public scores of human contestants at the IMO and plots them against the corresponding AlphaGeometry proof lengths. The result shows that, for the three problems with the lowest human score, AlphaGeometry also requires exceptionally long proofs and the help of language-model constructions to reach its solution. For easier problems (average human score > 3.5), however, we observe no correlation (*p* = −0.06) between the average human score and AlphaGeometry proof length.

## Conclusion

AlphaGeometry is the first computer program to surpass the performance of the average IMO contestant in proving Euclidean plane geometry theorems, outperforming strong computer algebra and search baselines. Notably, we demonstrated through AlphaGeometry a neuro-symbolic approach for theorem proving by means of large-scale exploration from scratch, sidestepping the need for human-annotated proof examples and human-curated problem statements. Our method to generate and train language models on purely synthetic data provides a general guiding framework for mathematical domains that are facing the same data-scarcity problem.

## Methods

### Geometry representation

General-purpose formal languages such as Lean^31^ still require a large amount of groundwork to describe most IMO geometry problems at present. We do not directly address this challenge as it requires deep expertise and substantial research outside the scope of theorem-proving methodologies. To sidestep this barrier, we instead adopted a more specialized language used in GEX^10^, JGEX^17^, MMP/Geometer^13^ and GeoLogic^19^, a line of work that aims to provide a logical and graphical environment for synthetic geometry theorems with human-like non-degeneracy and topological assumptions. Examples of this language are shown in Fig. 1d,f. Owing to its narrow formulation, 75% of all IMO geometry problems can be adapted to this representation. In this type of geometry environment, each proof step is logically and numerically verified and can also be evaluated by a human reader as if it is written by IMO contestants, thanks to the highly natural grammar of the language. To cover more expressive algebraic and arithmetic reasoning, we also add integers, fractions and geometric constants to the vocabulary of this language. We do not push further for a complete solution to geometry representation as it is a separate and extremely challenging research topic that demands substantial investment from the mathematical formalization community.

### Sampling consistent theorem premises

We developed a constructive diagram builder language similar to that used by JGEX^17^ to construct one object in the premise at a time, instead of freely sampling many premises that involve several objects, therefore avoiding the generation of a self-contradicting set of premises. An exhaustive list of construction actions is shown in Extended Data Table 1. These actions include constructions to create new points that are related to others in a certain way, that is, collinear, incentre/excentre etc., as well as constructions that take a number as its parameter, for example, “construct point X such that given a number *α*, ∠ABX = *α*”. One can extend this list with more sophisticated actions to describe a more expressive set of geometric scenarios, improving both the synthetic data diversity and the test-set coverage. A more general and expressive diagram builder language can be found in ref. ^32^. We make use of a simpler language that is sufficient to describe problems in IMO-AG-30 and can work well with the symbolic engine DD.

### The symbolic deduction engine

The core functionality of the engine is deducing new true statements given the theorem premises. Deduction can be performed by means of geometric rules such as ‘If X then Y’, in which X and Y are sets of geometric statements such as ‘A, B, C are collinear’. We use the method of structured DD^10,17^ for this purpose as it can find the deduction closure in just seconds on standard non-accelerator hardware. To further enhance deduction, we also built into AlphaGeometry the ability to perform deduction through AR. AR enable proof steps that perform angle/ratio/distance chasing. Detailed examples of AR are shown in Extended Data Table 2. Such proof steps are ubiquitous in geometry proofs, yet not covered by geometric rules. We expand the Gaussian elimination process implemented in GeoLogic^19^ to find the deduction closure for all possible linear operators in just seconds. Our symbolic deduction engine is an intricate integration of DD and AR, which we apply alternately to expand the joint closure of known true statements until expansion halts. This process typically finishes within a few seconds to at most a few minutes on standard non-accelerator hardware.

### Algebraic reasoning

There has not been a complete treatment for algebraic deduction in the literature of geometry theorem proving. For example, in iGeoTutor^12^, Z3 (ref. ^33^) is used to handle arithmetic inferences but algebraic manipulations are not covered. DD (ref. ^17^) handles algebraic deductions by expressing them under a few limited deduction rules, therefore, it is unable to express more complex manipulations, leaving arithmetic inferences not covered. The most general treatment so far is a process similar that in ref. ^34^ for angle-only theorem discovery and implemented in GeoLogic^19^ for both angle and ratios. We expanded this formulation to cover all reasoning about angles, ratios and distances between points and also arithmetic reasoning with geometric constants such as ‘pi’ or ‘1:2’. Concrete examples of algebraic reasoning are given in Extended Data Table 2.

On a high level, we first convert the input linear equations to a matrix of their coefficients. In particular, we create a coefficient matrix *A* ∈ *R*^*M*×*N*^ in which *N* is the number of variables and *M* is the number of input equations. In geometry, any equality is of the form *a* − *b* = *c* − *d* ⇔ *a* − *b* − *c* + *d* = 0. For example, the angle equality ∠ABC = ∠XYZ is represented as *s*(AB) − *s*(BC) = *s*(XY) − *s*(YZ), in which *s*(AB) is the angle between AB and the x-direction, modulo pi. Similarly, ratios AB:CD = EF:GH are represented as log(AB) − log(CD) = log(EF) − log(GH), in which log(AB) is the log of the length of segment AB. For distances, each variable is a (point, line) pair, representing a specific point on a specific line.

Because all equalities are of the form ‘*a* − *b* − *c* + *d* = 0’, we populate the row for each equality with values +1, −1, −1, +1 at columns corresponding to variables *a*, *b*, *c* and *d*. Running Gaussian elimination on A returns a new matrix with leading 1s at each of the columns, essentially representing each variable as a unique linear combination of all remaining variables. As an example, suppose we have ‘*a* − *b* = *b* − *c*’, ‘*d* − *c* = *a* − *d*’ and ‘*b* − *c* = *c* − *e*’ as input equalities, running the Gaussian elimination process (denoted GE in the following equation) returns the following result:

$$(\begin{array}{ccccc}a & b & c & d & e\\ 1 & -2 & 1 & 0 & 0\\ -1 & 0 & -1 & 2 & 0\\ 0 & 1 & -2 & 0 & 1\end{array})\,\mathop{\to }\limits^{GE}(\begin{array}{ccccc}a & b & c & d & e\\ 1 & 0 & 0 & -1.5 & 0.5\\ 0 & 1 & 0 & -1 & 0\\ 0 & 0 & 1 & -0.5 & -0.5\end{array})\Rightarrow \{\begin{array}{c}a=1.5d-0.5e\\ b=d\\ c=0.5d+0.5e\end{array}$$

From this result, we can deterministically and exhaustively deduce all new equalities by checking if *x*_1_ = *x*_2_ or *x*_1_ − *x*_2_ = *x*_2_ − *x*_3_ or *x*_1_ − *x*_2_ = *x*_3_ − *x*_4_, in which {*x*_1_, *x*_2_, *x*_3_, *x*_4_} is any 4-permutation of all variables. In the above Gaussian Elimination, for example, AR deduced that *b* = *d* from the three input equalities. To handle geometric constants such as ‘0.5 pi’ or ‘5:12’, we included ‘pi’ and ‘1’ as default variables to all coefficient matrices.

### Deductive database implementation

Unlike the original implementation of DD, we use a graph data structure to capture the symmetries of geometry, rather than using strings of canonical forms. With a graph data structure, we captured not only the symmetrical permutations of function arguments but also the transitivity of equality, collinearity and concyclicity. This graph data structure bakes into itself some deduction rules explicitly stated in the geometric rule list used in DD. These deduction rules from the original list are therefore not used anywhere in exploration but implicitly used and explicitly spelled out on-demand when the final proof is serialized into text.

#### Traceback to find minimal proofs

Each deduction step needs to be coupled with a traceback algorithm, which returns the minimal set of immediate ancestor statements that is necessary to deduce the conclusion statement of the step. This is the core building block for extracting proof graphs and minimal premises described in the main text. A minimal-premise-extraction algorithm is necessary to avoid superfluous auxiliary constructions that contribute to the proof through unnecessary transitivity. For example, ‘*a* = *b*’ and ‘*b* = *c*’ might not be necessary if ‘*a* = *c*’ can be obtained directly through other reasoning chains.

### Traceback for geometric-rule deduction

To do this, we record the equality transitivity graph. For example, if ‘*a* = *b*’, ‘*b* = *c*’, ‘*c* = *d*’ and ‘*a* = *d*’ are deduced, which results in nodes *a*, *b*, *c* and *d* being connected to the same ‘equality node’ *e*, we maintain a graph within *e* that has edges [(*a*, *b*), (*b*, *c*), (*c*, *d*), (*a*, *d*)]. This allows the traceback algorithm to perform a breadth-first search to find the shortest path of transitivity of equality between any pair of variables among *a*, *b*, *c* and *d*. For collinearity and concyclicity, however, the representation is more complex. In these cases, hypergraphs *G*(*V*, *E*) with 3-edges or 4-edges are used as the equality transitivity graph. The traceback is now equivalent to finding a minimum spanning tree (denoted MST in the following equation) for the target set *S* of nodes (three collinear nodes or four concyclic nodes) whose weight is the cardinality of the union of its hyperedges *e*′:

$${\rm{MST}}(S)={\min }_{T\subset E}| {\bigcup }_{{e}^{{\prime} }\subset T}w({e}^{{\prime} })| \,{\rm{s.t.}}\,S\subset T$$

Such optimization is NP-hard, as it is a reduction from the decision version of vertex cover. We simply use a greedy algorithm in this case to find a best-effort minimum spanning tree.

### Traceback for algebraic deduction

Traceback through Gaussian elimination can be done by recognizing that it is equivalent to a mixed integer linear programming problem. Given the coefficient matrix of input equations *A* constructed as described in the previous sections and a target equation with coefficients vector *b* ∈ *R*^*N*^, we determine the minimal set of premises for *b* by defining non-negative integer decision vectors *x*, *y* ∈ *Z*^*M*^ and solve the following mixed-integer linear programming problem:

$$x,y={\min }_{x,y}{\sum }_{i}\left({x}_{i}+{y}_{i}\right)\,{\rm{s.t.}}\,{A}^{{\rm{T}}}\left(x-y\right)=b$$

The minimum set of immediate parent nodes for the equality represented by *b* will be the *i*th equations (*i*th rows in *A*) whose corresponding decision value (*x*_*i*_ − *y*_*i*_) is non-zero.

### Integrating DD and AR

DD and AR are applied alternately to expand their joint deduction closure. The output of DD, which consists of new statements deduced with deductive rules, is fed into AR and vice versa. For example, if DD deduced ‘AB is parallel to CD’, the slopes of lines AB and CD will be updated to be equal variables in AR’s coefficient matrix *A*, defined in the ‘Algebraic reasoning’ section. Namely, a new row will be added to *A* with ‘1’ at the column corresponding to the variable slope(AB) and ‘−1’ at the column of slope(CD). Gaussian elimination and mixed-integer linear programming is run again as AR executes, producing new equalities as inputs to the next iteration of DD. This loop repeats until the joint deduction closure stops expanding. Both DD and AR are deterministic processes that only depend on the theorem premises, therefore they do not require any design choices in their implementation.

### Proof pruning

Although the set of immediate ancestors to any node is minimal, this does not guarantee that the fully traced back dependency subgraph *G*(*N*) and the necessary premise *P* are minimal. Here we define minimality to be the property that *G*(*N*) and *P* cannot be further pruned without losing conclusion reachability. Without minimality, we obtained many synthetic proofs with vacuous auxiliary constructions, having shallow relation to the actual proof and can be entirely discarded. To solve this, we perform exhaustive trial and error, discarding each subset of the auxiliary points and rerunning DD + AR on the smaller subset of premises to verify goal reachability. At the end, we return the minimum proof obtainable across all trials. This proof-pruning procedure is done both during synthetic data generation and after each successful proof search during test time.

### Parallelized data generation and deduplication

We run our synthetic-data-generation process on a large number of parallel CPU workers, each seeded with a different random seed to reduce duplications. After running this process on 100,000 CPU workers for 72 h, we obtained roughly 500 million synthetic proof examples. We reformat the proof statements to their canonical form (for example, sorting arguments of individual terms and sorting terms within the same proof step, etc.) to avoid shallow deduplication against itself and against the test set. At the end, we obtain 100 million unique theorem–proof examples. A total of 9 million examples involves at least one auxiliary construction. We find no IMO-AG-30 problems in the synthetic data. On the set of geometry problems collected in JGEX^17^, which consists mainly of problems with moderate difficulty and well-known theorems, we find nearly 20 problems in the synthetic data. This suggests that the training data covered a fair amount of common knowledge in geometry, but the space of more sophisticated theorems is still much larger.

### Language model architecture and training

We use the Meliad library^35^ for transformer training with its base settings. The transformer has 12 layers, embedding dimension of 1,024, eight heads of attention and an inter-attention dense layer of dimension 4,096 with ReLU activation. Overall, the transformer has 151 million parameters, excluding embedding layers at its input and output heads. Our customized tokenizer is trained with ‘word’ mode using SentencePiece^36^ and has a vocabulary size of 757. We limit the maximum context length to 1,024 tokens and use T5-style relative position embedding^37^. Sequence packing^38,39^ is also used because more than 90% of our sequences are under 200 in length. During training, a dropout^40^ rate of 5% is applied pre-attention and post-dense. A 4 × 4 slice of TPUv3 (ref. ^41^) is used as its hardware accelerator. For pretraining, we train the transformer with a batch size of 16 per core and a cosine learning-rate schedule that decays from 0.01 to 0.001 in 10,000,000 steps. For fine-tuning, we maintain the final learning rate of 0.001 for another 1,000,000 steps. For the set-up with no pretraining, we decay the learning rate from 0.01 to 0.001 in 1,000,000 steps. We do not perform any hyperparameter tuning. These hyperparameter values are either selected to be a large round number (training steps) or are provided by default in the Meliad codebase.

#### Parallelized proof search

Because the language model decoding process returns *k* different sequences describing *k* alternative auxiliary constructions, we perform a beam search over these *k* options, using the score of each beam as its value function. This set-up is highly parallelizable across beams, allowing substantial speed-up when there are parallel computational resources. In our experiments, we use a beam size of *k* = 512, the maximum number of iterations is 16 and the branching factor for each node, that is, the decoding batch size, is 32. This is the maximum inference-time batch size that can fit in the memory of a GPU V100 for our transformer size. Scaling up these factors to examine a larger fraction of the search space might improve AlphaGeometry results even further.

For each problem, we used a pool of four GPU workers, each hosting a copy of the transformer language model to divide the work between alternative beams, and a pool of 10,000 CPU workers to host the symbolic solvers, shared across all beams across all 30 problems. This way, a problem that terminates early can contribute its share of computing power to longer-running problems. We record the running time of the symbolic solver on each individual problem, which—by design—stays roughly constant across all beams. We use this and the language model decoding speed to infer the necessary parallelism needed for each problem, in isolation, to stay under different time limits at the IMO in Extended Data Fig. 1.

### The effect of data and search

**Extended Data Fig. 6:**
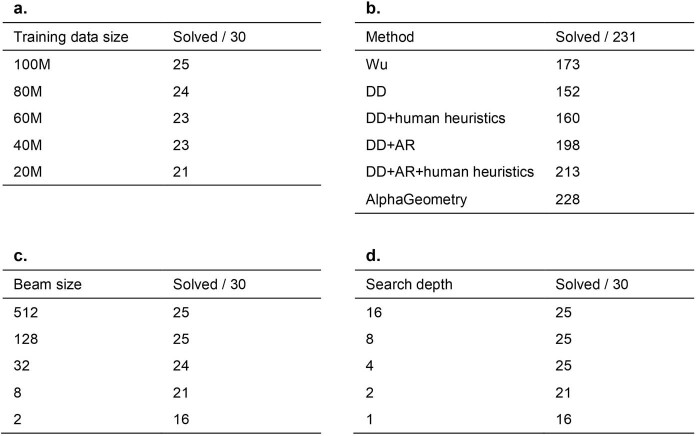
Analysis of AlphaGeometry performance under changes made to its training and testing. **a**, The effect of reducing training data on AlphaGeometry performance. At 20% of training data, AlphaGeometry still solves 21 problems, outperforming all other baselines. **b**, Evaluation on a larger set of 231 geometry problems, covering a diverse range of sources outside IMO competitions. The rankings of different machine solvers stays the same as in Table 1, with AlphaGeometry solving almost all problems. **c**, The effect of reducing beam size during test time on AlphaGeometry performance. At beam size 8, that is, a 64 times reduction from its full setting, AlphaGeometry still solves 21 problems, outperforming all other baselines. **d**, The effect of reducing search depth on AlphaGeometry performance. At depth 2, AlphaGeometry still solves 21 problems, outperforming all other baselines.

We trained AlphaGeometry on smaller fractions of the original training data (20%, 40%, 60% and 80%) and found that, even at 20% of training data, AlphaGeometry still solves 21 problems, more than the strongest baseline (DD + AR + human-designed heuristics) with 18 problems solved, as shown in Extended Data Fig. 6a. To study the effect of beam search on top of the language model, we reduced the beam size and search depth separately during proof search and reported the results in Extended Data Fig. 6c,d. We find that, with a beam size of 8, that is, a 64 times reduction from the original beam size of 512, AlphaGeometry still solves 21 problems. A similar result of 21 problems can be obtained by reducing the search depth from 16 to only two, while keeping the beam size constant at 512.

### Evaluation on a larger test set

We evaluated AlphaGeometry and other baselines on a larger test set of 231 geometry problems, curated in ref. ^17^. This set covers a wider range of sources outside IMO competitions: textbook examples and exercises, regional olympiads and famous geometry theorems; some are even more complex than typical IMO problems, such as the five circles theorem, Morley’s theorem or Sawayama and Thébault’s theorem. The results are reported in Extended Data Fig. 6b. The overall rankings of different approaches remained the same as in Table 1, with AlphaGeometry solving almost all problems (98.7%). The strongest baseline DD + AR + human-designed heuristics solves 92.2%, whereas the previous state of the art solves 75%.

#### AlphaGeometry framework and applicability to other domains

The strength of AlphaGeometry’s neuro-symbolic set-up lies in its ability to generate auxiliary constructions, which is an important ingredient across many mathematical domains. In Extended Data Table 3, we give examples in four other mathematical domains in which coming up with auxiliary constructions is key to the solution. In Extended Data Table 4, we give a line-by-line comparison of a geometry proof and an inequality proof for the IMO 1964 Problem 2, highlighting how they both fit into the same framework.

Our paper shows that language models can learn to come up with auxiliary constructions from synthetic data, in which problem statements and auxiliary constructions are randomly generated together and then separated using the traceback algorithm to identify the dependency difference. Concretely, the AlphaGeometry framework requires the following ingredients:

1. An implementation of the domain’s objects and definitions.
2. A random premise sampler.
3. The symbolic engine(s) that operate within the implementation (1).
4. A traceback procedure for the symbolic engine.

Using these four ingredients and the algorithm described in the main text, one can generate synthetic data for any target domain. As shown in our paper, there are non-trivial engineering challenges in building each ingredient. For example, current formalizations of combinatorics are very nascent, posing challenges to (1) and (2). Also, building powerful symbolic engines for different domains requires deep domain expertise, posing challenges to (3) and (4). We consider applying this framework to a wider scope as future work and look forward to further innovations that tackle these challenges.

### Transformer in theorem proving

Research in automated theorem proving has a long history dating back to the 1950s (refs. ^6,42,43^), resulting in highly optimized first-order logic solvers such as E (ref. ^44^) or Vampire^45^. In the 2010s, deep learning matured as a new powerful tool for automated theorem proving, demonstrating great successes in premise selection and proof guidance^46–49^, as well as SAT solving^50^. On the other hand, transformer^18^ exhibits outstanding reasoning capabilities across a variety of tasks^51–53^. The first success in applying transformer language models to theorem proving is GPT-f (ref. ^15^). Its follow up extensions^2,16^ further developed this direction, allowing machines to solve some olympiad-level problems for the first time. Innovation in the proof-search algorithm and online training^3^ also improves transformer-based methods, solving a total of ten (adapted) IMO problems in algebra and number theory. These advances, however, are predicated on a substantial amount of human proof examples and standalone problem statements designed and curated by humans.

#### Geometry theorem proving

Geometry theorem proving evolves in an entirely separate space. Its literature is divided into two branches, one of computer algebra methods and one of search methods. The former is largely considered solved since the introduction of Wu’s method^21^, which can theoretically decide the truth value of any geometrical statement of equality type, building on specialized algebraic tools introduced in earlier works^54,55^. Even though computer algebra has strong theoretical guarantees, its performance can be limited in practice owing to their large time and space complexity^56^. Further, the methodology of computer algebra is not of interest to AI research, which instead seeks to prove theorems using search methods, a more human-like and general-purpose process.

Search methods also started as early as the 1950s (refs. ^6,7^) and continued to develop throughout the twentieth century^57–60^. With the introduction of DD^10,17^, area methods^61^ and full-angle methods^30^, geometry solvers use higher-level deduction rules than Tarski’s or Hilbert’s axioms and are able to prove a larger number of more complex theorems than those operating in formal languages. Geometry theorem proving of today, however, is still relying on human-designed heuristics for auxiliary constructions^10–14^. Geometry theorem proving falls behind the recent advances made by machine learning because its presence in formal mathematical libraries such as Lean^31^ or Isabelle^62^ is extremely limited.

#### Synthetic data in theorem proving

Synthetic data has long been recognized and used as an important ingredient in theorem proving^63–66^. State-of-the-art machine learning methods make use of expert iteration to generate a curriculum of synthetic proofs^2,3,15^. Their methods, however, only generate synthetic proofs for a fixed set of predefined problems, designed and selected by humans. Our method, on the other hand, generates both synthetic problems and proofs entirely from scratch. Aygun et al.^67^ similarly generated synthetic proofs with hindsight experience replay^68^, providing a smooth range of theorem difficulty to aid learning similar to our work. AlphaGeometry, however, is not trained on existing conjectures curated by humans and does not learn from proof attempts on the target theorems. Their approach is thus orthogonal and can be used to further improve AlphaGeometry. Most similar to our work is Firoiu et al.^69^, whose method uses a forward proposer to generate synthetic data by depth-first exploration and trains a neural network purely on these synthetic data. Our work, on the other hand, uses breadth-first exploration, necessary to obtain the minimal proofs and premises, and uses a traceback algorithm to identify auxiliary constructions, thus introducing new symbols and hypotheses that the forward proposer cannot propose.

## Online content

Any methods, additional references, Nature Portfolio reporting summaries, source data, extended data, supplementary information, acknowledgements, peer review information; details of author contributions and competing interests; and statements of data and code availability are available at 10.1038/s41586-023-06747-5.

### Supplementary information

Supplementary Information Supplementary Sections 1 and 2. Section 1 contains GPT-4 prompting details and includes prompting for two scenarios: (1) GPT-4 producing full proofs in natural language and (2) GPT-4 interfaces with DD + AR. Section 2 contains AlphaGeometry solutions for problems in IMO-AG-30. It lists the 30 problem statements, their diagrams to aid understanding and AlphaGeometry solution (if any) sequentially.

### Source data

Source Data Fig. 2

Source Data Fig. 4

Source Data Fig. 6

Source Data Extended Data Fig. 1

## Figures and Tables

**Extended Data Table 1:**
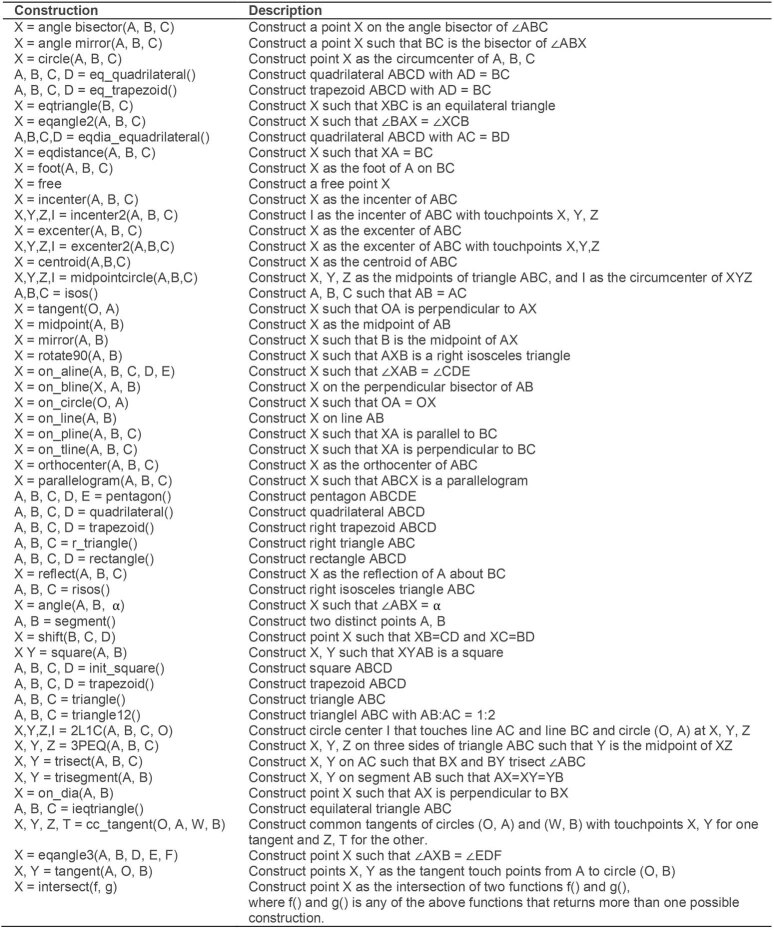
List of actions to construct the random premises

These actions include constructions to create new points that are related to others in a certain way, for example, collinear, incentre/excentre etc., and constructions that take a number as its parameter.

**Extended Data Table 2:**
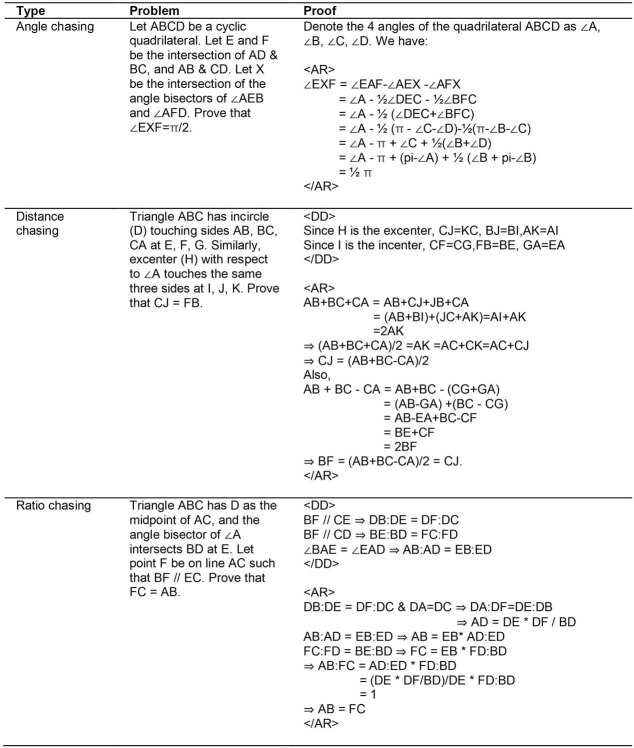
Three examples of algebraic reasoning (AR) in geometry theorem proving, with AR proof steps between the two tags <AR></AR>

In AlphaGeometry, the engine AR can execute all three examples efficiently, under a unified procedure of Gaussian elimination.

**Extended Data Table 3:**
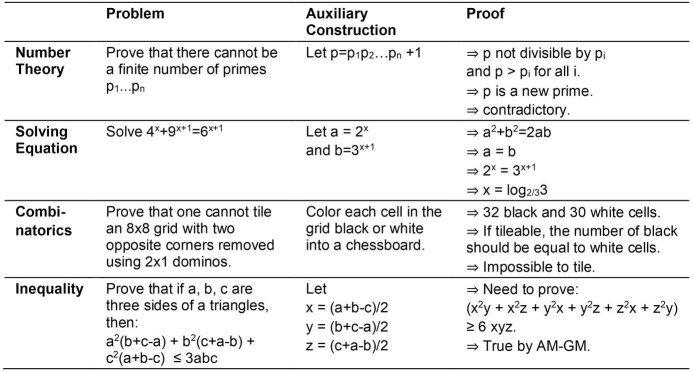
Examples of auxiliary constructions in four different domains

In these examples, the construction is key to the proof, whereas the remaining proof is relatively more mechanical. In AlphaGeometry, the mechanical portion is efficiently handled by the symbolic engine DD + AR.

**Extended Data Table 4:**
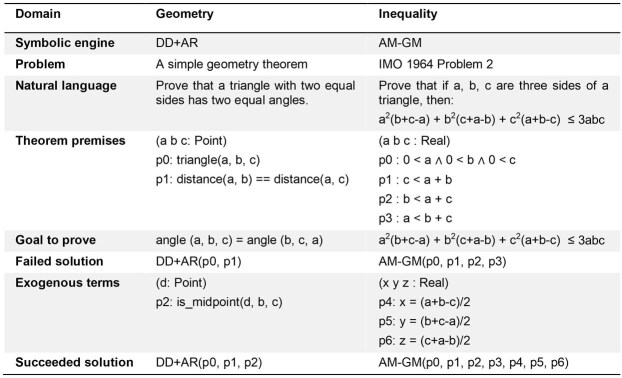
A comparison between a geometry proof and an IMO inequality proof through the lens of the AlphaGeometry framework

We assume AM-GM to be a symbolic engine capable of (1) algebraic rewrites and simplification and (2) applying the inequality rule of arithmetic means–geometric means. With the original premises, directly applying AM-GM fails to deliver a solution, which is similar to the geometry example, for which DD + AR fails to solve the simple problem. Some correct auxiliary constructions are necessary for both symbolic engines (DD + AR in the case of geometry and AM-GM in the case of inequality) to succeed, as shown in the last two rows of the table. Note that there are ten more common inequalities typically used at mathematical olympiads besides AM-GM, just as DD + AR itself encapsulates more than 50 different deduction rules for geometry commonly used at the olympiads.

## Data Availability

The data supporting the findings of this work are available in the Extended Data and the Supplementary Information. Source data are provided with this paper.

## Code availability

Our code and model checkpoint is available at https://github.com/google-deepmind/alphageometry.
