# Author Correction: Solving olympiad geometry without human demonstrations

**DOI:** 10.1038/s41586-024-07115-7

**Published:** 2024-02-23

**Authors:** Trieu H. Trinh, Yuhuai Wu, Quoc V. Le, He He, Thang Luong

**Affiliations:** 1 Google Deepmind, Mountain View, CA USA; 2 Computer Science Department, New York University, New York, NY USA

**Keywords:** Computer science, Computational science

Correction to: *Nature* 10.1038/s41586-023-06747-5 Published online 17 January 2024

In the version of the article initially published, the Acknowledgements section listed the name P. Bak as P. Pak. This has now been corrected in the HTML and PDF versions of the article.

