# Supplementary material for: Solving olympiad geometry without human demonstrations
Source: Nature. 2024 Jan 17;625(7995):476–82. doi: 10.1038/s41586-023-06747-5 (PMC10794143; doi:10.1038/s41586-023-06747-5)
Supplement: Supplementary file 1 — Supplementary Sections 1 and 2. Section 1 contains GPT-4 prompting details and includes prompting for two scenarios: (1) GPT-4 producing full proofs in natural language and (2) GPT-4 interfaces with DD + AR. Section 2 contains AlphaGeometry solutions for problems in IMO-AG-30. It lists the 30 problem statements, their diagrams to aid understanding and AlphaGeometry solution (if any) sequentially. [file 41586_2023_6747_MOESM1_ESM.pdf]

nature portfolio

https://doi.org/10.1038/s41586-023-06747-5

Supplementary information

Solving olympiad geometry without human demonstrations

In the format provided by the authors and unedited

Supplementary Information of Solving Olympiad Geometry without Human Demonstration.

Trieu H. Trinh, Yuhuai Wu, Quoc V. Le, He He, Thang Luong

April 2023

1 GPT-4 prompting details

1.1 GPT-4 prompting for full natural language proofs

System message

```
You are GPT-4, an Artificial General Intelligence, and Olympiad Solver,
     an expert theorem prover designed for Mathematical Olympiads. You
    are participating in the International Mathematical Olympiad (IMO).
     You always provide rigorous, detailed, and complete solution to
    any problem that you are asked. You do not discuss any further
    details other than presenting the exact solution to the problem.
    You may think out loud, step by step, or hierarchically organize
    your solution at higher level subgoals down to lower level proof
    steps whenever necessary.
```

Reflect and revise message

```
Is this solution complete and correct? Please deeply reflect on your
    solution. If incomplete, please continue from where you dropped off
    . If incorrect, please try again and provide a correct solution. If
     you think the solution is complete and correct, simply respond "
    DONE." without adding anything.
```

1.2 GPT-4 prompting for interfacing with DD+AR

System message

```
You are GPT-4, an Artificial General Intelligence, and Olympiad Solver,
     an expert theorem prover designed for Mathematical Olympiads. You
    are participating in the International Mathematical Olympiad (IMO)
    and you will be asked to solve Geometry problems. Your task is to
    provide auxiliary constructions in a formal language,such that a
    symbolic engine can parse your output and perform symbolic
    deductions to attempt to solve the IMO problem. You will be
    provided with the output of the symbolic engine to start
    constructing the first auxiliary point. If the symbolic engine
    failed to solve the problem, you will be asked to construct the
    next auxiliary point. This process will repeat multiple times until
     you can solve the problem.
```

```
Following is the grammar to construct a new point:
<point name> : <first predicate> , <second predicate>

Where the new point's name is a single character and each predicate can be one of six cases:

perp A B C D : AB is perpendicular to CD
para A B C D : AB is parallel to CD
cong A B C D : segment AB has the same length to CD
coll A B C : A, B, and C are collinear
cyclic A B C D : A B C D are concyclic
eqangle A B C D E F : the angle ABC = the angle DEF

For example, to construct the midpoint M of segment BC, output this:

M : coll M B C , cong M B M C

Because any M that satisfies (1) being collinear to B and C, and (2) being equidistance to B and C, uniquely defines the midpoint of BC.

To construct the perpendicular foot D from vertex A of triangle ABC, output this:

D : perp A D B C , coll D B C

To construct the touch point T of the tangent line from A to a circle with center O and radius equal to BC, output this:

T : perp A T O T , cong O T B C

Once the auxiliary construction is parsed, the symbolic deduction engine will use it together with the problem to deduce new predicates. Each predicate is either one of the above six types.

Some conventions in problem statements:

XYZ is considered angle XYZ (angle between lines XY and YZ), unless explicitly stated to be a triangle.
(XY, ZT) is considered the angle between lines XY and ZT.
(X, Y) is the circle centered at X and pass through Y.
XY is the line passing through points X and Y, unless explicitly stated to be a segment, or explicitly stated to be equal some other segments such as "XY = AB".
```

Reflect and revise message

```
The symbolic engine failed to solve the problem with this auxiliary point. Here is its new output:

<list of DD+AR newly deduced statements>

Let's continue to construct another different auxiliary point. Maybe its combination with the previously constructed points will succeed.

Please think deeply, step by step, on how and why the next auxiliary construction can help, and at the end of your response, output a
```

```
single line of text, with the correct grammar described above, to
construct one auxiliary point with name <an unused point name>,
that will help the symbolic engine solve the problem.
```

2 G0 solutions to classical geometry in IMO 2000-2022

2.1 IMO 2000 P1

Original:

Two circles G1 and G2 intersect at two points M and N. Let AB be the line tangent to these circles at A and B, respectively, so that M lies closer to AB than N. Let CD be the line parallel to AB and passing through the point M, with C on G1 and D on G2. Lines AC and BD meet at E; lines AN and CD meet at P; lines BN and CD meet at Q. Show that EP = EQ.

Translated:

Let A and B be any two distinct points. Define point G_1 such that AB is perpendicular to AG_1. Define point G_2 such that AB is perpendicular to BG_2. Define point M as the intersection of circles (G_1, A) and (G_2, B). Define point N as the intersection of circles (G_1, A) and (G_2, B). Define point C on circle (G_1, A) such that AB is parallel to CM. Define point D on circle (G_2, B) such that AB is parallel to DM. Define point E as the intersection of lines AC and BD. Define point P as the intersection of lines AN and CD. Define point Q as the intersection of lines BN and CD. Prove that $EP = EQ$

Proof:

Step 1. $BG_2 = DG_2$ and $BG_2 = G_2M \Rightarrow G_2$ is the circumcenter of BDM.

Step 2. G_2 is the circumcenter of BDM and AB is perpendicular to BG_2 $\Rightarrow \measuredangle ABD = \measuredangle BMD$.

Step 3. $AG_1 = CG_1$ and $AG_1 = G_1M \Rightarrow G_1$ is the circumcenter of ACM.

Step 4. G_1 is the circumcenter of ACM and AB is perpendicular to AG_1 $\Rightarrow \measuredangle BAM = \measuredangle ACM$.

Step 5. B, D, E are collinear, $\measuredangle ABD = \measuredangle BMD$ and AB is parallel to DM $\Rightarrow BA$ is the bisector of $\measuredangle MBE$.

Step 6. A, C, E are collinear, $\measuredangle BAM = \measuredangle ACM$ and AB is parallel to CM $\Rightarrow AB$ is the bisector of $\measuredangle MAE$.

Step 7. AB is the bisector of $\measuredangle MAE$ and BA is the bisector of $\measuredangle MBE \Rightarrow$ $AE = AM$ and $BE = BM$.

Step 8. $AG_1 = G_1M$ and $AG_1 = G_1N \Rightarrow G_1$ is the circumcenter of AMN.

Step 9. G_1 is the circumcenter of AMN and AB is perpendicular to AG_1 $\Rightarrow \measuredangle BAM = \measuredangle ANM$ and $\measuredangle BAN = \measuredangle AMN$.

Step 10. AB is parallel to CM and AB is parallel to $DM \Rightarrow CM$ is parallel to DM.

Step 11. CM is parallel to $DM \Rightarrow C, D, M$ are collinear.

Step 12. A, N, P are collinear, C, D, M are collinear, C, D, P are collinear, $\measuredangle BAN = \measuredangle AMN$ and AB is parallel to $DM \Rightarrow \measuredangle AMN = \measuredangle MPA$.

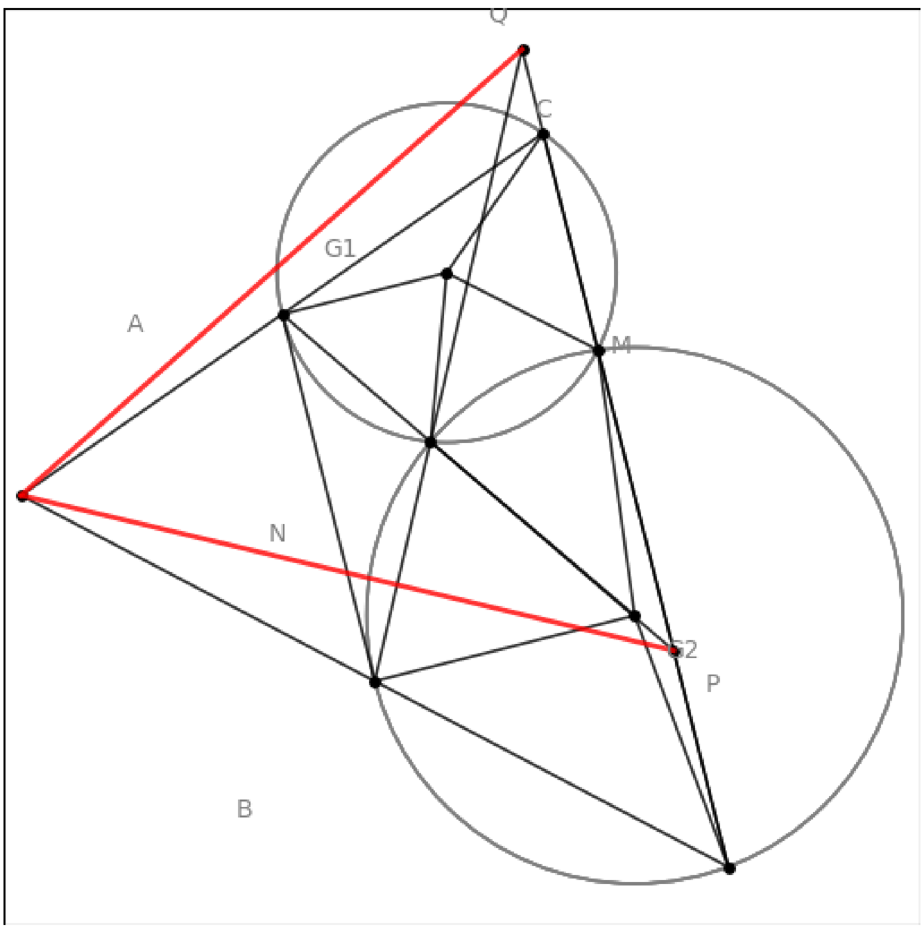

Figure 1: imo 2000 p1

Step 13. C, D, M are collinear, C, D, P are collinear, $\measuredangle BAM = \measuredangle ANM$ and AB is parallel to $DM \Rightarrow \measuredangle AMP = \measuredangle MNA$.

Step 14. $\measuredangle AMN = \measuredangle MPA$ and $\measuredangle AMP = \measuredangle MNA \Rightarrow \frac{AM}{AP} = \frac{AN}{AM}$.

Step 15. $AE = AM$ and $\frac{AM}{AP} = \frac{AN}{AM} \Rightarrow \frac{AE}{AP} = \frac{AN}{AE}$.

Step 16. A, N, P are collinear $\Rightarrow \measuredangle EAN = \measuredangle EAP$.

Step 17. $\measuredangle EAN = \measuredangle EAP$ and $\frac{AE}{AP} = \frac{AN}{AE} \Rightarrow \measuredangle AEN = \measuredangle EPA$.

Step 18. $AG_1 = CG_1$, $AG_1 = G_1M$ and $AG_1 = G_1N \Rightarrow A, C, M, N$ are cyclic.

Step 19. A, C, M, N are cyclic $\Rightarrow \measuredangle CAN = \measuredangle CMN$.

Step 20. $BG_2 = DG_2$, $BG_2 = G_2M$ and $BG_2 = G_2N \Rightarrow B, D, M, N$ are cyclic.

Step 21. B, D, M, N are cyclic $\Rightarrow \measuredangle DBN = \measuredangle DMN$.

Step 22. A, C, E are collinear, B, D, E are collinear, $\measuredangle CAN = \measuredangle CMN$,

$\measuredangle DBN = \measuredangle DMN$, AB is parallel to CM and AB is parallel to $DM \Rightarrow \measuredangle EAN = \measuredangle EBN$.

Step 23. $\measuredangle EAN = \measuredangle EBN \Rightarrow A, B, E, N$ are cyclic.

Step 24. A, B, E, N are cyclic $\Rightarrow \measuredangle BAE = \measuredangle BNE$ and $\measuredangle BAN = \measuredangle BEN$.

Step 25. $BG_2 = DG_2$ and $BG_2 = G_2N \Rightarrow G_2$ is the circumcenter of BDN.

Step 26. G_2 is the circumcenter of BDN and AB is perpendicular to BG_2 $\Rightarrow \measuredangle ABD = \measuredangle BND$.

Step 27. C, D, M are collinear, C, D, Q are collinear, $\measuredangle ABD = \measuredangle BND$ and AB is parallel to $DM \Rightarrow \measuredangle BDQ = \measuredangle DNB$.

Step 28. B, N, Q are collinear $\Rightarrow \measuredangle DBN = \measuredangle DBQ$.

Step 29. $\measuredangle DBN = \measuredangle DBQ$ and $\measuredangle BDQ = \measuredangle DNB \Rightarrow \frac{BD}{BQ} = \frac{BN}{BD}$.

Step 30. $BG_2 = G_2M$ and $BG_2 = G_2N \Rightarrow G_2$ is the circumcenter of BMN.

Step 31. G_2 is the circumcenter of BMN and AB is perpendicular to BG_2 $\Rightarrow \measuredangle ABM = \measuredangle BNM$.

Step 32. $\measuredangle ABM = \measuredangle BNM$ and AB is parallel to $DM \Rightarrow \measuredangle BNM = \measuredangle DMB$.

Step 33. B, D, M, N are cyclic and $\measuredangle BNM = \measuredangle DMB \Rightarrow BD = BM$.

Step 34. $BD = BM$, $BE = BM$ and $\frac{BD}{BQ} = \frac{BN}{BD} \Rightarrow \frac{BE}{BQ} = \frac{BN}{BE}$.

Step 35. B, N, Q are collinear $\Rightarrow \measuredangle EBN = \measuredangle EBQ$.

Step 36. $\measuredangle EBN = \measuredangle EBQ$ and $\frac{BE}{BQ} = \frac{BN}{BE} \Rightarrow \measuredangle BEN = \measuredangle EQB$.

Step 37. A, N, P are collinear, $\measuredangle BAE = \measuredangle BNE$, $\measuredangle AEN = \measuredangle EPA$ and AB is parallel to $DM \Rightarrow \measuredangle(BN, DM) = \measuredangle NPE$.

Step 38. A, N, P are collinear, B, N, Q are collinear, $\measuredangle BAN = \measuredangle BEN$, $\measuredangle BEN = \measuredangle EQB$ and AB is parallel to $DM \Rightarrow \measuredangle(BN, EQ) = \measuredangle(NP, DM)$.

Step 39. $\measuredangle(BN, DM) = \measuredangle NPE$ and $\measuredangle(BN, EQ) = \measuredangle(NP, DM) \Rightarrow \measuredangle(DM, EQ) = \measuredangle(EP, DM)$.

Step 40. C, D, M are collinear, C, D, P are collinear, C, D, Q are collinear and $\measuredangle(DM, EQ) = \measuredangle(EP, DM) \Rightarrow \measuredangle EPQ = \measuredangle PQE$.

Step 41. $\measuredangle EPQ = \measuredangle PQE \Rightarrow EP = EQ$

■

2.2 IMO 2000 P6

Original:

Let AH1, BH2, and CH3 be the altitudes of a triangle ABC. The incircle W of triangle ABC touches the sides BC, CA and AB at T1, T2 and T3, respectively. Consider the symmetric images of the lines H1H2, H2H3, and H3H1 with respect to the lines T1T2, T2T3, and T3T1. Prove that these images form a triangle whose vertices lie on W.

Translated:

Let ABC be a triangle. Define point I such that AI is the bisector of $\measuredangle BAC$ and CI is the bisector of $\measuredangle ACB$. Define point T_1 as the foot of I on line BC. Define point T_2 as the foot of I on line AC. Define point T_3 as the foot of I on line AB. Define point H_1 as the foot of A on line BC. Define point H_2 as the foot of B on line AC. Define point H_3 as the foot of C on line AB. Define point X_1 as the intersection of circles (T_1, H_1) and (T_2, H_1). Define point X_2 as the

intersection of circles (T_1, H_2) and (T_2, H_2). Define point Y_2 as the intersection of circles (T_2, H_2) and (T_3, H_2). Define point Y_3 as the intersection of circles (T_2, H_3) and (T_3, H_3). Define point Z as the intersection of lines X_1X_2 and Y_2Y_3. Prove that $T_1I = IZ$

Proof:

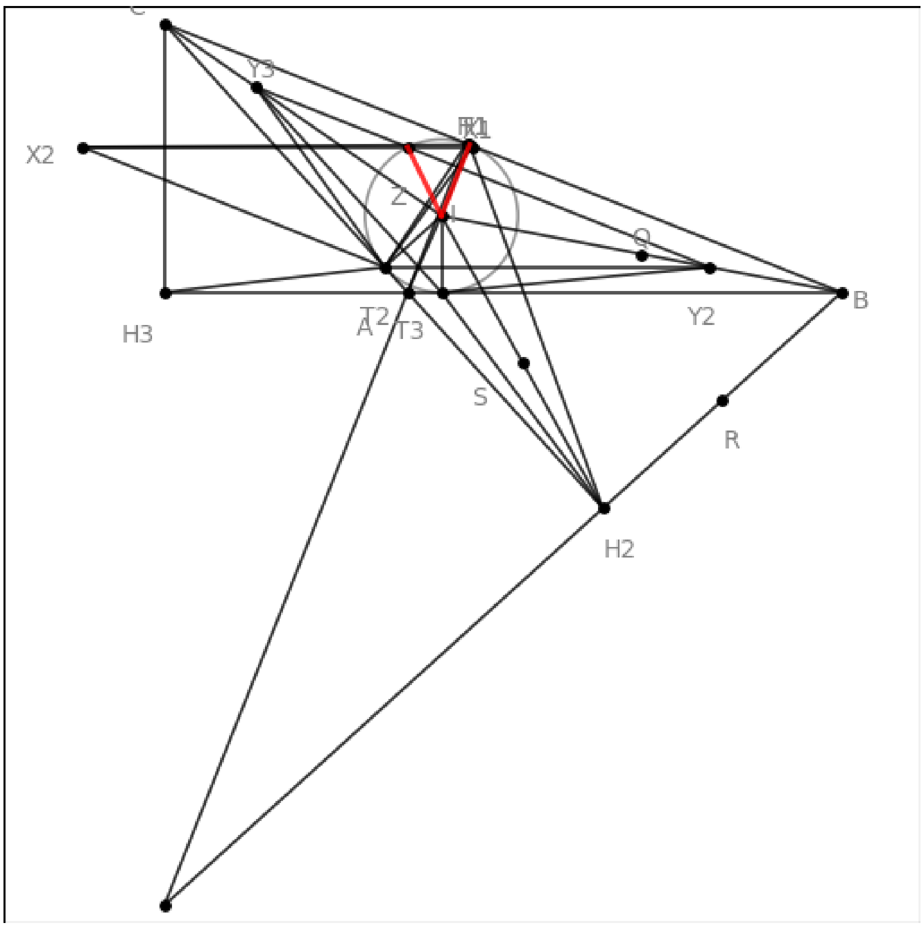

Figure 2: imo 2000 p6

Construct point Q as the midpoint of BI.

Construct point S as the midpoint of IH_2.

Step 1. $T_2H_2 = T_2Y_2 \Rightarrow \measuredangle T_2H_2Y_2 = \measuredangle H_2Y_2T_2$.

Step 2. $T_2H_3 = T_2Y_3$ and $T_3H_3 = T_3Y_3 \Rightarrow T_2T_3$ is perpendicular to H_3Y_3.

Step 3. $T_2H_3 = T_2Y_3$ and $T_3H_3 = T_3Y_3 \Rightarrow \measuredangle T_2H_3T_3 = \measuredangle T_3Y_3T_2$.

Step 4. $T_2H_2 = T_2Y_2$ and $T_3H_2 = T_3Y_2 \Rightarrow T_2T_3$ is perpendicular to H_2Y_2.

Step 5. A, C, T_2 are collinear, A, C, H_2 are collinear, $\measuredangle T_2H_2Y_2 = \measuredangle H_2Y_2T_2$, T_2T_3 is perpendicular to H_2Y_2 and T_2T_3 is perpendicular to $H_3Y_3 \Rightarrow \measuredangle(AC, H_3Y_3) =$

$\measuredangle(H_3Y_3, T_2Y_2)$.

Step 6. AC is perpendicular to T_2I and T_2T_3 is perpendicular to $H_3Y_3 \Rightarrow \measuredangle(AC, T_2I) = \measuredangle(H_3Y_3, T_2T_3)$.

Step 7. $\measuredangle(AC, T_2I) = \measuredangle(H_3Y_3, T_2T_3)$ and $\measuredangle(AC, H_3Y_3) = \measuredangle(H_3Y_3, T_2Y_2) \Rightarrow \measuredangle T_3T_2I = \measuredangle(T_2Y_2, H_3Y_3)$.

Step 8. B, I, Q are collinear and $BQ = IQ \Rightarrow Q$ is the midpoint of BI.

Step 9. I, H_2, S are collinear and $IS = H_2S \Rightarrow S$ is the midpoint of IH_2.

Step 10. Q is the midpoint of BI and S is the midpoint of $IH_2 \Rightarrow BH_2$ is parallel to QS.

Step 11. A, B, T_3 are collinear, A, C, T_2 are collinear, AB is perpendicular to T_3I and AC is perpendicular to $T_2I \Rightarrow \measuredangle AT_2I = \measuredangle IT_3A$.

Step 12. A, B, T_3 are collinear, A, C, T_2 are collinear and AI is the bisector of $\measuredangle BAC \Rightarrow AI$ is the bisector of $\measuredangle T_2AT_3$.

Step 13. AI is the bisector of $\measuredangle T_2AT_3$ and $\measuredangle AT_2I = \measuredangle IT_3A \Rightarrow T_2I = T_3I$ and $AT_2 = AT_3$.

Step 14. $T_2I = T_3I \Rightarrow \measuredangle T_2T_3I = \measuredangle IT_2T_3$.

Step 15. A, B, T_3 are collinear and AB is perpendicular to $T_3I \Rightarrow AT_3$ is perpendicular to T_3I.

Step 16. AT_3 is perpendicular to T_3I and T_2T_3 is perpendicular to $H_3Y_3 \Rightarrow \measuredangle(AT_3, H_3Y_3) = \measuredangle IT_3T_2$.

Step 17. $\measuredangle(AT_3, H_3Y_3) = \measuredangle IT_3T_2$, $\measuredangle T_3T_2I = \measuredangle(T_2Y_2, H_3Y_3)$ and $\measuredangle T_2T_3I = \measuredangle IT_2T_3 \Rightarrow \measuredangle(AT_3, H_3Y_3) = \measuredangle(T_2Y_2, H_3Y_3)$.

Step 18. $\measuredangle(AT_3, H_3Y_3) = \measuredangle(T_2Y_2, H_3Y_3) \Rightarrow AT_3$ is parallel to T_2Y_2.

Step 19. $AT_2 = AT_3$ and $T_2I = T_3I \Rightarrow AI$ is perpendicular to T_2T_3.

Step 20. $T_3H_3 = T_3Y_3 \Rightarrow \measuredangle T_3H_3Y_3 = \measuredangle H_3Y_3T_3$.

Step 21. A, B, T_3 are collinear, A, B, H_3 are collinear, AI is the bisector of $\measuredangle BAC$, $\measuredangle T_3H_3Y_3 = \measuredangle H_3Y_3T_3$, AI is perpendicular to T_2T_3 and T_2T_3 is perpendicular to $H_3Y_3 \Rightarrow \measuredangle(AC, H_3Y_3) = \measuredangle T_3Y_3H_3$.

Step 22. $\measuredangle(AC, H_3Y_3) = \measuredangle T_3Y_3H_3 \Rightarrow AC$ is parallel to T_3Y_3.

Step 23. A, B, T_3 are collinear, A, B, H_3 are collinear, A, C, T_2 are collinear, A, C, H_2 are collinear, $\measuredangle T_2H_3T_3 = \measuredangle T_3Y_3T_2$, AC is parallel to T_3Y_3 and AT_3 is parallel to $T_2Y_2 \Rightarrow \measuredangle H_2T_2H_3 = \measuredangle Y_3T_2Y_2$.

Step 24. $T_2H_2 = T_2Y_2$, $T_2H_3 = T_2Y_3$ and $\measuredangle H_2T_2H_3 = \measuredangle Y_3T_2Y_2 \Rightarrow \measuredangle T_2Y_2Y_3 = \measuredangle H_3H_2T_2$.

Step 25. A, C, T_2 are collinear, B, C, T_1 are collinear, AC is perpendicular to T_2I and BC is perpendicular to $T_1I \Rightarrow \measuredangle CT_1I = \measuredangle IT_2C$.

Step 26. A, C, T_2 are collinear, B, C, T_1 are collinear and CI is the bisector of $\measuredangle ACB \Rightarrow CI$ is the bisector of $\measuredangle T_1CT_2$.

Step 27. CI is the bisector of $\measuredangle T_1CT_2$ and $\measuredangle CT_1I = \measuredangle IT_2C \Rightarrow T_1I = T_2I$ and $CT_1 = CT_2$.

Step 28. $T_1I = T_2I \Rightarrow \measuredangle T_1T_2I = \measuredangle IT_1T_2$.

Step 29. $T_1H_1 = T_1X_1 \Rightarrow \measuredangle T_1H_1X_1 = \measuredangle H_1X_1T_1$.

Step 30. $T_1H_2 = T_1X_2$ and $T_2H_2 = T_2X_2 \Rightarrow T_1T_2$ is perpendicular to H_2X_2.

Step 31. $T_1H_2 = T_1X_2$ and $T_2H_2 = T_2X_2 \Rightarrow \measuredangle T_1H_2T_2 = \measuredangle T_2X_2T_1$.

Step 32. $T_1H_1 = T_1X_1$ and $T_2H_1 = T_2X_1 \Rightarrow T_1T_2$ is perpendicular to H_1X_1.

Step 33. B, C, T_1 are collinear, B, C, H_1 are collinear, $\measuredangle T_1H_1X_1 = \measuredangle H_1X_1T_1$, T_1T_2 is perpendicular to H_1X_1 and T_1T_2 is perpendicular to $H_2X_2 \Rightarrow \measuredangle(CH_1, H_2X_2) = \measuredangle(H_2X_2, T_1X_1)$.

Step 34. B, C, H_1 are collinear, BC is perpendicular to T_1I and T_1T_2 is perpendicular to $H_2X_2 \Rightarrow \measuredangle(CH_1, T_1I) = \measuredangle(H_2X_2, T_1T_2)$.

Step 35. $\measuredangle(CH_1, T_1I) = \measuredangle(H_2X_2, T_1T_2)$ and $\measuredangle(CH_1, H_2X_2) = \measuredangle(H_2X_2, T_1X_1)$ $\Rightarrow \measuredangle T_2T_1I = \measuredangle(T_1X_1, H_2X_2)$.

Step 36. AC is perpendicular to T_2I and T_1T_2 is perpendicular to $H_2X_2 \Rightarrow \measuredangle(AC, H_2X_2) = \measuredangle IT_2T_1$.

Step 37. $\measuredangle(AC, H_2X_2) = \measuredangle IT_2T_1$, $\measuredangle T_2T_1I = \measuredangle(T_1X_1, H_2X_2)$ and $\measuredangle T_1T_2I = \measuredangle IT_1T_2 \Rightarrow \measuredangle(AC, H_2X_2) = \measuredangle(T_1X_1, H_2X_2)$.

Step 38. $\measuredangle(AC, H_2X_2) = \measuredangle(T_1X_1, H_2X_2) \Rightarrow AC$ is parallel to T_1X_1.

Step 39. A, B, H_3 are collinear, A, C, H_2 are collinear, AB is perpendicular to CH_3 and AC is perpendicular to $BH_2 \Rightarrow \measuredangle BH_2C = \measuredangle BH_3C$.

Step 40. $\measuredangle BH_2C = \measuredangle BH_3C \Rightarrow B, C, H_2, H_3$ are cyclic.

Step 41. B, C, H_2, H_3 are cyclic $\Rightarrow \measuredangle CBH_3 = \measuredangle CH_2H_3$ and $\measuredangle BCH_3 = \measuredangle BH_2H_3$.

Step 42. $CT_1 = CT_2$ and $T_1I = T_2I \Rightarrow CI$ is perpendicular to T_1T_2.

Step 43. $T_2H_2 = T_2X_2 \Rightarrow \measuredangle T_2H_2X_2 = \measuredangle H_2X_2T_2$.

Step 44. A, C, T_2 are collinear, A, C, H_2 are collinear, B, C, H_1 are collinear, CI is the bisector of $\measuredangle ACB$, $\measuredangle T_2H_2X_2 = \measuredangle H_2X_2T_2$, CI is perpendicular to T_1T_2 and T_1T_2 is perpendicular to $H_2X_2 \Rightarrow \measuredangle(CH_1, H_2X_2) = \measuredangle T_2X_2H_2$.

Step 45. $\measuredangle(CH_1, H_2X_2) = \measuredangle T_2X_2H_2 \Rightarrow CH_1$ is parallel to T_2X_2.

Step 46. A, B, T_3 are collinear, A, B, H_3 are collinear, A, C, T_2 are collinear, A, C, H_2 are collinear, B, C, H_1 are collinear, Y_2, Y_3, Z are collinear, $\measuredangle CBH_3 = \measuredangle CH_2H_3$, $\measuredangle T_2Y_2Y_3 = \measuredangle H_3H_2T_2$, AT_3 is parallel to T_2Y_2 and CH_1 is parallel to $T_2X_2 \Rightarrow \measuredangle(AC, T_2X_2) = \measuredangle(AC, Y_2Z)$.

Step 47. $\measuredangle(AC, T_2X_2) = \measuredangle(AC, Y_2Z) \Rightarrow T_2X_2$ is parallel to Y_2Z.

Step 48. A, C, T_2 are collinear, A, C, H_2 are collinear, B, C, T_1 are collinear, B, C, H_1 are collinear, $\measuredangle T_1H_2T_2 = \measuredangle T_2X_2T_1$, AC is parallel to T_1X_1 and CH_1 is parallel to $T_2X_2 \Rightarrow \measuredangle H_1T_1H_2 = \measuredangle X_2T_1X_1$.

Step 49. $T_1H_1 = T_1X_1$, $T_1H_2 = T_1X_2$ and $\measuredangle H_1T_1H_2 = \measuredangle X_2T_1X_1 \Rightarrow \measuredangle T_1X_1X_2 = \measuredangle H_2H_1T_1$.

Step 50. A, C, H_2 are collinear, B, C, H_1 are collinear, AC is perpendicular to BH_2 and AH_1 is perpendicular to $BC \Rightarrow \measuredangle AH_1B = \measuredangle AH_2B$.

Step 51. $\measuredangle AH_1B = \measuredangle AH_2B \Rightarrow A, B, H_1, H_2$ are cyclic.

Step 52. A, B, H_1, H_2 are cyclic $\Rightarrow \measuredangle ABH_1 = \measuredangle AH_2H_1$.

Step 53. A, B, T_3 are collinear, A, C, H_2 are collinear, B, C, T_1 are collinear, B, C, H_1 are collinear, X_1, X_2, Z are collinear, $\measuredangle ABH_1 = \measuredangle AH_2H_1$, $\measuredangle T_1X_1X_2 = \measuredangle H_2H_1T_1$, AC is parallel to T_1X_1 and AT_3 is parallel to $T_2Y_2 \Rightarrow \measuredangle X_2T_2Y_2 = \measuredangle(T_2X_2, X_1Z)$.

Step 54. $\measuredangle X_2T_2Y_2 = \measuredangle(T_2X_2, X_1Z) \Rightarrow T_2Y_2$ is parallel to X_1Z.

Step 55. $T_2H_2 = T_2X_2$ and $T_2H_2 = T_2Y_2 \Rightarrow T_2X_2 = T_2Y_2$.

Step 56. $T_2X_2 = T_2Y_2 \Rightarrow \measuredangle T_2X_2Y_2 = \measuredangle X_2Y_2T_2$.

Step 57. X_1, X_2, Z are collinear, T_2X_2 is parallel to Y_2Z and T_2Y_2 is parallel to $X_1Z \Rightarrow \measuredangle X_2T_2Y_2 = \measuredangle Y_2ZX_2$.

Step 58. $\measuredangle T_2X_2Y_2 = \measuredangle X_2Y_2T_2$ and T_2X_2 is parallel to $Y_2Z \Rightarrow Y_2X_2$ is the bisector of $\measuredangle T_2Y_2Z$.

Step 59. $\measuredangle X_2T_2Y_2 = \measuredangle Y_2ZX_2$ and Y_2X_2 is the bisector of $\measuredangle T_2Y_2Z \Rightarrow T_2Y_2 = Y_2Z$ and $T_2X_2 = X_2Z$.

Step 60. $T_2X_2 = X_2Z$ and $T_2Y_2 = Y_2Z \Rightarrow T_2Z$ is perpendicular to X_2Y_2.

Step 61. $T_2H_2 = T_2X_2$ and $T_2H_2 = T_2Y_2 \Rightarrow T_2$ is the circumcenter of $H_2X_2Y_2$.

Step 62. A, C, T_2 are collinear, A, C, H_2 are collinear and AC is perpendicular to $BH_2 \Rightarrow BH_2$ is perpendicular to T_2H_2.

Step 63. T_2 is the circumcenter of $H_2X_2Y_2$ and BH_2 is perpendicular to $T_2H_2 \Rightarrow \measuredangle BH_2Y_2 = \measuredangle H_2X_2Y_2$.

Step 64. $\measuredangle BH_2Y_2 = \measuredangle H_2X_2Y_2$, AC is perpendicular to BH_2, AC is perpendicular to T_2I, T_2T_3 is perpendicular to H_2Y_2 and T_2T_3 is perpendicular to $H_3Y_3 \Rightarrow \measuredangle(T_2I, H_3Y_3) = \measuredangle H_2X_2Y_2$.

Step 65. AC is perpendicular to T_2I and T_1T_2 is perpendicular to $H_2X_2 \Rightarrow \measuredangle(AC, T_2I) = \measuredangle(T_1T_2, H_2X_2)$.

Step 66. $\measuredangle(AC, T_2I) = \measuredangle(T_1T_2, H_2X_2)$ and $\measuredangle(T_2I, H_3Y_3) = \measuredangle H_2X_2Y_2 \Rightarrow \measuredangle(AC, T_1T_2) = \measuredangle(H_3Y_3, X_2Y_2)$.

Step 67. $T_1I = T_2I$ and $T_2I = T_3I \Rightarrow I$ is the circumcenter of $T_1T_2T_3$.

Step 68. A, C, T_2 are collinear and AC is perpendicular to $T_2I \Rightarrow AT_2$ is perpendicular to T_2I.

Step 69. I is the circumcenter of $T_1T_2T_3$ and AT_2 is perpendicular to $T_2I \Rightarrow \measuredangle AT_2T_3 = \measuredangle T_2T_1T_3$.

Step 70. A, C, T_2 are collinear, $\measuredangle(AC, T_1T_2) = \measuredangle(H_3Y_3, X_2Y_2)$ and $\measuredangle AT_2T_3 = \measuredangle T_2T_1T_3 \Rightarrow \measuredangle T_1T_3T_2 = \measuredangle(X_2Y_2, H_3Y_3)$.

Step 71. T_2T_3 is perpendicular to $H_3Y_3 \Rightarrow \measuredangle(T_2T_3, H_3Y_3) = \measuredangle(H_3Y_3, T_2T_3)$.

Step 72. $\measuredangle(T_2T_3, H_3Y_3) = \measuredangle(H_3Y_3, T_2T_3)$ and $\measuredangle T_1T_3T_2 = \measuredangle(X_2Y_2, H_3Y_3) \Rightarrow \measuredangle(T_1T_3, X_2Y_2) = \measuredangle(H_3Y_3, T_2T_3)$.

Step 73. A, B, T_3 are collinear and AB is perpendicular to $T_3I \Rightarrow BT_3$ is perpendicular to T_3I.

Step 74. Q is the midpoint of BI and BT_3 is perpendicular to $T_3I \Rightarrow T_3Q = IQ$.

Step 75. B, C, T_1 are collinear and BC is perpendicular to $T_1I \Rightarrow BT_1$ is perpendicular to T_1I.

Step 76. Q is the midpoint of BI and BT_1 is perpendicular to $T_1I \Rightarrow T_1Q = IQ$.

Step 77. $T_1I = T_2I$ and $T_2I = T_3I \Rightarrow T_1I = T_3I$.

Step 78. $T_1Q = IQ$ and $T_3Q = IQ \Rightarrow T_1Q = T_3Q$.

Step 79. $T_1I = T_3I$ and $T_1Q = T_3Q \Rightarrow T_1T_3$ is perpendicular to IQ.

Step 80. AC is perpendicular to T_2I and T_1T_3 is perpendicular to $IQ \Rightarrow \measuredangle(AC, T_1T_3) = \measuredangle T_2IQ$.

Step 81. B, C, H_1 are collinear and BC is perpendicular to $T_1I \Rightarrow CH_1$ is perpendicular to T_1I.

Step 82. AC is perpendicular to T_2I and CH_1 is perpendicular to $T_1I \Rightarrow \measuredangle(AC, T_1I) = \measuredangle(T_2I, CH_1)$.

Step 83. $T_1I = T_3I$, $T_1Q = IQ$ and $T_3Q = IQ \Rightarrow \measuredangle IT_1Q = \measuredangle T_3IQ$.

Step 84. B, C, H_1 are collinear, $\measuredangle(AC, T_1I) = \measuredangle(T_2I, CH_1)$, $\measuredangle BCH_3 = \measuredangle BH_2H_3$, AB is perpendicular to CH_3, AB is perpendicular to T_3I, AC is perpendicular to BH_2 and AC is perpendicular to $T_2I \Rightarrow \measuredangle(AC, T_1I) = \measuredangle(H_2H_3, T_3I)$.

Step 85. $\measuredangle(AC, T_1I) = \measuredangle(H_2H_3, T_3I)$ and $\measuredangle IT_1Q = \measuredangle T_3IQ \Rightarrow \measuredangle(AC, T_1Q) = \measuredangle(H_2H_3, IQ)$.

Step 86. $T_1Q = IQ$ and $T_2Y_2 = Y_2Z \Rightarrow \frac{QT_1}{QI} = \frac{Y_2T_2}{Y_2Z}$.

Step 87. A, C, T_2 are collinear, A, C, H_2 are collinear, Y_2, Y_3, Z are collinear, $\measuredangle(AC, T_1Q) = \measuredangle(H_2H_3, IQ)$ and $\measuredangle T_2Y_2Y_3 = \measuredangle H_3H_2T_2 \Rightarrow \measuredangle T_1QI = \measuredangle ZY_2T_2$.

Step 88. $\measuredangle T_1QI = \measuredangle ZY_2T_2$ and $\frac{QT_1}{QI} = \frac{Y_2T_2}{Y_2Z} \Rightarrow \frac{T_1I}{T_1Q} = \frac{T_2Z}{T_2Y_2}$.

Step 89. A, C, T_2 are collinear, A, C, H_2 are collinear, $\measuredangle(AC, T_1T_3) = \measuredangle T_2IQ$, $\measuredangle(T_1T_3, X_2Y_2) = \measuredangle(H_3Y_3, T_2T_3)$, T_2T_3 is perpendicular to H_3Y_3 and T_2Z is perpendicular to $X_2Y_2 \Rightarrow \measuredangle T_2IQ = \measuredangle H_2T_2Z$.

Step 90. $T_1I = T_2I$, $T_1Q = IQ$, $T_2H_2 = T_2X_2$, $T_2X_2 = T_2Y_2$ and $\frac{T_1I}{T_1Q} = \frac{T_2Z}{T_2Y_2} \Rightarrow \frac{IT_2}{IQ} = \frac{T_2Z}{T_2H_2}$.

Step 91. $\measuredangle T_2IQ = \measuredangle H_2T_2Z$ and $\frac{IT_2}{IQ} = \frac{T_2Z}{T_2H_2} \Rightarrow \measuredangle T_2H_2Z = \measuredangle T_2QI$ and $\frac{T_2I}{T_2Z} = \frac{T_2Q}{H_2Z}$.

Step 92. A, C, T_2 are collinear, A, C, H_2 are collinear and AC is perpendicular to $T_2I \Rightarrow T_2I$ is perpendicular to T_2H_2.

Step 93. S is the midpoint of IH_2 and T_2I is perpendicular to $T_2H_2 \Rightarrow T_2S = IS$ and $T_2S = H_2S$.

Step 94. $T_2S = IS \Rightarrow \measuredangle T_2IS = \measuredangle ST_2I$.

Step 95. I, H_2, S are collinear, $\measuredangle T_2IS = \measuredangle ST_2I$, BH_2 is parallel to QS, AC is perpendicular to BH_2 and AC is perpendicular to $T_2I \Rightarrow SQ$ is the bisector of $\measuredangle T_2SH_2$.

Step 96. $T_2S = H_2S$ and SQ is the bisector of $\measuredangle T_2SH_2 \Rightarrow T_2Q = H_2Q$ and QS is the bisector of $\measuredangle T_2QH_2$.

Step 97. $T_2Q = H_2Q \Rightarrow \measuredangle T_2H_2Q = \measuredangle QT_2H_2$.

Step 98. AT_3 is perpendicular to T_3I and CH_1 is perpendicular to $T_1I \Rightarrow \measuredangle(AT_3, CH_1) = \measuredangle T_3IT_1$.

Step 99. A, C, T_2 are collinear, A, C, H_2 are collinear, $\measuredangle T_2H_2Z = \measuredangle T_2QI$ and $\measuredangle T_2H_2Q = \measuredangle QT_2H_2 \Rightarrow \measuredangle(AC, H_2Q) = \measuredangle(IQ, H_2Z)$.

Step 100. A, B, T_3 are collinear, A, C, H_2 are collinear, B, C, H_1 are collinear, $\measuredangle(AT_3, CH_1) = \measuredangle T_3IT_1$, $\measuredangle ABH_1 = \measuredangle AH_2H_1$ and $\measuredangle IT_1Q = \measuredangle T_3IQ \Rightarrow \measuredangle(AC, H_1H_2) = \measuredangle IQT_1$.

Step 101. $\measuredangle(AC, H_1H_2) = \measuredangle IQT_1$ and $\measuredangle(AC, H_2Q) = \measuredangle(IQ, H_2Z) \Rightarrow \measuredangle(T_1Q, H_1H_2) = \measuredangle ZH_2Q$.

Step 102. AC is perpendicular to T_2I and T_1T_3 is perpendicular to $IQ \Rightarrow \measuredangle(AC, T_2I) = \measuredangle(IQ, T_1T_3)$.

Step 103. $\measuredangle(AC, T_2I) = \measuredangle(IQ, T_1T_3)$ and $\measuredangle(AC, H_1H_2) = \measuredangle IQT_1 \Rightarrow \measuredangle(T_1T_3, T_2I) = \measuredangle(T_1Q, H_1H_2)$.

Step 104. $\measuredangle(T_1T_3, T_2I) = \measuredangle(T_1Q, H_1H_2)$, $\measuredangle(T_1T_3, X_2Y_2) = \measuredangle(H_3Y_3, T_2T_3)$, $\measuredangle(T_1Q, H_1H_2) = \measuredangle ZH_2Q$, T_2T_3 is perpendicular to H_3Y_3 and T_2Z is perpendicular to $X_2Y_2 \Rightarrow \measuredangle IT_2Z = \measuredangle QH_2Z$.

Step 105. $T_2Q = H_2Q$ and $\frac{T_2I}{T_2Z} = \frac{T_2Q}{H_2Z} \Rightarrow \frac{T_2I}{T_2Z} = \frac{H_2Q}{H_2Z}$.

Step 106. $\measuredangle IT_2Z = \measuredangle QH_2Z$ and $\frac{T_2I}{T_2Z} = \frac{H_2Q}{H_2Z} \Rightarrow \measuredangle(T_2I, H_2Q) = \measuredangle IZQ$ and $\frac{T_2I}{H_2Q} = \frac{ZI}{ZQ}$.

Step 107. $\measuredangle(T_2I, H_2Q) = \measuredangle IZQ$, QS is the bisector of $\measuredangle T_2QH_2$, BH_2 is parallel to QS, AC is perpendicular to BH_2 and AC is perpendicular to $T_2I \Rightarrow \measuredangle QT_2I = \measuredangle IZQ$.

Step 108. $T_2Q = H_2Q$ and $\frac{T_2I}{H_2Q} = \frac{ZI}{ZQ} \Rightarrow \frac{T_2I}{T_2Q} = \frac{ZI}{ZQ}$.

Step 109. $\measuredangle QT_2I = \measuredangle IZQ$ and $\frac{T_2I}{T_2Q} = \frac{ZI}{ZQ} \Rightarrow T_2I = IZ$.

Step 110. $T_1I = T_2I$ and $T_2I = IZ \Rightarrow T_1I = IZ$

■

2.3 IMO 2002 P2A

Original:

Let BC be a diameter of circle W with center O. Let A be a point of circle W such that $0^o < \measuredangle AOB < 120^o$. Let D be the midpoint of arc AB not containing C. Line l passes through O and is parallel to line AD. Line l intersects line AC at J. The perpendicular bisector of segment OA intersects circle W at E and F. Prove that EJ is the angle bisector of angle CEF.

Translated:

Let B and C be any two distinct points. Define point O as the midpoint of BC. Let A be any point on circle (O, B). Define point D as the circumcenter of triangle BAO. Define point E on circle (O, B) such that $AE = EO$, $\measuredangle EAO = \measuredangle AOE$ and $\measuredangle AEO = \measuredangle AEO$. Define point F on circle (O, B) such that $AF = FO$ and $\measuredangle FAO = \measuredangle AOF$. Define point J on line AC such that AD is parallel to JO. Prove that EJ is the bisector of $\measuredangle CEF$

Proof:

Step 1. $AO = BO$ and $BO = CO \Rightarrow O$ is the circumcenter of ABC.

Step 2. O is the circumcenter of ABC and B, C, O are collinear $\Rightarrow AB$ is perpendicular to AC.

Step 3. $AD = BD$ and $AO = BO \Rightarrow AB$ is perpendicular to DO.

Step 4. $AO = BO$ and $BO = DO \Rightarrow AO = DO$.

Step 5. $AO = DO \Rightarrow \measuredangle ADO = \measuredangle OAD$.

Step 6. A, C, J are collinear, $\measuredangle ADO = \measuredangle OAD$, AD is parallel to JO, AB is perpendicular to AC and AB is perpendicular to $DO \Rightarrow \measuredangle AJO = \measuredangle JOA$.

Step 7. $\measuredangle AJO = \measuredangle JOA \Rightarrow AJ = AO$.

Step 8. $\measuredangle EAO = \measuredangle AOE$ and $\measuredangle AEO = \measuredangle AEO \Rightarrow \frac{AO}{AE} = \frac{OA}{OE}$.

Step 9. $\measuredangle FAO = \measuredangle AOF \Rightarrow \frac{AO}{AF} = \frac{OA}{OF}$.

Step 10. $AJ = AO$, $AO = BO$, $BO = EO$, $BO = FO$, $\frac{AO}{AE} = \frac{OA}{OE}$ and $\frac{AO}{AF} = \frac{OA}{OF} \Rightarrow E, F, J, O$ are cyclic.

Step 11. E, F, J, O are cyclic $\Rightarrow \measuredangle FEJ = \measuredangle FOJ$.

Step 12. $AE = EO$, $BO = EO$ and $BO = FO \Rightarrow AE = FO$.

Step 13. $AF = FO$, $BO = EO$ and $BO = FO \Rightarrow AF = EO$.

Step 14. $AE = FO$ and $AF = EO \Rightarrow AE$ is parallel to FO.

Step 15. $AO = BO$, $BO = DO$, $BO = EO$ and $BO = FO \Rightarrow A, D, E, F$ are cyclic.

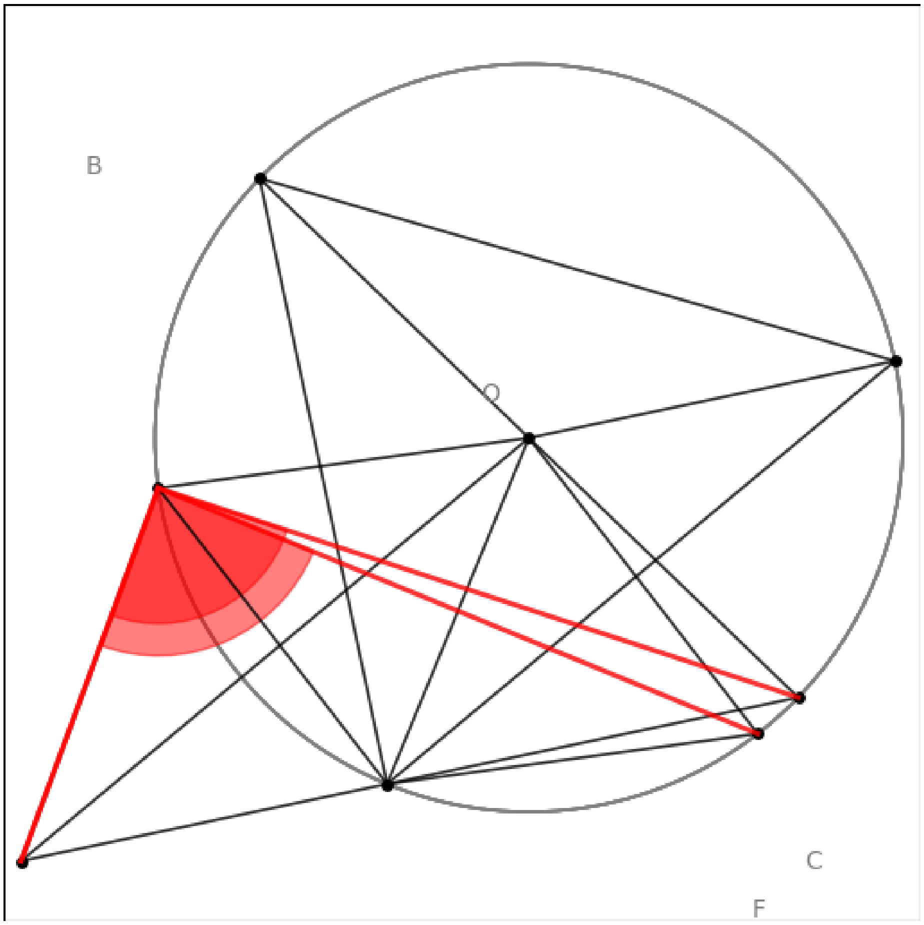

Figure 3: imo 2002 p2a

Step 16. A, D, E, F are cyclic $\Rightarrow \measuredangle ADF = \measuredangle AEF$.

Step 17. $AO = BO$, $BO = CO$, $BO = EO$ and $BO = FO \Rightarrow A, C, E, F$ are cyclic.

Step 18. A, C, E, F are cyclic $\Rightarrow \measuredangle ACE = \measuredangle AFE$.

Step 19. $AE = EO$, $AF = FO$, $BO = EO$ and $BO = FO \Rightarrow AE = AF$.

Step 20. $AE = AF \Rightarrow \measuredangle AEF = \measuredangle EFA$.

Step 21. $BO = DO$ and $BO = FO \Rightarrow DO = FO$.

Step 22. $DO = FO \Rightarrow \measuredangle DFO = \measuredangle ODF$.

Step 23. $\measuredangle ACE = \measuredangle AFE$, $\measuredangle ADF = \measuredangle AEF$ and $\measuredangle AEF = \measuredangle EFA \Rightarrow \measuredangle ADF = \measuredangle ECA$.

Step 24. $\measuredangle DFO = \measuredangle ODF$, AB is perpendicular to AC and AB is perpendicular to $DO \Rightarrow \measuredangle(AC, DF) = \measuredangle DFO$.

Step 25. $\measuredangle(AC, DF) = \measuredangle DFO$ and $\measuredangle ADF = \measuredangle ECA \Rightarrow \measuredangle(AD, FO) =$

$\measuredangle(CE, DF)$.

Step 26. A, C, J are collinear, AD is parallel to JO, AB is perpendicular to AC and AB is perpendicular to $DO \Rightarrow \measuredangle JAD = \measuredangle DOJ$.

Step 27. AD is parallel to $JO \Rightarrow \measuredangle ADJ = \measuredangle OJD$.

Step 28. $\measuredangle JAD = \measuredangle DOJ$ and $\measuredangle ADJ = \measuredangle OJD \Rightarrow AJ = DO$.

Step 29. A, C, J are collinear, AE is parallel to FO, AB is perpendicular to AC and AB is perpendicular to $DO \Rightarrow \measuredangle JAE = \measuredangle DOF$.

Step 30. $AE = FO$, $AJ = DO$ and $\measuredangle JAE = \measuredangle DOF \Rightarrow \measuredangle(AE, FO) = \measuredangle(EJ, DF)$.

Step 31. $\measuredangle(AD, FO) = \measuredangle(CE, DF)$, $\measuredangle(AE, FO) = \measuredangle(EJ, DF)$, $\measuredangle FEJ = \measuredangle FOJ$, AD is parallel to JO and AE is parallel to $FO \Rightarrow EJ$ is the bisector of $\measuredangle CEF$

■

2.4 IMO 2002 P2B

Original:

Let BC be a diameter of circle W with center O. Let A be a point of circle W such that $0^o < \measuredangle AOB < 120^o$. Let D be the midpoint of arc AB not containing C. Line l passes through O and is parallel to line AD. Line l intersects line AC at J. The perpendicular bisector of segment OA intersects circle W at E and F. Prove that CJ is the angle bisector of angle ECF.

Translated:

Let B and C be any two distinct points. Define point O such that $BO = CO$. Let A be any point on circle (O, B). Define point E as the circumcenter of triangle BAO. Define point F as the circumcenter of triangle BAO. Let J be any point on line AC. Prove that CJ is the bisector of $\measuredangle ECF$

Proof:

Step 1. $AO = BO$, $BO = CO$, $BO = EO$ and $BO = FO \Rightarrow A, C, E, F$ are cyclic.

Step 2. A, C, E, F are cyclic $\Rightarrow \measuredangle ACF = \measuredangle AEF$ and $\measuredangle ACE = \measuredangle AFE$.

Step 3. $AE = EO$, $AF = FO$, $BO = EO$ and $BO = FO \Rightarrow AE = AF$.

Step 4. $AE = AF \Rightarrow \measuredangle AEF = \measuredangle EFA$.

Step 5. A, C, J are collinear, $\measuredangle ACE = \measuredangle AFE$, $\measuredangle ACF = \measuredangle AEF$ and $\measuredangle AEF = \measuredangle EFA \Rightarrow CJ$ is the bisector of $\measuredangle ECF$

■

2.5 IMO 2003 P4

Original:

Let ABCD be a cyclic quadrilateral. Let P, Q and R be the feet of perpendiculars from D to lines BC, CA and AB respectively. Show that PQ = QR if the bisectors of angles ABC and ADC meet on segment AC.

Translated:

Let ABC be a triangle. Define point O as the circumcenter of triangle CBA. Define point B_1 on circle (O, A) such that $\measuredangle B_1AC = \measuredangle ACB_1$. Define

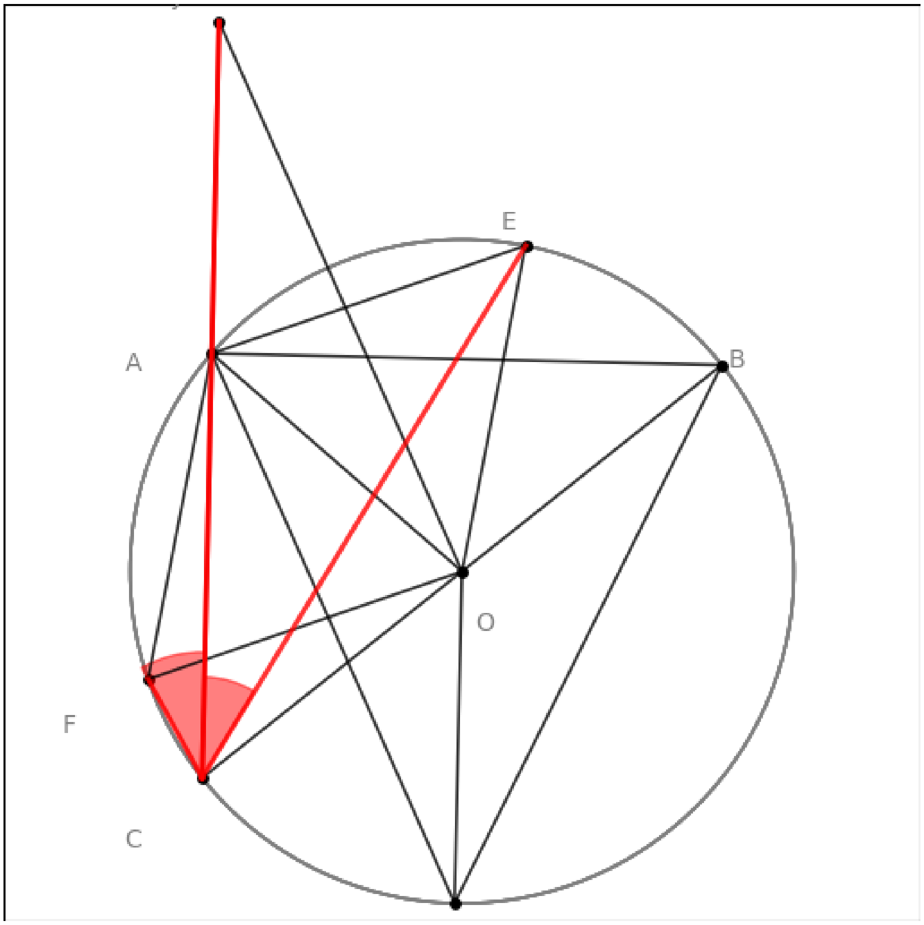

Figure 4: imo 2002 p2b

point D_1 on circle (O, A) such that $\measuredangle ACD_1 = \measuredangle D_1AC$. Define point X as the intersection of lines AC and BB_1. Define point D as the intersection of circle (O, A) and line D_1X. Define point P as the foot of D on line BC. Define point Q as the foot of D on line AC. Define point R as the foot of D on line AB. Prove that $PQ = QR$

Proof:

Step 1. A, C, Q are collinear, B, C, P are collinear, AC is perpendicular to DQ and BC is perpendicular to $DP \Rightarrow \measuredangle CPD = \measuredangle CQD$.

Step 2. $\measuredangle CPD = \measuredangle CQD \Rightarrow C, D, P, Q$ are cyclic.

Step 3. C, D, P, Q are cyclic $\Rightarrow \measuredangle CDQ = \measuredangle CPQ$.

Step 4. $AO = BO$, $AO = B_1O$ and $BO = CO \Rightarrow A, B, B_1, C$ are cyclic.

Step 5. $AO = BO$, $AO = B_1O$, $AO = DO$ and A, B, B_1, C are cyclic $\Rightarrow$ A, B, C, D are cyclic.

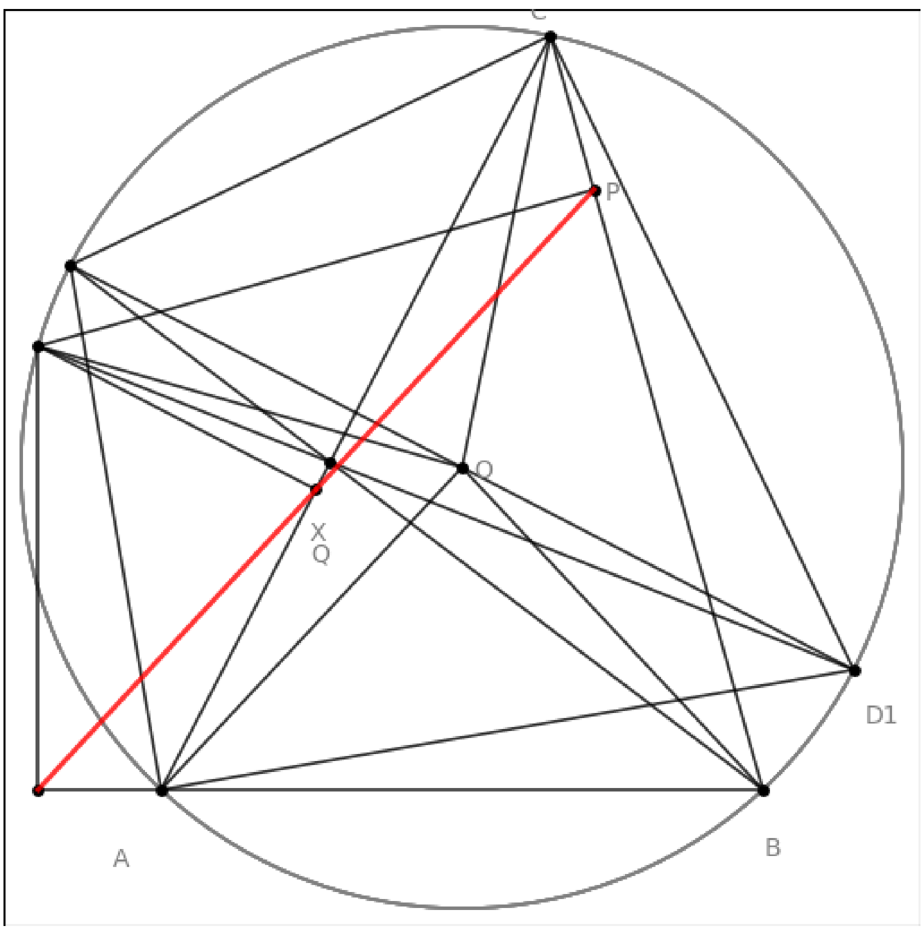

Figure 5: imo 2003 p4

Step 6. A, B, C, D are cyclic $\Rightarrow \measuredangle BAD = \measuredangle BCD$, $\measuredangle ACB = \measuredangle ADB$ and $\measuredangle BAC = \measuredangle BDC$.

Step 7. A, C, X are collinear and AC is perpendicular to DQ $\Rightarrow$ AX is perpendicular to DQ.

Step 8. B, C, P are collinear and BC is perpendicular to DP $\Rightarrow$ BP is perpendicular to DP.

Step 9. AX is perpendicular to DQ and BP is perpendicular to DP $\Rightarrow$ $\measuredangle(AX, BP) = \measuredangle QDP$.

Step 10. B, C, P are collinear, $\measuredangle BAD = \measuredangle BCD$ and $\measuredangle CDQ = \measuredangle CPQ \Rightarrow \measuredangle BAD = \measuredangle PQD$.

Step 11. A, C, X are collinear, B, C, P are collinear, $\measuredangle(AX, BP) = \measuredangle QDP$ and $\measuredangle ACB = \measuredangle ADB \Rightarrow \measuredangle ADB = \measuredangle QDP$.

Step 12. $\measuredangle ADB = \measuredangle QDP$ and $\measuredangle BAD = \measuredangle PQD \Rightarrow \frac{BA}{BD} = \frac{PQ}{PD}$.

Step 13. A, B, R are collinear, A, C, Q are collinear, AB is perpendicular to DR and AC is perpendicular to $DQ \Rightarrow \measuredangle AQD = \measuredangle ARD$.

Step 14. $\measuredangle AQD = \measuredangle ARD \Rightarrow A, D, Q, R$ are cyclic.

Step 15. A, D, Q, R are cyclic $\Rightarrow \measuredangle ADQ = \measuredangle ARQ$.

Step 16. A, B, R are collinear and AB is perpendicular to $DR \Rightarrow AR$ is perpendicular to DR.

Step 17. AR is perpendicular to DR and AX is perpendicular to $DQ \Rightarrow \measuredangle XAR = \measuredangle QDR$.

Step 18. A, B, R are collinear, $\measuredangle BAD = \measuredangle BCD$ and $\measuredangle ADQ = \measuredangle ARQ \Rightarrow \measuredangle BCD = \measuredangle RQD$.

Step 19. A, B, R are collinear, A, C, X are collinear, $\measuredangle BAC = \measuredangle BDC$ and $\measuredangle XAR = \measuredangle QDR \Rightarrow \measuredangle BDC = \measuredangle RDQ$.

Step 20. $\measuredangle BDC = \measuredangle RDQ$ and $\measuredangle BCD = \measuredangle RQD \Rightarrow \frac{BC}{BD} = \frac{RQ}{RD}$.

Step 21. AR is perpendicular to DR and BP is perpendicular to $DP \Rightarrow \measuredangle(AR, BP) = \measuredangle RDP$.

Step 22. A, B, R are collinear, B, C, P are collinear and $\measuredangle BAD = \measuredangle BCD \Rightarrow \measuredangle DAR = \measuredangle DCP$.

Step 23. B, C, P are collinear and $\measuredangle(AR, BP) = \measuredangle RDP \Rightarrow \measuredangle ARD = \measuredangle CPD$.

Step 24. $\measuredangle DAR = \measuredangle DCP$ and $\measuredangle ARD = \measuredangle CPD \Rightarrow \frac{DA}{DR} = \frac{DC}{DP}$.

Step 25. $AO = BO$, $AO = B_1O$, $AO = DO$, $AO = D_1O$ and A, B, B_1, C are cyclic $\Rightarrow A, C, D, D_1$ are cyclic.

Step 26. A, C, D, D_1 are cyclic $\Rightarrow \measuredangle ACD_1 = \measuredangle ADD_1$ and $\measuredangle ACD = \measuredangle AD_1D$.

Step 27. D, D_1, X are collinear, $\measuredangle ACD = \measuredangle AD_1D$, $\measuredangle ACD_1 = \measuredangle D_1AC$ and $\measuredangle ACD_1 = \measuredangle ADD_1 \Rightarrow DX$ is the bisector of $\measuredangle ADC$.

Step 28. A, C, X are collinear and DX is the bisector of $\measuredangle ADC \Rightarrow \frac{DA}{DC} = \frac{XA}{XC}$.

Step 29. A, B, B_1, C are cyclic $\Rightarrow \measuredangle AB_1B = \measuredangle ACB$ and $\measuredangle ABB_1 = \measuredangle ACB_1$.

Step 30. B, B_1, X are collinear, $\measuredangle B_1AC = \measuredangle ACB_1$, $\measuredangle ABB_1 = \measuredangle ACB_1$ and $\measuredangle AB_1B = \measuredangle ACB \Rightarrow BX$ is the bisector of $\measuredangle ABC$.

Step 31. A, C, X are collinear and BX is the bisector of $\measuredangle ABC \Rightarrow \frac{BA}{BC} = \frac{XA}{XC}$.

Step 32. $\frac{BA}{BC} = \frac{XA}{XC}$, $\frac{BA}{BD} = \frac{PQ}{PD}$, $\frac{BC}{BD} = \frac{RQ}{RD}$, $\frac{DA}{DC} = \frac{XA}{XC}$ and $\frac{DA}{DR} = \frac{DC}{DP} \Rightarrow$ by ratio chasing: $PQ = QR$

■

2.6 IMO 2004 P1

Original:

Let ABC be a triangle with AB = AC. The circle with diameter BC intersects the sides AB and AC at M and N respectively. Denote by O the midpoint of the side BC. The bisectors of the angles $\measuredangle BAC$ and $\measuredangle MON$ intersect at R. Prove that the circumcircles of the triangles BMR and CNR have a common point lying on the side BC.

Translated:

Let $ABCO$ be a quadrilateral. Define point M as the intersection of circle (O, B) and line AB. Define point N as the intersection of circle (O, B) and line AC. Define point R such that AR is the bisector of $\measuredangle BAC$ and OR is the bisector of $\measuredangle MON$. Define point O_1 as the circumcenter of triangle RBM. Define point O_2 as the circumcenter of triangle RCN. Define point P as the intersection of circles (O_1, R) and (O_2, R). Prove that B, C, P are collinear

Proof:

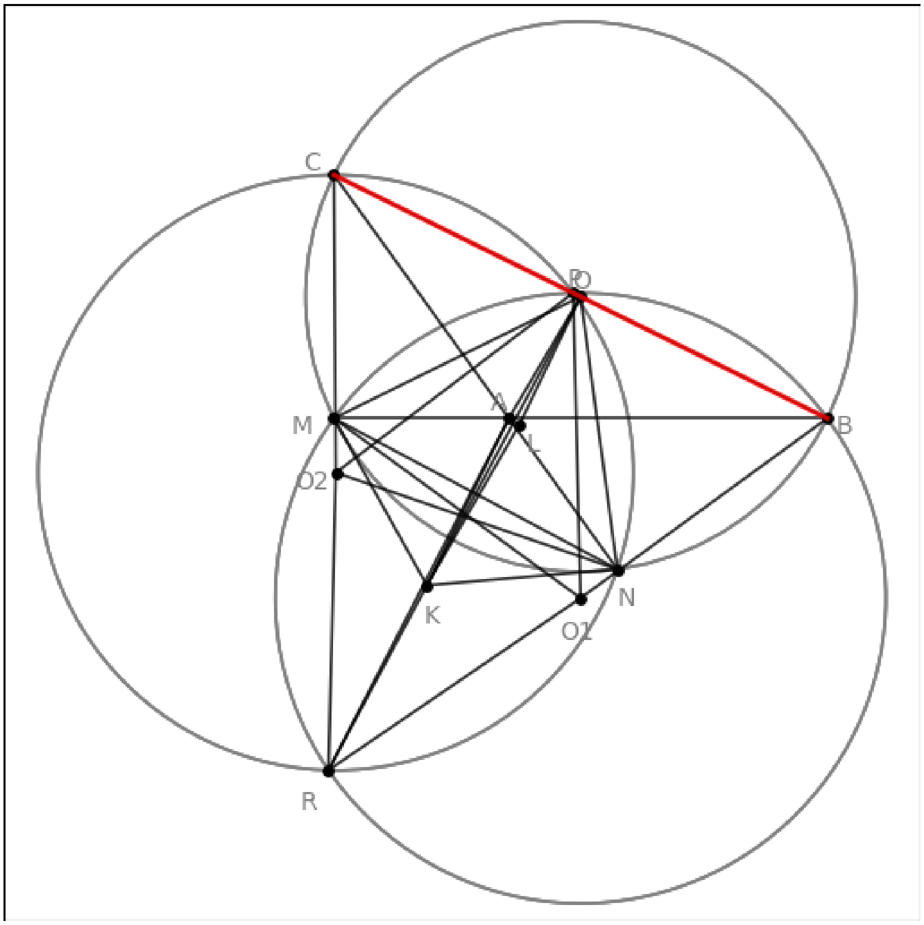

Figure 6: imo 2004 p1

Construct point K such that $KM = KN$.
Construct point L as the intersection of circles (K, A) and (O, A).
Step 1. $AK = KL$ and $AO = LO \Rightarrow AL$ is perpendicular to KO.
Step 2. $AK = KL$ and $AO = LO \Rightarrow KO$ is the bisector of $\measuredangle AKL$.
Step 3. $BO = MO$ and $BO = NO \Rightarrow MO = NO$.
Step 4. $KM = KN$ and $MO = NO \Rightarrow KO$ is perpendicular to MN.

Step 5. $MO = NO$ and OR is the bisector of $\measuredangle MON \Rightarrow MR = NR$.

Step 6. $MO = NO$ and $MR = NR \Rightarrow MN$ is perpendicular to OR.

Step 7. AL is perpendicular to KO and KO is perpendicular to $MN \Rightarrow AL$ is parallel to MN.

Step 8. KO is the bisector of $\measuredangle AKL$, AL is parallel to MN, AL is perpendicular to KO and MN is perpendicular to $OR \Rightarrow KR$ is the bisector of $\measuredangle AKL$.

Step 9. $AK = KL$ and KR is the bisector of $\measuredangle AKL \Rightarrow AR = LR$.

Step 10. $MO = NO \Rightarrow \measuredangle MNO = \measuredangle OMN$.

Step 11. $AO = LO \Rightarrow \measuredangle ALO = \measuredangle OAL$.

Step 12. $\measuredangle ALO = \measuredangle OAL$, $\measuredangle MNO = \measuredangle OMN$ and AL is parallel to $MN \Rightarrow$ by angle chasing: $\measuredangle AOM = \measuredangle NOL$ and $\measuredangle AON = \measuredangle MOL$.

Step 13. $AO = LO$, $MO = NO$ and $\measuredangle AOM = \measuredangle NOL \Rightarrow AM = LN$.

Step 14. $AM = LN$, $AR = LR$ and $MR = NR \Rightarrow \measuredangle MAR = \measuredangle RLN$.

Step 15. A, B, M are collinear, A, C, N are collinear, AR is the bisector of $\measuredangle BAC$ and $\measuredangle MAR = \measuredangle RLN \Rightarrow \measuredangle NAR = \measuredangle NLR$.

Step 16. $\measuredangle NAR = \measuredangle NLR \Rightarrow A, L, N, R$ are cyclic.

Step 17. $AO = LO$, $MO = NO$ and $\measuredangle AON = \measuredangle MOL \Rightarrow AN = LM$.

Step 18. $AN = LM$, $AR = LR$ and $MR = NR \Rightarrow \measuredangle RAN = \measuredangle MLR$.

Step 19. A, B, M are collinear, A, C, N are collinear, AR is the bisector of $\measuredangle BAC$ and $\measuredangle RAN = \measuredangle MLR \Rightarrow \measuredangle MAR = \measuredangle MLR$.

Step 20. $\measuredangle MAR = \measuredangle MLR \Rightarrow A, L, M, R$ are cyclic.

Step 21. A, L, M, R are cyclic and A, L, N, R are cyclic $\Rightarrow A, M, N, R$ are cyclic.

Step 22. A, M, N, R are cyclic $\Rightarrow \measuredangle MAN = \measuredangle MRN$.

Step 23. $BO_1 = MO_1$, $MO_1 = O_1R$ and $O_1P = O_1R \Rightarrow B, M, P, R$ are cyclic.

Step 24. B, M, P, R are cyclic $\Rightarrow \measuredangle BMR = \measuredangle BPR$.

Step 25. $CO_2 = NO_2$, $NO_2 = O_2R$ and $O_2P = O_2R \Rightarrow C, N, P, R$ are cyclic.

Step 26. C, N, P, R are cyclic $\Rightarrow \measuredangle NCP = \measuredangle NRP$.

Step 27. A, B, M are collinear, A, C, N are collinear, $\measuredangle MAN = \measuredangle MRN$, $\measuredangle NCP = \measuredangle NRP$ and $\measuredangle BMR = \measuredangle BPR \Rightarrow \measuredangle BPR = \measuredangle CPR$.

Step 28. $\measuredangle BPR = \measuredangle CPR \Rightarrow BP$ is parallel to CP.

Step 29. BP is parallel to $CP \Rightarrow B, C, P$ are collinear

■

2.7 IMO 2004 P5A

Original:

In a convex quadrilateral ABCD, the diagonal BD bisects neither the angle ABC nor the angle CDA. The point P lies inside ABCD and satisfies $\measuredangle PBC = \measuredangle DBA$ and $\measuredangle PDC = \measuredangle BDA$. Prove that AP=CP given ABCD is a cyclic quadrilateral.

Translated:

Let ABC be a triangle. Define point O as the circumcenter of triangle CBA. Let D be any point on circle (O, A). Define point P such that $\measuredangle ABD = \measuredangle PBC$ and $\measuredangle ADB = \measuredangle PDC$. Prove that $AP = CP$

Proof:

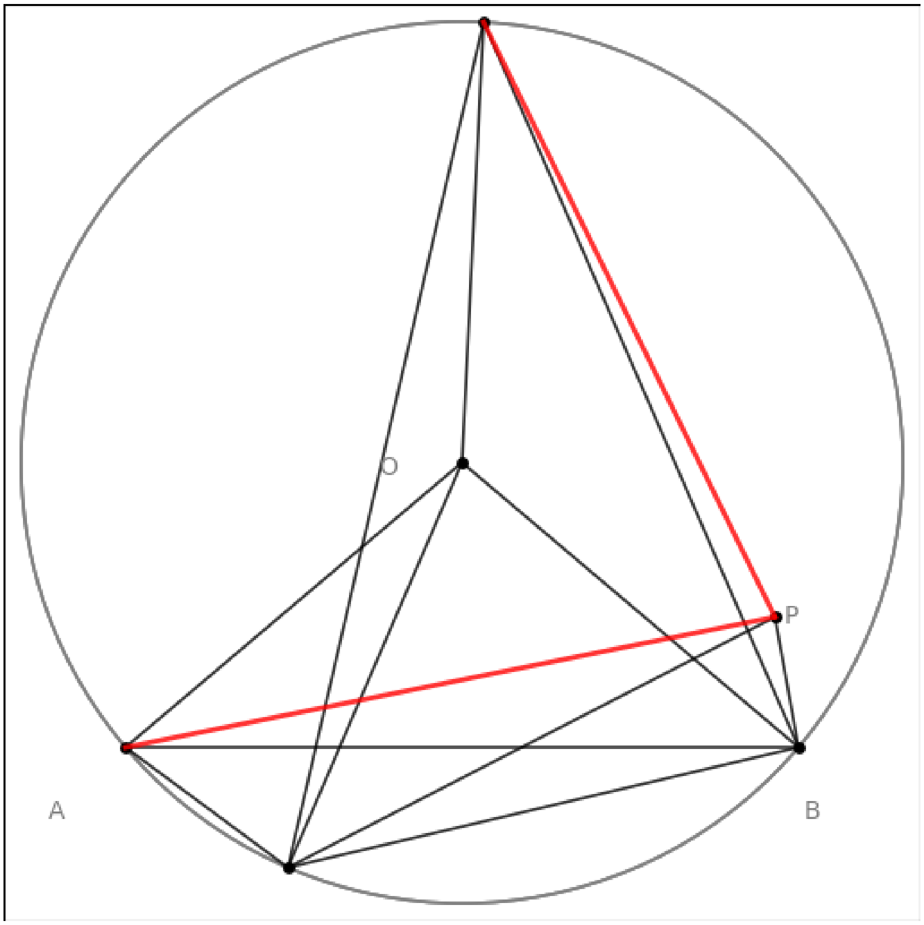

Figure 7: imo 2004 p5a

Step 1. $AO = BO$, $AO = DO$ and $BO = CO \Rightarrow A, B, C, D$ are cyclic.

Step 2. A, B, C, D are cyclic $\Rightarrow \measuredangle BAD = \measuredangle BCD$ and $\measuredangle BAC = \measuredangle BDC$.

Step 3. $AO = BO$, $AO = DO$ and $BO = CO \Rightarrow CO = DO$.

Step 4. $CO = DO \Rightarrow \measuredangle CDO = \measuredangle OCD$.

Step 5. $BO = CO \Rightarrow \measuredangle BCO = \measuredangle OBC$.

Step 6. $\measuredangle BAD = \measuredangle BCD$, $\measuredangle ABD = \measuredangle PBC$, $\measuredangle BCO = \measuredangle OBC$, $\measuredangle ADB = \measuredangle PDC$ and $\measuredangle CDO = \measuredangle OCD \Rightarrow$ by angle chasing: $\measuredangle BOD = \measuredangle BPD$.

Step 7. $\measuredangle BOD = \measuredangle BPD \Rightarrow B, D, O, P$ are cyclic.

Step 8. B, D, O, P are cyclic $\Rightarrow \measuredangle BDP = \measuredangle BOP$.

Step 9. $AO = BO$ and $BO = CO \Rightarrow AO = CO$.

Step 10. $AO = CO \Rightarrow \measuredangle ACO = \measuredangle OAC$.

Step 11. $AO = BO \Rightarrow \measuredangle ABO = \measuredangle OAB$.

Step 12. $\measuredangle BAC = \measuredangle BDC$, $\measuredangle BAD = \measuredangle BCD$, $\measuredangle ABO = \measuredangle OAB$, $\measuredangle ACO = \measuredangle OAC$, $\measuredangle BCO = \measuredangle OBC$, $\measuredangle ADB = \measuredangle PDC$ and $\measuredangle BDP = \measuredangle BOP \Rightarrow$ by angle chasing: OP is the bisector of $\measuredangle AOC$.

Step 13. $AO = CO$ and OP is the bisector of $\measuredangle AOC \Rightarrow AP = CP$

■

2.8 IMO 2005 P5A

Original:

Let ABCD be a fixed convex quadrilateral with BC = DA and $BCDA$. Let two variable points E and F lie on the sides BC and DA, respectively, and satisfy BE = DF. The lines AC and BD meet at P, the lines BD and EF meet at Q, the lines EF and AC meet at R. Let O1 and O2 be the circumcircle of triangles APD and BPC respectively, and M be the second intersection of the circles (O1,P) and (O2,P). Prove that the circumcircles of the triangles PQR, as E and F vary, always go through M.

Translated:

Let ABC be a triangle. Define point D such that $AD = BC$. Let E be any point on line BC. Define point F on line AD such that $BE = DF$. Define point P as the intersection of lines AC and BD. Define point Q as the intersection of lines BD and EF. Define point R as the intersection of lines AC and EF. Define point O_1 as the circumcenter of triangle PDA. Define point O_2 as the circumcenter of triangle CBP. Define point M as the intersection of circles (O_1, P) and (O_2, P). Prove that M, P, Q, R are cyclic

Proof:

Step 1. $AO_1 = O_1P$, $DO_1 = O_1P$ and $MO_1 = O_1P \Rightarrow A, D, M, P$ are cyclic.

Step 2. A, D, M, P are cyclic $\Rightarrow \measuredangle DAP = \measuredangle DMP$ and $\measuredangle MAP = \measuredangle MDP$.

Step 3. $BO_2 = O_2P$, $CO_2 = O_2P$ and $MO_2 = O_2P \Rightarrow B, C, M, P$ are cyclic.

Step 4. B, C, M, P are cyclic $\Rightarrow \measuredangle BCP = \measuredangle BMP$ and $\measuredangle MBP = \measuredangle MCP$.

Step 5. A, C, P are collinear, A, D, F are collinear and $\measuredangle DAP = \measuredangle DMP \Rightarrow \measuredangle(AF, CP) = \measuredangle DMP$.

Step 6. B, C, E are collinear and $\measuredangle BCP = \measuredangle BMP \Rightarrow \measuredangle(BE, CP) = \measuredangle BMP$.

Step 7. $\measuredangle(AF, CP) = \measuredangle DMP$ and $\measuredangle(BE, CP) = \measuredangle BMP \Rightarrow \measuredangle(AF, BE) = \measuredangle DMB$.

Step 8. A, D, F are collinear, B, C, E are collinear and $\measuredangle(AF, BE) = \measuredangle DMB \Rightarrow \measuredangle ADM = \measuredangle CBM$.

Step 9. A, C, P are collinear, B, D, P are collinear, $\measuredangle MAP = \measuredangle MDP$ and $\measuredangle MBP = \measuredangle MCP \Rightarrow \measuredangle AMD = \measuredangle CMB$.

Step 10. $\measuredangle AMD = \measuredangle CMB$ and $\measuredangle ADM = \measuredangle CBM \Rightarrow \frac{AD}{BC} = \frac{MD}{MB}$.

Step 11. A, D, F are collinear, B, C, E are collinear and $\measuredangle ADM = \measuredangle CBM \Rightarrow \measuredangle EBM = \measuredangle FDM$.

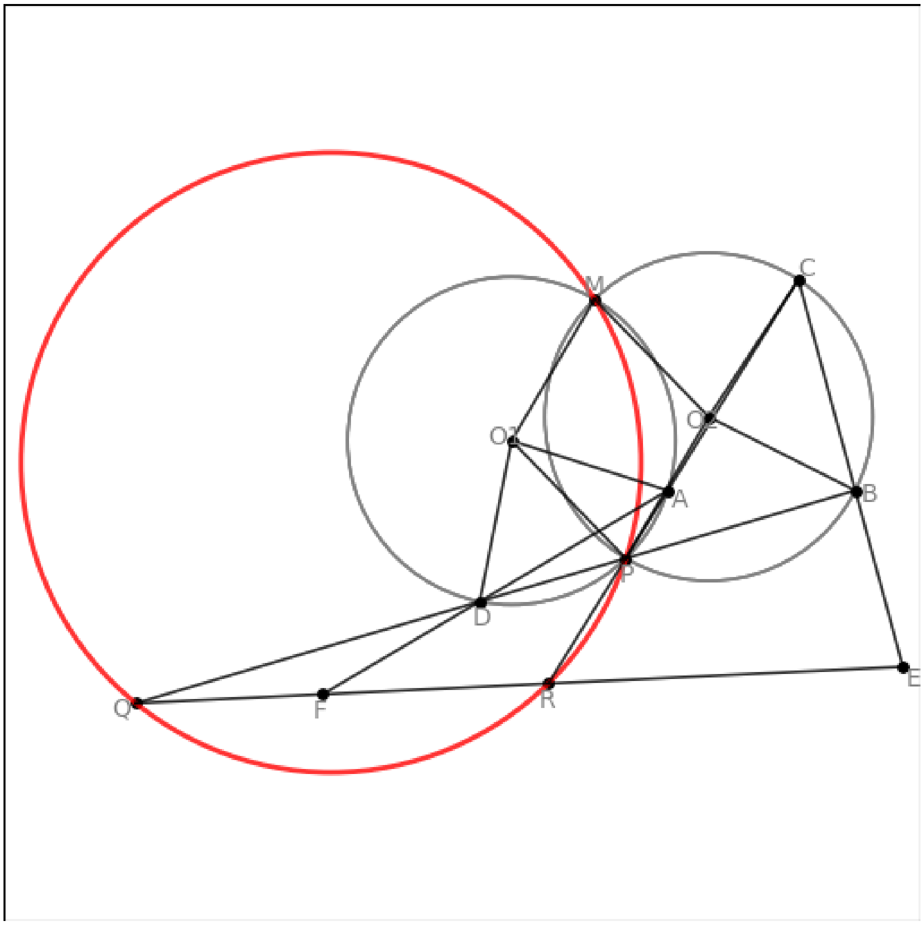

Figure 8: imo 2005 p5a

Step 12. $AD = BC$ and $\frac{AD}{BC} = \frac{MD}{MB} \Rightarrow BM = DM$.

Step 13. $BE = DF$, $BM = DM$ and $\measuredangle EBM = \measuredangle FDM \Rightarrow EM = FM$ and $\measuredangle BMD = \measuredangle EMF$.

Step 14. $BM = DM$ and $EM = FM \Rightarrow \frac{MB}{MD} = \frac{ME}{MF}$.

Step 15. $\measuredangle BMD = \measuredangle EMF$ and $\frac{MB}{MD} = \frac{ME}{MF} \Rightarrow \measuredangle BDM = \measuredangle EFM$.

Step 16. B, D, Q are collinear, E, F, Q are collinear and $\measuredangle BDM = \measuredangle EFM \Rightarrow \measuredangle MDQ = \measuredangle MFQ$.

Step 17. $\measuredangle MDQ = \measuredangle MFQ \Rightarrow D, F, M, Q$ are cyclic.

Step 18. D, F, M, Q are cyclic $\Rightarrow \measuredangle DFQ = \measuredangle DMQ$.

Step 19. A, C, P are collinear, A, C, R are collinear, A, D, F are collinear, E, F, Q are collinear, E, F, R are collinear, $\measuredangle DAP = \measuredangle DMP$ and $\measuredangle DFQ = \measuredangle DMQ \Rightarrow \measuredangle MPR = \measuredangle MQR$.

Step 20. $\measuredangle MPR = \measuredangle MQR \Rightarrow M, P, Q, R$ are cyclic

■

2.9 IMO 2007 P4

Original:

In triangle ABC the bisector of $\measuredangle BCA$ meets the circumcircle again at R, the perpendicular bisector of BC at P, and the perpendicular bisector of AC at Q. The midpoint of BC is K and the midpoint of AC is L. Prove that the triangles RPK and RQL have the same area.

Translated:

Let ABC be a triangle. Define point O as the circumcenter of triangle CBA. Define point R on circle (O, A) such that $AR = BR$ and $\measuredangle ABR = \measuredangle RAB$. Define point L as the midpoint of AC. Define point K as the midpoint of BC. Define point P as the intersection of lines CR and KO. Define point Q as the intersection of lines CR and LO. Define point L_1 as the foot of L on line CR. Define point K_1 as the foot of K on line CR. Prove that $\frac{KK_1}{LL_1} = \frac{RQ}{RP}$

Proof:

Step 1. $AO = BO$, $AO = OR$ and $BO = CO \Rightarrow O$ is the circumcenter of ACR.

Step 2. A, C, L are collinear and $AL = CL \Rightarrow L$ is the midpoint of AC.

Step 3. O is the circumcenter of ACR and L is the midpoint of $AC \Rightarrow \measuredangle AOL = \measuredangle ARC$.

Step 4. $AO = BO$, $AO = OR$ and $BO = CO \Rightarrow O$ is the circumcenter of BCR.

Step 5. B, C, K are collinear and $BK = CK \Rightarrow K$ is the midpoint of BC.

Step 6. O is the circumcenter of BCR and K is the midpoint of $BC \Rightarrow \measuredangle BOK = \measuredangle BRC$.

Step 7. $AO = BO$ and $AR = BR \Rightarrow \measuredangle RAO = \measuredangle OBR$.

Step 8. C, K_1, R are collinear, C, L_1, R are collinear, C, P, R are collinear, C, Q, R are collinear, K, O, P are collinear, L, O, Q are collinear, $\measuredangle RAO = \measuredangle OBR$, $\measuredangle AOL = \measuredangle ARC$ and $\measuredangle BOK = \measuredangle BRC \Rightarrow \measuredangle KPK_1 = \measuredangle L_1QL$.

Step 9. C, K_1, R are collinear, C, L_1, R are collinear, C, P, R are collinear, C, Q, R are collinear, CR is perpendicular to KK_1 and CR is perpendicular to $LL_1 \Rightarrow \measuredangle KK_1P = \measuredangle QL_1L$.

Step 10. $\measuredangle KK_1P = \measuredangle QL_1L$ and $\measuredangle KPK_1 = \measuredangle L_1QL \Rightarrow \frac{KK_1}{LL_1} = \frac{KP}{LQ}$.

Step 11. $AO = BO$, $AO = OR$ and $BO = CO \Rightarrow A, B, C, R$ are cyclic.

Step 12. A, B, C, R are cyclic $\Rightarrow \measuredangle ABR = \measuredangle ACR$ and $\measuredangle BAR = \measuredangle BCR$.

Step 13. $AO = BO$ and $BO = CO \Rightarrow AO = CO$.

Step 14. $AL = CL$ and $AO = CO \Rightarrow AC$ is perpendicular to LO.

Step 15. $BK = CK$ and $BO = CO \Rightarrow BC$ is perpendicular to KO.

Step 16. A, C, L are collinear, B, C, K are collinear, C, P, R are collinear, C, Q, R are collinear, $\measuredangle BAR = \measuredangle BCR$, $\measuredangle ABR = \measuredangle RAB$ and $\measuredangle ABR = \measuredangle ACR$ $\Rightarrow \measuredangle KCP = \measuredangle QCL$.

Step 17. A, C, L are collinear, B, C, K are collinear, K, O, P are collinear, L, O, Q are collinear, AC is perpendicular to LO and BC is perpendicular to $KO \Rightarrow \measuredangle CLQ = \measuredangle PKC$.

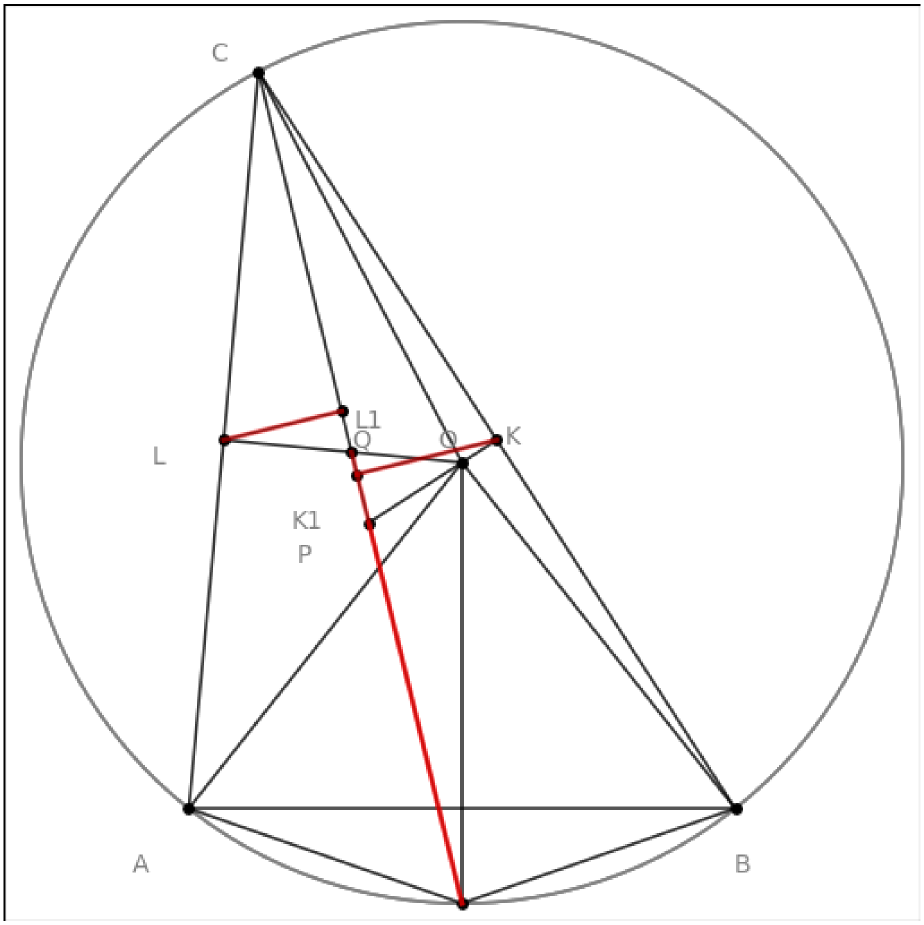

Figure 9: imo 2007 p4

Step 18. $\measuredangle KCP = \measuredangle QCL$ and $\measuredangle CLQ = \measuredangle PKC \Rightarrow \frac{CP}{CQ} = \frac{KP}{LQ}$.

Step 19. $AO = BO$, $AO = OR$ and $BO = CO \Rightarrow CO = OR$.

Step 20. $CO = OR \Rightarrow \measuredangle OCR = \measuredangle CRO$.

Step 21. C, P, R are collinear, C, Q, R are collinear, K, O, P are collinear, L, O, Q are collinear, $\measuredangle RAO = \measuredangle OBR$, $\measuredangle AOL = \measuredangle ARC$ and $\measuredangle BOK = \measuredangle BRC$ $\Rightarrow \measuredangle CQO = \measuredangle OPR$.

Step 22. C, P, R are collinear, C, Q, R are collinear and $\measuredangle OCR = \measuredangle CRO \Rightarrow$ $\measuredangle OCQ = \measuredangle PRO$.

Step 23. $\measuredangle OCQ = \measuredangle PRO$ and $\measuredangle CQO = \measuredangle OPR \Rightarrow \frac{OC}{OR} = \frac{CQ}{PR}$.

Step 24. C, P, R are collinear, C, Q, R are collinear, K, O, P are collinear, L, O, Q are collinear, $\measuredangle RAO = \measuredangle OBR$, $\measuredangle AOL = \measuredangle ARC$ and $\measuredangle BOK = \measuredangle BRC$ $\Rightarrow \measuredangle CPO = \measuredangle OQR$.

Step 25. C, P, R are collinear, C, Q, R are collinear and $\measuredangle OCR = \measuredangle CRO \Rightarrow$

$\measuredangle PCO = \measuredangle ORQ$.

Step 26. $\measuredangle PCO = \measuredangle ORQ$ and $\measuredangle CPO = \measuredangle OQR \Rightarrow \frac{OC}{OR} = \frac{CP}{QR}$.

Step 27. $\frac{CP}{CQ} = \frac{KP}{LQ}$, $\frac{KK_1}{LL_1} = \frac{KP}{LQ}$, $\frac{OC}{OR} = \frac{CP}{QR}$ and $\frac{OC}{OR} = \frac{CQ}{PR} \Rightarrow \frac{KK_1}{LL_1} = \frac{RQ}{RP}$

■

2.10 IMO 2008 P1A

Original:

Let H be the orthocenter of a triangle ABC. The circle GammaA centered at the midpoint of BC and passing through H intersects the sideline BC at points A1 and A2. Similarly,define the points B1, B2, C1, and C2. Prove that C1 C2 B1 B2 are concyclic.

Translated:

Let ABC be a triangle. Define point H such that AH is perpendicular to BC. Define point E as the midpoint of AC. Define point F as the midpoint of AB. Define point B_1 as the intersection of circle (E, H) and line AC. Define point B_2 as the intersection of circle (E, H) and line AC. Define point C_1 as the intersection of circle (F, H) and line AB. Define point C_2 as the intersection of circle (F, H) and line AB. Prove that B_1, B_2, C_1, C_2 are cyclic

Proof:

Construct point O such that $HE = EO$ and HO is perpendicular to EF.

Step 1. $HE = EB_1$, $HE = EB_2$ and $HE = EO \Rightarrow H, B_1, B_2, O$ are cyclic.

Step 2. H, B_1, B_2, O are cyclic $\Rightarrow \measuredangle B_2HO = \measuredangle B_2B_1O$ and $\measuredangle HB_1B_2 = \measuredangle HOB_2$.

Step 3. $HE = EB_2 \Rightarrow \measuredangle EHB_2 = \measuredangle HB_2E$.

Step 4. A, C, E are collinear, A, C, B_1 are collinear, A, C, B_2 are collinear and $\measuredangle B_2HO = \measuredangle B_2B_1O \Rightarrow \measuredangle B_2HO = \measuredangle(EB_2, B_1O)$.

Step 5. $\measuredangle EHB_2 = \measuredangle HB_2E$ and $\measuredangle B_2HO = \measuredangle(EB_2, B_1O) \Rightarrow \measuredangle EHB_2 = \measuredangle HOB_1$.

Step 6. A, C, E are collinear and $AE = CE \Rightarrow E$ is the midpoint of AC.

Step 7. A, B, F are collinear and $AF = BF \Rightarrow F$ is the midpoint of AB.

Step 8. E is the midpoint of AC and F is the midpoint of $AB \Rightarrow BC$ is parallel to EF.

Step 9. $HE = EB_1$, $HE = EB_2$ and $HE = EO \Rightarrow E$ is the circumcenter of B_1B_2O.

Step 10. A, C, E are collinear, A, C, B_1 are collinear and A, C, B_2 are collinear $\Rightarrow E, B_1, B_2$ are collinear.

Step 11. E is the circumcenter of B_1B_2O and E, B_1, B_2 are collinear $\Rightarrow B_1O$ is perpendicular to B_2O.

Step 12. BC is parallel to EF and AH is perpendicular to $BC \Rightarrow AH$ is perpendicular to EF.

Step 13. AH is perpendicular to EF and B_1O is perpendicular to $B_2O \Rightarrow \measuredangle(AH, B_1O) = \measuredangle(EF, B_2O)$.

Step 14. AH is perpendicular to BC and HO is perpendicular to $EF \Rightarrow \measuredangle(AH, BC) = \measuredangle(HO, EF)$.

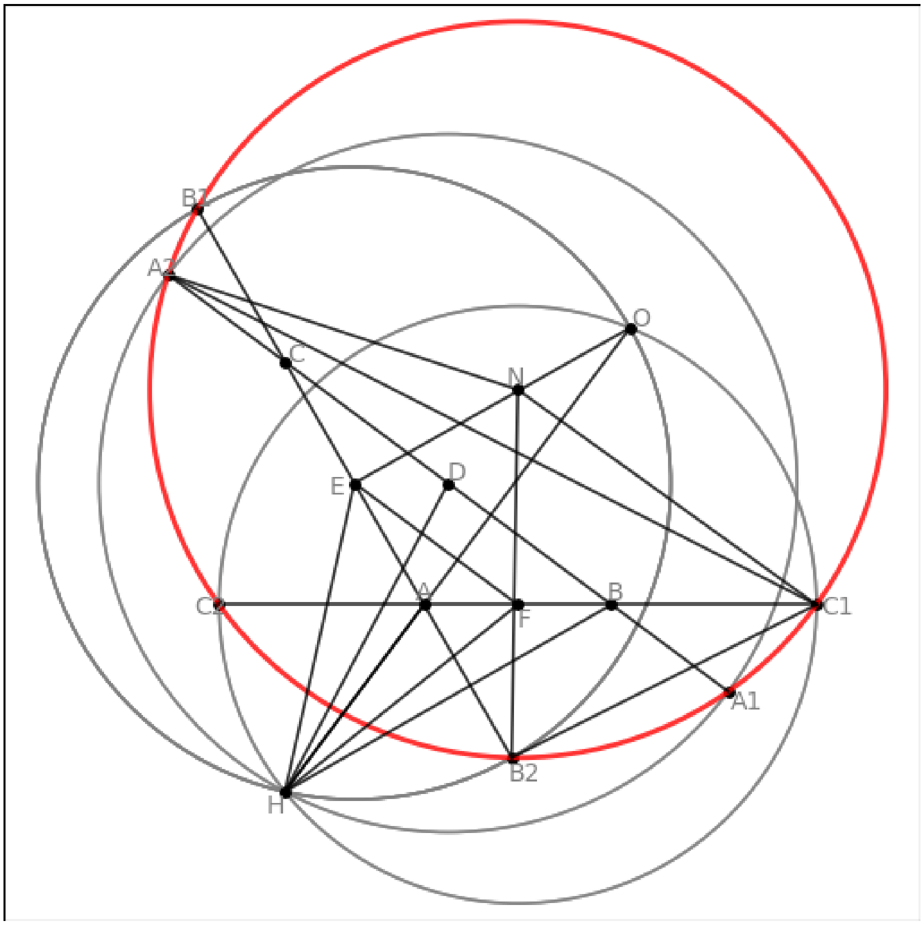

Figure 10: imo 2008 p1a

Step 15. $\measuredangle(AH, BC) = \measuredangle(HO, EF)$ and BC is parallel to $EF \Rightarrow \measuredangle(AH, EF) = \measuredangle(HO, EF)$.

Step 16. $\measuredangle(AH, EF) = \measuredangle(HO, EF) \Rightarrow AH$ is parallel to HO.

Step 17. $HE = EB_1$ and $HE = EB_2 \Rightarrow E$ is the circumcenter of HB_1B_2.

Step 18. E is the circumcenter of HB_1B_2 and E, B_1, B_2 are collinear $\Rightarrow$ HB_1 is perpendicular to HB_2.

Step 19. A, C, E are collinear, A, C, B_1 are collinear, A, C, B_2 are collinear and $\measuredangle HB_1B_2 = \measuredangle HOB_2 \Rightarrow \measuredangle(HB_1, EB_2) = \measuredangle HOB_2$.

Step 20. HB_1 is perpendicular to HB_2 and HO is perpendicular to $EF \Rightarrow$ $\measuredangle B_1HB_2 = \measuredangle(HO, EF)$.

Step 21. $\measuredangle B_1HB_2 = \measuredangle(HO, EF)$ and $\measuredangle(HB_1, EB_2) = \measuredangle HOB_2 \Rightarrow \measuredangle HB_2E = \measuredangle(EF, B_2O)$.

Step 22. $HE = EB_2$ and $HE = EO \Rightarrow EB_2 = EO$.

Step 23. $EB_2 = EO \Rightarrow \measuredangle EB_2O = \measuredangle B_2OE$.

Step 24. $\measuredangle(AH, B_1O) = \measuredangle(EF, B_2O)$, $\measuredangle EHB_2 = \measuredangle HOB_1$ and AH is parallel to $HO \Rightarrow \measuredangle EHB_2 = \measuredangle(EF, B_2O)$.

Step 25. $\measuredangle HB_2E = \measuredangle(EF, B_2O)$ and $\measuredangle EB_2O = \measuredangle B_2OE \Rightarrow \measuredangle(HB_2, EF) = \measuredangle B_2OE$.

Step 26. $\measuredangle EHB_2 = \measuredangle(EF, B_2O)$ and $\measuredangle(HB_2, EF) = \measuredangle B_2OE \Rightarrow EF$ is the bisector of $\measuredangle HEO$.

Step 27. $HE = EO$ and EF is the bisector of $\measuredangle HEO \Rightarrow HF = FO$.

Step 28. $HF = FC_1$, $HF = FC_2$ and $HF = FO \Rightarrow H, C_1, C_2, O$ are cyclic.

Step 29. H, C_1, C_2, O are cyclic $\Rightarrow \measuredangle HC_1C_2 = \measuredangle HOC_2$.

Step 30. AH is parallel to $HO \Rightarrow A, H, O$ are collinear.

Step 31. A, B, C_1 are collinear, A, B, C_2 are collinear, A, H, O are collinear and $\measuredangle HC_1C_2 = \measuredangle HOC_2 \Rightarrow \measuredangle AOC_2 = \measuredangle HC_1A$ and $\measuredangle AC_2O = \measuredangle C_1HA$.

Step 32. $\measuredangle AC_2O = \measuredangle C_1HA$ and $\measuredangle AOC_2 = \measuredangle HC_1A \Rightarrow \frac{AH}{AC_1} = \frac{AC_2}{AO}$.

Step 33. A, C, B_1 are collinear, A, C, B_2 are collinear, A, H, O are collinear and $\measuredangle HB_1B_2 = \measuredangle HOB_2 \Rightarrow \measuredangle AOB_2 = \measuredangle HB_1A$.

Step 34. A, C, B_1 are collinear, A, C, B_2 are collinear, $\measuredangle HB_1B_2 = \measuredangle HOB_2$ and AH is parallel to $HO \Rightarrow \measuredangle AB_2O = \measuredangle B_1HA$.

Step 35. $\measuredangle AB_2O = \measuredangle B_1HA$ and $\measuredangle AOB_2 = \measuredangle HB_1A \Rightarrow \frac{AH}{AB_1} = \frac{AB_2}{AO}$.

Step 36. $\frac{AH}{AB_1} = \frac{AB_2}{AO}$ and $\frac{AH}{AC_1} = \frac{AC_2}{AO} \Rightarrow$ by ratio chasing: $\frac{AB_1}{AC_2} = \frac{AC_1}{AB_2}$.

Step 37. A, B, C_1 are collinear, A, B, C_2 are collinear, A, C, B_1 are collinear and A, C, B_2 are collinear $\Rightarrow \measuredangle B_1AC_2 = \measuredangle B_2AC_1$.

Step 38. $\measuredangle B_1AC_2 = \measuredangle B_2AC_1$ and $\frac{AB_1}{AC_2} = \frac{AC_1}{AB_2} \Rightarrow \measuredangle AB_1C_2 = \measuredangle B_2C_1A$.

Step 39. A, B, C_1 are collinear, A, B, C_2 are collinear, A, C, B_1 are collinear, A, C, B_2 are collinear and $\measuredangle AB_1C_2 = \measuredangle B_2C_1A \Rightarrow \measuredangle B_2B_1C_2 = \measuredangle B_2C_1C_2$.

Step 40. $\measuredangle B_2B_1C_2 = \measuredangle B_2C_1C_2 \Rightarrow B_1, B_2, C_1, C_2$ are cyclic

■

2.11 IMO 2008 P1B

Original:

Let H be the orthocenter of a triangle ABC. The circle GammaA centered at the midpoint of BC and passing through H intersects the sideline BC at points A1 and A2. Similarly,define the points B1, B2, C1, and C2. Prove that C1 C2 B1 A1 are concyclic.

Translated:

Let ABC be a triangle. Define point H as the orthocenter of triangle CBA. Define point D as the midpoint of BC. Define point E as the midpoint of AC. Define point F as the midpoint of AB. Define point A_1 as the intersection of circle (D, H) and line BC. Define point A_2 as the intersection of circle (D, H) and line BC. Define point B_1 as the intersection of circle (E, H) and line AC. Define point B_2 as the intersection of circle (E, H) and line AC. Define point C_1 as the intersection of circle (F, H) and line AB. Define point C_2 as the intersection of circle (F, H) and line AB. Prove that C_1, C_2, B_1, A_1 are cyclic

Proof:

Not solved.

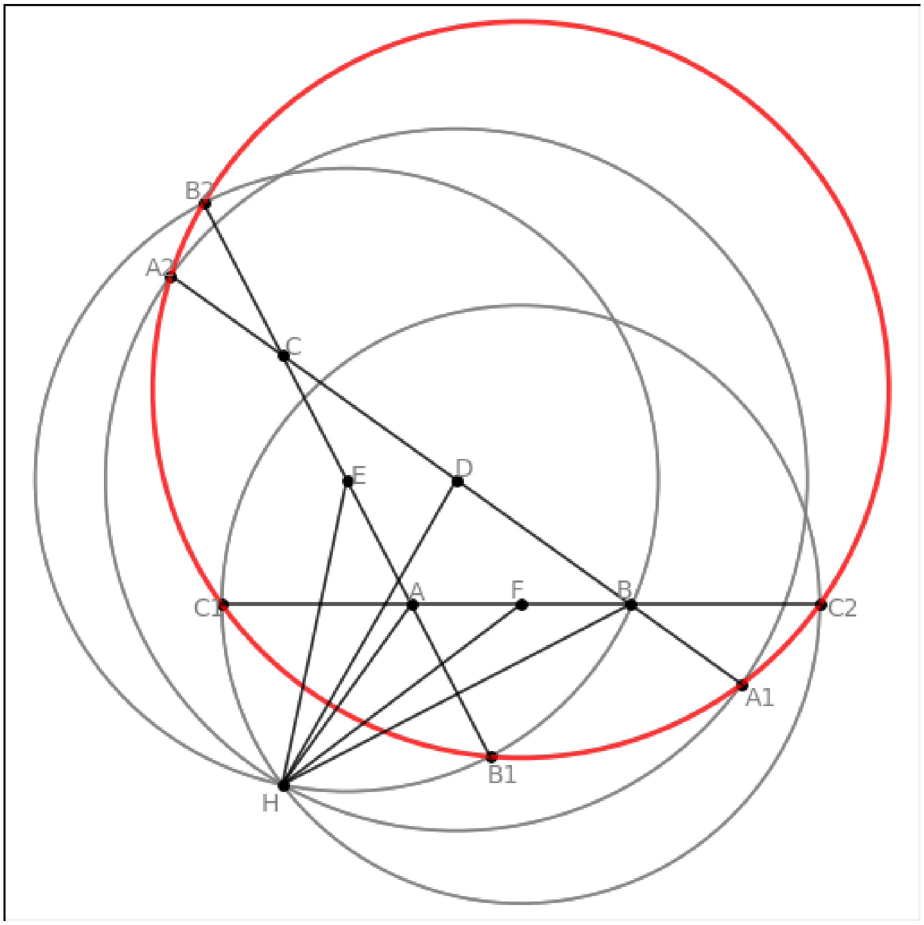

Figure 11: imo 2008 p1b

2.12 IMO 2008 P6

Original:

Let ABCD be a convex quadrilateral with $BA \neq BC$. Denote the incircles of triangles ABC and ADC by W1 and W2 respectively. Suppose that there exists a circle W tangent to ray BA beyond A and to the ray BC beyond C, which is also tangent to the lines AD and CD. Prove that the common external tangents to W1 and W2 intersect on W.

Translated:

Let XYZ be a triangle. Define point O as the circumcenter of triangle YXZ. Let W be any point on circle (O, X). Define point A such that AX is perpendicular to OX and AZ is perpendicular to OZ. Define point B such that BW is perpendicular to OW and BZ is perpendicular to OZ. Define point C such that CW is perpendicular to OW and CY is perpendicular to OY. Define

point D such that DX is perpendicular to OX and DY is perpendicular to OY. Define point I_1 such that AI_1 is the bisector of $\measuredangle BAC$ and CI_1 is the bisector of $\measuredangle ACB$. Define point I_2 such that AI_2 is the bisector of $\measuredangle CAD$ and DI_2 is the bisector of $\measuredangle ADC$. Define point F_1 as the foot of I_1 on line AC. Define point F_2 as the foot of I_2 on line AC. Define points Q, T such that $F_1I_1 = I_1Q$, $F_2I_2 = I_2T$, I_1Q is perpendicular to QT and I_2T is perpendicular to QT. Define points P, S such that $F_1I_1 = I_1P$, $F_2I_2 = I_2S$, I_1P is perpendicular to PS and I_2S is perpendicular to PS. Define point K as the intersection of lines PS and QT. Prove that $OK = OX$

Proof:

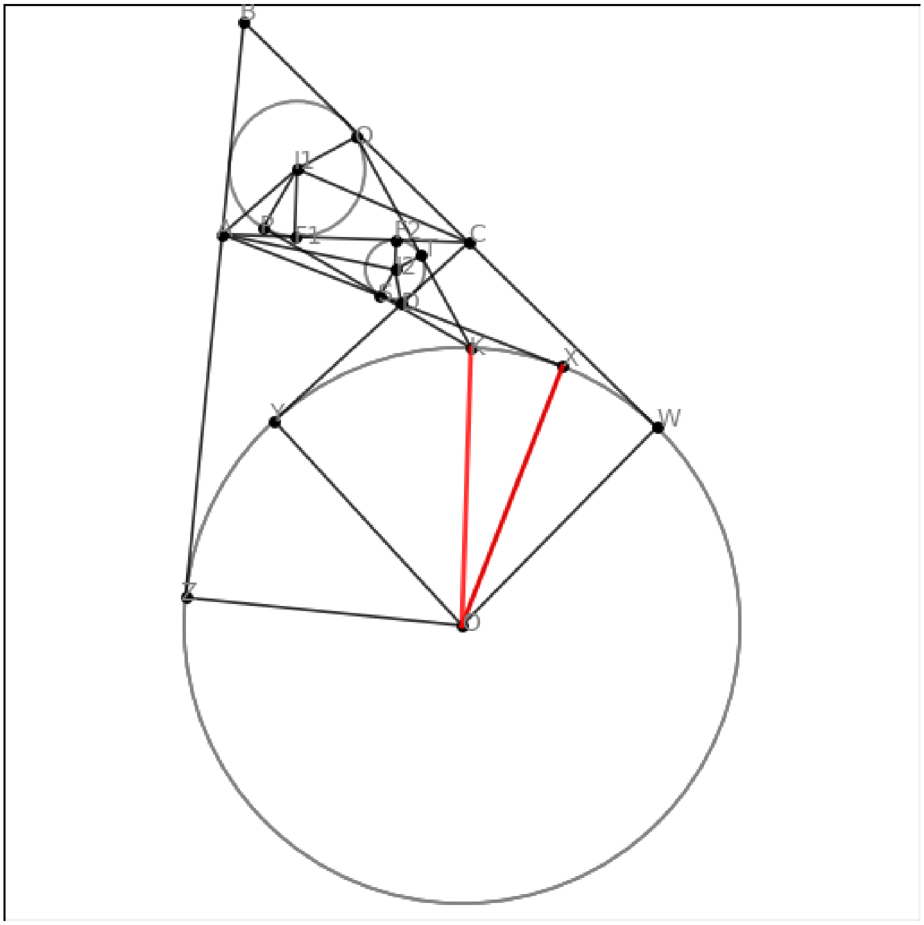

Figure 12: imo 2008 p6

Not solved.

2.13 IMO 2009 P2

Original:

Let ABC be a triangle with circumcenter O. The points P and Q are interior points of the sides CA and AB respectively. Let K, L M be the midpoints of BP, CQ, P Q, respectively, and let Gamma be the circumcircle of triangle KLM. Suppose that P Q is tangent to Gamma. Prove that OP = OQ.

Translated:

Let MLK be a triangle. Define point W as the circumcenter of triangle LMK. Define point Q such that MW is perpendicular to MQ. Define point P as the mirror of Q through M. Define point B as the mirror of P through K. Define point C as the mirror of Q through L. Define point A as the intersection of lines QB and PC. Define point O as the circumcenter of triangle CBA. Prove that $QO = PO$

Proof:

Construct point D such that $MK = LD$ and $MD = LK$.

Construct point E as the mirror of K through W.

Construct point F as the mirror of Q through D.

Step 1. M, Q, P are collinear and $MQ = MP \Rightarrow M$ is the midpoint of QP.

Step 2. L, Q, C are collinear and $LQ = LC \Rightarrow L$ is the midpoint of QC.

Step 3. M is the midpoint of QP and L is the midpoint of $QC \Rightarrow ML$ is parallel to PC.

Step 4. $BO = CO$ and $BO = AO \Rightarrow CO = AO$.

Step 5. $CO = AO \Rightarrow \measuredangle CAO = \measuredangle OCA$.

Step 6. K, P, B are collinear and $KP = KB \Rightarrow K$ is the midpoint of PB.

Step 7. M is the midpoint of QP and K is the midpoint of $PB \Rightarrow MK$ is parallel to QB.

Step 8. $BO = AO \Rightarrow \measuredangle BAO = \measuredangle OBA$.

Step 9. Q, D, F are collinear and $QD = DF \Rightarrow D$ is the midpoint of QF.

Step 10. L is the midpoint of QC and D is the midpoint of $QF \Rightarrow LD$ is parallel to CF.

Step 11. $MK = LD$ and $MD = LK \Rightarrow \measuredangle MLD = \measuredangle KML$ and $\measuredangle MLK = \measuredangle DML$.

Step 12. P, C, A are collinear, $\measuredangle CAO = \measuredangle OCA$ and ML is parallel to PC $\Rightarrow \measuredangle(ML, AO) = \measuredangle(CO, ML)$.

Step 13. Q, B, A are collinear, $\measuredangle BAO = \measuredangle OBA$ and MK is parallel to QB $\Rightarrow \measuredangle(MK, AO) = \measuredangle(BO, MK)$.

Step 14. $\measuredangle MLD = \measuredangle KML$ and LD is parallel to $CF \Rightarrow \measuredangle(ML, CF) = \measuredangle KML$.

Step 15. $\measuredangle(ML, CF) = \measuredangle KML$, $\measuredangle(ML, AO) = \measuredangle(CO, ML)$, $\measuredangle(MK, AO) = \measuredangle(BO, MK)$, MK is parallel to QB and LD is parallel to $CF \Rightarrow$ by angle chasing: $\measuredangle(LD, CO) = \measuredangle QBO$.

Step 16. L, Q, C are collinear, Q, D, F are collinear and LD is parallel to $CF \Rightarrow \frac{LD}{CF} = \frac{QD}{QF}$.

Step 17. M, Q, P are collinear, K, P, B are collinear and MK is parallel to $QB \Rightarrow \frac{MK}{QB} = \frac{PK}{PB}$.

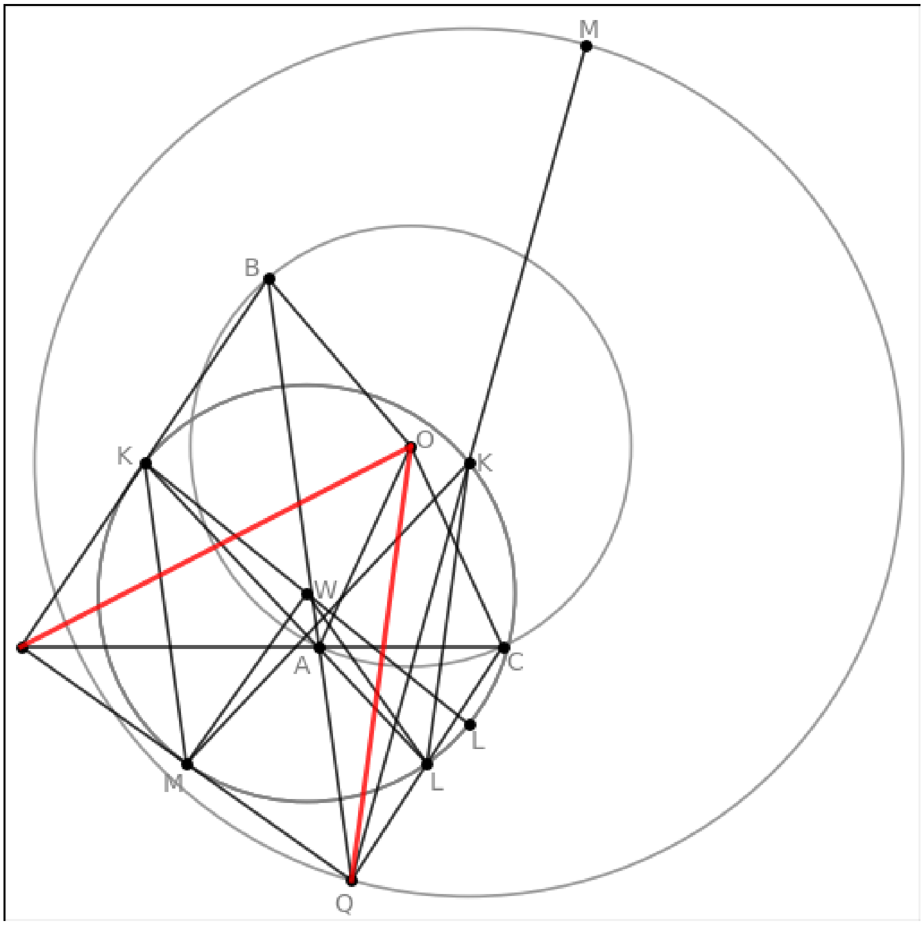

Figure 13: imo 2009 p2

Step 18. K is the midpoint of PB and D is the midpoint of $QF \Rightarrow \frac{BK}{BP} = \frac{FD}{FQ}$.

Step 19. $MK = LD$, $KP = KB$, $QD = DF$, $\frac{MK}{QB} = \frac{PK}{PB}$, $\frac{LD}{CF} = \frac{QD}{QF}$ and $\frac{BK}{BP} = \frac{FD}{FQ} \Rightarrow \frac{MK}{CF} = \frac{LD}{QB}$.

Step 20. $MK = LD$ and $\frac{MK}{CF} = \frac{LD}{QB} \Rightarrow QB = CF$.

Step 21. $\measuredangle(LD, CO) = \measuredangle QBO$ and LD is parallel to $CF \Rightarrow \measuredangle QBO = \measuredangle FCO$.

Step 22. $QB = CF$, $BO = CO$ and $\measuredangle QBO = \measuredangle FCO \Rightarrow \measuredangle(QB, CF) = \measuredangle QOF$ and $QO = OF$.

Step 23. $MW = LW$, $LW = KW$ and $KW = WE \Rightarrow M, L, K, E$ are cyclic.

Step 24. M, L, K, E are cyclic $\Rightarrow \measuredangle MLE = \measuredangle MKE$.

Step 25. $LW = KW$ and $KW = WE \Rightarrow LW = WE$.

Step 26. $LW = WE \Rightarrow \measuredangle WLE = \measuredangle LEW$.

Step 27. K, W, E are collinear, P, C, A are collinear, $\measuredangle(ML, CF) = \measuredangle KML$, $\measuredangle MLE = \measuredangle MKE$, $\measuredangle WLE = \measuredangle LEW$, ML is parallel to PC and LD is parallel to $CF \Rightarrow \measuredangle WLE = \measuredangle(LD, CA)$.

Step 28. K, W, E are collinear, Q, B, A are collinear, P, C, A are collinear, $\measuredangle MLE = \measuredangle MKE$, ML is parallel to PC and MK is parallel to $QB \Rightarrow \measuredangle LEW = \measuredangle CAB$.

Step 29. $\measuredangle WLE = \measuredangle(LD, CA)$ and $\measuredangle LEW = \measuredangle CAB \Rightarrow \measuredangle LWE = \measuredangle(LD, BA)$.

Step 30. K, W, E are collinear, Q, B, A are collinear, $\measuredangle LWE = \measuredangle(LD, BA)$, $\measuredangle(QB, CF) = \measuredangle QOF$ and LD is parallel to $CF \Rightarrow \measuredangle LWK = \measuredangle FOQ$.

Step 31. $LW = KW$ and $QO = OF \Rightarrow \frac{WL}{WK} = \frac{OF}{OQ}$.

Step 32. $\measuredangle LWK = \measuredangle FOQ$ and $\frac{WL}{WK} = \frac{OF}{OQ} \Rightarrow \measuredangle LKW = \measuredangle FQO$.

Step 33. $QO = OF$ and $QD = DF \Rightarrow QF$ is perpendicular to OD.

Step 34. $LW = KW$ and $KW = WE \Rightarrow W$ is the circumcenter of LKE.

Step 35. W is the circumcenter of LKE and K, W, E are collinear $\Rightarrow LK$ is perpendicular to LE.

Step 36. K, W, E are collinear, Q, D, F are collinear and $\measuredangle LKW = \measuredangle FQO$ $\Rightarrow \measuredangle(LK, WE) = \measuredangle(DF, QO)$.

Step 37. Q, D, F are collinear, LK is perpendicular to LE and QF is perpendicular to $OD \Rightarrow \measuredangle KLE = \measuredangle FDO$.

Step 38. $\measuredangle KLE = \measuredangle FDO$ and $\measuredangle(LK, WE) = \measuredangle(DF, QO) \Rightarrow \measuredangle LEW = \measuredangle DOQ$.

Step 39. M is the midpoint of QP and D is the midpoint of $QF \Rightarrow MD$ is parallel to PF.

Step 40. $MW = LW$ and $LW = KW \Rightarrow W$ is the circumcenter of MLK.

Step 41. W is the circumcenter of MLK and MW is perpendicular to MQ $\Rightarrow \measuredangle MLK = \measuredangle QMK$.

Step 42. Q, B, A are collinear, P, C, A are collinear, $\measuredangle MLK = \measuredangle QMK$, $\measuredangle MLK = \measuredangle DML$, ML is parallel to PC, MK is parallel to QB and MD is parallel to $PF \Rightarrow \measuredangle(MQ, BA) = \measuredangle(PF, CA)$.

Step 43. K, W, E are collinear, Q, B, A are collinear, P, C, A are collinear, $\measuredangle MLE = \measuredangle MKE$, ML is parallel to PC and MK is parallel to $QB \Rightarrow \measuredangle(LE, CA) = \measuredangle(WE, BA)$.

Step 44. $\measuredangle(MQ, BA) = \measuredangle(PF, CA)$ and $\measuredangle(LE, CA) = \measuredangle(WE, BA) \Rightarrow \measuredangle(MQ, WE) = \measuredangle(PF, LE)$.

Step 45. $\measuredangle(MQ, WE) = \measuredangle(PF, LE)$, $\measuredangle LEW = \measuredangle DOQ$ and MD is parallel to $PF \Rightarrow \measuredangle QMD = \measuredangle QOD$.

Step 46. $\measuredangle QMD = \measuredangle QOD \Rightarrow M, Q, O, D$ are cyclic.

Step 47. M, Q, O, D are cyclic $\Rightarrow \measuredangle QMO = \measuredangle QDO$.

Step 48. M, Q, P are collinear, Q, D, F are collinear, $\measuredangle QMO = \measuredangle QDO$ and QF is perpendicular to $OD \Rightarrow MO$ is perpendicular to QP.

Step 49. M is the midpoint of QP and MO is perpendicular to $QP \Rightarrow QO = PO$

■

2.14 IMO 2010 P2

Original:

Let I be the incenter of a triangle ABC and let Gamma be its circumcircle. Let line AI intersect Gamma again at D. Let E be a point on arc BDC and F a point on side BC such that $\measuredangle BAF = \measuredangle CAE < 1/2\measuredangle BAC$. Finally, let G be the midpoint of IF. Prove that DG and EI intersect on Gamma.

Translated:

Let ABC be a triangle. Define point O as the circumcenter of triangle CBA. Define point I such that AI is the bisector of $\measuredangle BAC$ and CI is the bisector of $\measuredangle ACB$. Define point D as the intersection of circle (O, A) and line AI. Let F be any point on line BC. Define point E on circle (O, A) such that $\measuredangle BAF = \measuredangle EAC$. Define point G as the midpoint of IF. Define point K as the intersection of lines IE and DG. Prove that $AO = OK$

Proof:

Construct point H as the mirror of E through O.

Construct point L as the midpoint of AI.

Construct point M as the midpoint of BI.

Step 1. $AO = BO$, $AO = OD$ and $BO = CO \Rightarrow A, B, C, D$ are cyclic.

Step 2. $AO = BO$, $AO = OD$, $AO = OE$ and A, B, C, D are cyclic $\Rightarrow A, B, C, E$ are cyclic.

Step 3. $AO = OD$, $AO = OE$ and $OE = OH \Rightarrow A, D, E, H$ are cyclic.

Step 4. A, B, C, D are cyclic, A, B, C, E are cyclic and A, D, E, H are cyclic $\Rightarrow A, B, D, H$ are cyclic.

Step 5. A, B, D, H are cyclic $\Rightarrow \measuredangle DAH = \measuredangle DBH$ and $\measuredangle ABH = \measuredangle ADH$.

Step 6. A, B, C, D are cyclic, A, B, C, E are cyclic and A, D, E, H are cyclic $\Rightarrow A, C, D, H$ are cyclic.

Step 7. A, C, D, H are cyclic $\Rightarrow \measuredangle DAH = \measuredangle DCH$.

Step 8. A, B, C, D are cyclic, A, B, C, E are cyclic and A, D, E, H are cyclic $\Rightarrow B, C, D, H$ are cyclic.

Step 9. B, C, D, H are cyclic $\Rightarrow \measuredangle BCH = \measuredangle BDH$.

Step 10. A, I, D are collinear and $\measuredangle DAH = \measuredangle DBH \Rightarrow \measuredangle IAH = \measuredangle DBH$.

Step 11. A, I, D are collinear and $\measuredangle DAH = \measuredangle DCH \Rightarrow \measuredangle IAH = \measuredangle DCH$.

Step 12. A, I, D are collinear and $\measuredangle ABH = \measuredangle ADH \Rightarrow \measuredangle ABH = \measuredangle(AI, DH)$.

Step 13. AI is the bisector of $\measuredangle BAC$, $\measuredangle IAH = \measuredangle DBH$, $\measuredangle IAH = \measuredangle DCH$, $\measuredangle ABH = \measuredangle(AI, DH)$, CI is the bisector of $\measuredangle ACB$ and $\measuredangle BCH = \measuredangle BDH \Rightarrow$ by angle chasing: $\measuredangle AIC = \measuredangle ICD$.

Step 14. A, I, D are collinear and $\measuredangle AIC = \measuredangle ICD \Rightarrow \measuredangle CID = \measuredangle DCI$.

Step 15. $\measuredangle CID = \measuredangle DCI \Rightarrow CD = ID$.

Step 16. A, I, D are collinear and AI is the bisector of $\measuredangle BAC \Rightarrow AD$ is the bisector of $\measuredangle BAC$.

Step 17. A, B, C, D are cyclic and AD is the bisector of $\measuredangle BAC \Rightarrow BD = CD$.

Step 18. $BD = CD$ and $CD = ID \Rightarrow D$ is the circumcenter of BCI.

Step 19. B, I, M are collinear and $BM = IM \Rightarrow M$ is the midpoint of BI.

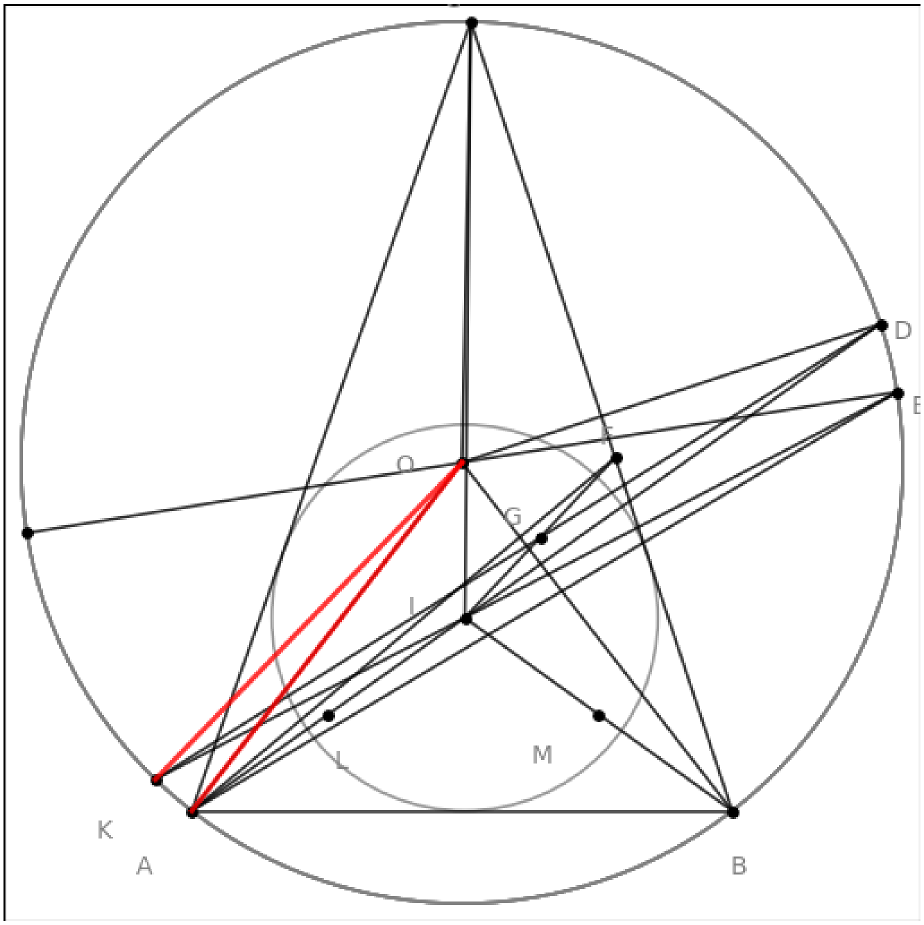

Figure 14: imo 2010 p2

Step 20. D is the circumcenter of BCI and M is the midpoint of $BI \Rightarrow \measuredangle BCI = \measuredangle MDI$.

Step 21. A, I, L are collinear and $AL = IL \Rightarrow L$ is the midpoint of AI.

Step 22. L is the midpoint of AI and M is the midpoint of $BI \Rightarrow AB$ is parallel to LM.

Step 23. A, I, D are collinear, A, I, L are collinear, CI is the bisector of $\measuredangle ACB$ and $\measuredangle BCI = \measuredangle MDI \Rightarrow \measuredangle ACI = \measuredangle LDM$.

Step 24. A, I, D are collinear, A, I, L are collinear, AI is the bisector of $\measuredangle BAC$ and AB is parallel to $LM \Rightarrow \measuredangle CAI = \measuredangle DLM$.

Step 25. $\measuredangle CAI = \measuredangle DLM$ and $\measuredangle ACI = \measuredangle LDM \Rightarrow \frac{IA}{IC} = \frac{ML}{MD}$ and $\frac{CA}{CI} = \frac{DL}{DM}$.

Step 26. A, B, C, E are cyclic $\Rightarrow \measuredangle ABC = \measuredangle AEC$.

Step 27. B, C, F are collinear and $\measuredangle ABC = \measuredangle AEC \Rightarrow \measuredangle ABF = \measuredangle AEC$.

Step 28. $\angle BAF = \angle EAC$ and $\angle ABF = \angle AEC \Rightarrow \frac{AB}{AF} = \frac{AE}{AC}$.

Step 29. A, I, L are collinear, B, I, M are collinear and AB is parallel to $LM \Rightarrow \frac{AB}{LM} = \frac{IA}{IL}$.

Step 30. I, F, G are collinear and $IG = FG \Rightarrow G$ is the midpoint of IF.

Step 31. G is the midpoint of IF and L is the midpoint of $AI \Rightarrow AF$ is parallel to GL.

Step 32. A, I, L are collinear, I, F, G are collinear and AF is parallel to GL $\Rightarrow \frac{AF}{GL} = \frac{IF}{IG}$.

Step 33. O, E, H are collinear and $OE = OH \Rightarrow O$ is the midpoint of EH.

Step 34. O is the midpoint of EH and L is the midpoint of $AI \Rightarrow \frac{IA}{IL} = \frac{HE}{HO}$.

Step 35. O is the midpoint of EH and G is the midpoint of $IF \Rightarrow \frac{HO}{HE} = \frac{IG}{IF}$.

Step 36. $AL = IL$ and $\frac{AB}{LM} = \frac{IA}{IL} \Rightarrow \frac{AB}{LM} = \frac{AI}{AL}$.

Step 37. $IG = FG$ and $\frac{AF}{GL} = \frac{IF}{IG} \Rightarrow \frac{AF}{GL} = \frac{FI}{FG}$.

Step 38. $AL = IL$, $OE = OH$ and $\frac{IA}{IL} = \frac{HE}{HO} \Rightarrow \frac{AL}{AI} = \frac{EO}{EH}$.

Step 39. $OE = OH$, $IG = FG$ and $\frac{HO}{HE} = \frac{IG}{IF} \Rightarrow \frac{EO}{EH} = \frac{FG}{FI}$.

Step 40. $\frac{AB}{AF} = \frac{AE}{AC}$, $\frac{AB}{LM} = \frac{AI}{AL}$, $\frac{AF}{GL} = \frac{FI}{FG}$, $\frac{AL}{AI} = \frac{EO}{EH}$, $\frac{CA}{CI} = \frac{DL}{DM}$, $\frac{IA}{IC} = \frac{ML}{MD}$ and $\frac{EO}{EH} = \frac{FG}{FI} \Rightarrow$ by ratio chasing: $\frac{AI}{AE} = \frac{LG}{LD}$.

Step 41. AI is the bisector of $\angle BAC$ and $\angle BAF = \angle EAC \Rightarrow$ by angle chasing: AI is the bisector of $\angle EAF$.

Step 42. A, I, D are collinear, A, I, L are collinear, AI is the bisector of $\angle EAF$ and AF is parallel to $GL \Rightarrow \angle IAE = \angle GLD$.

Step 43. $\angle IAE = \angle GLD$ and $\frac{AI}{AE} = \frac{LG}{LD} \Rightarrow \angle AEI = \angle LDG$.

Step 44. A, D, E, H are cyclic $\Rightarrow \angle ADH = \angle AEH$.

Step 45. A, I, D are collinear, A, I, L are collinear, I, E, K are collinear, D, G, K are collinear, $\angle ADH = \angle AEH$ and $\angle AEI = \angle LDG \Rightarrow \angle DKE = \angle DHE$.

Step 46. $\angle DKE = \angle DHE \Rightarrow D, E, K, H$ are cyclic.

Step 47. A, C, D, H are cyclic, A, D, E, H are cyclic and D, E, K, H are cyclic $\Rightarrow C, E, K, H$ are cyclic.

Step 48. C, E, K, H are cyclic $\Rightarrow \angle ECH = \angle EKH$.

Step 49. D, E, K, H are cyclic $\Rightarrow \angle EDH = \angle EKH$.

Step 50. $AO = BO$, $AO = OE$, $BO = CO$ and $OE = OH \Rightarrow O$ is the circumcenter of CEH.

Step 51. O is the circumcenter of CEH and O, E, H are collinear $\Rightarrow CE$ is perpendicular to CH.

Step 52. $AO = OD$, $AO = OE$ and $OE = OH \Rightarrow O$ is the circumcenter of DEH.

Step 53. O is the circumcenter of DEH and O, E, H are collinear $\Rightarrow DE$ is perpendicular to DH.

Step 54. CE is perpendicular to CH and DE is perpendicular to $DH \Rightarrow \angle ECH = \angle HDE$.

Step 55. $\angle ECH = \angle HDE$, $\angle ECH = \angle EKH$ and $\angle EDH = \angle EKH \Rightarrow EK$ is perpendicular to KH.

Step 56. O is the midpoint of EH and EK is perpendicular to $KH \Rightarrow OE = OK$.

Step 57. $AO = OE$ and $OE = OK \Rightarrow AO = OK$
■

2.15 IMO 2010 P4

Original:

Let P be a point interior to triangle ABC (with $CA \neq CB$). The lines AP, BP and CP meet again its circumcircle Gamma at K, L, M, respectively. The tangent line at C to Gamma meets the line AB at S. Show that from SC = SP follows MK = ML.

Translated:

Let S and C be any two distinct points. Define point P on circle (S, C) such that $\measuredangle CPS = \measuredangle SCP$. Define point O such that CO is perpendicular to CS. Let A be any point on circle (O, C). Define point B as the intersection of circle (O, C) and line AS. Define point M as the intersection of circle (O, C) and line CP. Define point L as the intersection of circle (O, C) and line BP. Define point K as the intersection of circle (O, C) and line AP. Prove that $KM = LM$

Proof:

Step 1. $AO = CO$ and $BO = CO \Rightarrow O$ is the circumcenter of ABC.

Step 2. O is the circumcenter of ABC and CO is perpendicular to $CS \Rightarrow \measuredangle BAC = \measuredangle BCS$ and $\measuredangle ABC = \measuredangle ACS$.

Step 3. A, B, S are collinear and $\measuredangle BAC = \measuredangle BCS \Rightarrow \measuredangle SAC = \measuredangle BCS$.

Step 4. A, B, S are collinear and $\measuredangle ABC = \measuredangle ACS \Rightarrow \measuredangle ACS = \measuredangle SBC$.

Step 5. $\measuredangle ACS = \measuredangle SBC$ and $\measuredangle SAC = \measuredangle BCS \Rightarrow \frac{SA}{SC} = \frac{SC}{SB}$.

Step 6. $CS = PS$ and $\frac{SA}{SC} = \frac{SC}{SB} \Rightarrow \frac{SA}{SP} = \frac{SP}{SB}$.

Step 7. A, B, S are collinear $\Rightarrow \measuredangle ASP = \measuredangle BSP$.

Step 8. $\measuredangle ASP = \measuredangle BSP$ and $\frac{SA}{SP} = \frac{SP}{SB} \Rightarrow \measuredangle SAP = \measuredangle BPS$.

Step 9. $AO = CO$, $BO = CO$, $CO = LO$ and $CO = MO \Rightarrow A, B, L, M$ are cyclic.

Step 10. A, B, L, M are cyclic $\Rightarrow \measuredangle ABL = \measuredangle AML$.

Step 11. $CO = KO$, $CO = LO$ and $CO = MO \Rightarrow C, K, L, M$ are cyclic.

Step 12. C, K, L, M are cyclic $\Rightarrow \measuredangle CKL = \measuredangle CML$.

Step 13. $CO = KO$ and $CO = MO \Rightarrow O$ is the circumcenter of CKM.

Step 14. O is the circumcenter of CKM and CO is perpendicular to $CS \Rightarrow \measuredangle CKM = \measuredangle SCM$.

Step 15. C, M, P are collinear and $\measuredangle CKL = \measuredangle CML \Rightarrow \measuredangle CKL = \measuredangle(CP, LM)$.

Step 16. C, M, P are collinear, $\measuredangle CKM = \measuredangle SCM$ and $\measuredangle CPS = \measuredangle SCP \Rightarrow \measuredangle CKM = \measuredangle CPS$.

Step 17. $\measuredangle CKL = \measuredangle(CP, LM)$ and $\measuredangle CKM = \measuredangle CPS \Rightarrow \measuredangle KLM = \measuredangle(KM, PS)$.

Step 18. A, B, S are collinear, B, L, P are collinear, $\measuredangle SAP = \measuredangle BPS$ and $\measuredangle ABL = \measuredangle AML \Rightarrow \measuredangle AML = \measuredangle APS$.

Step 19. $\measuredangle KLM = \measuredangle(KM, PS)$ and $\measuredangle AML = \measuredangle APS \Rightarrow \measuredangle(AM, KL) = \measuredangle(AP, KM)$.

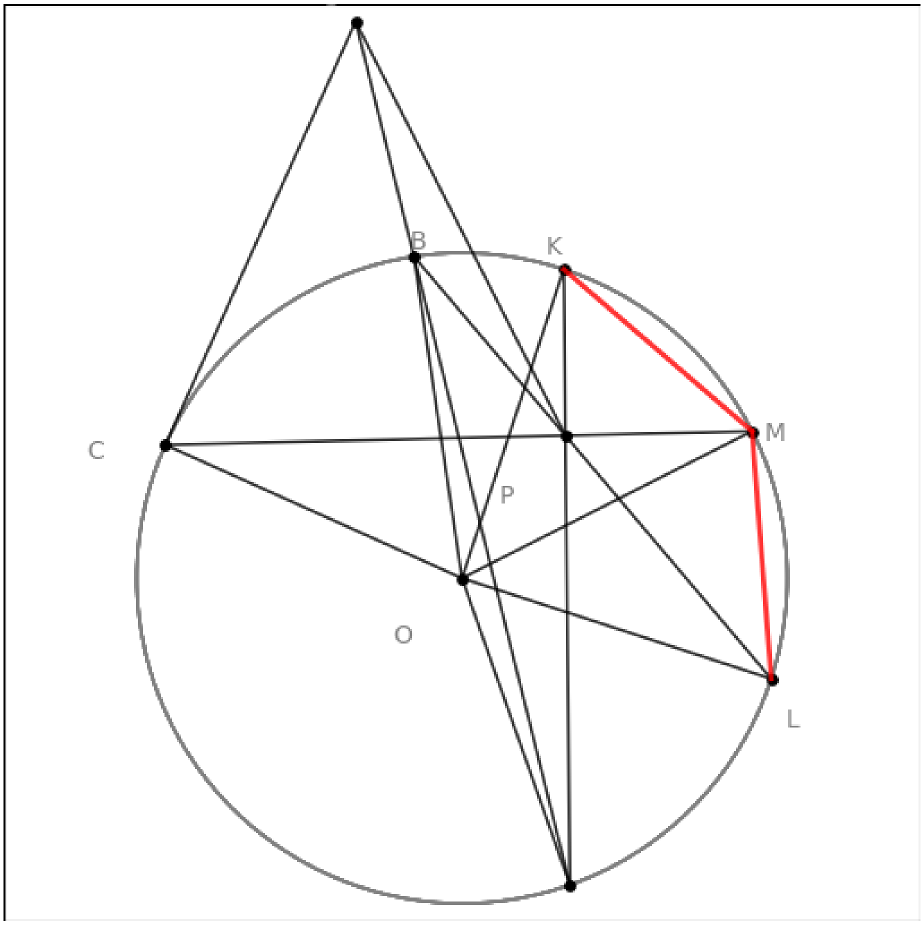

Figure 15: imo 2010 p4

Step 20. $AO = CO$, $BO = CO$, $CO = LO$, $CO = MO$ and C, K, L, M are cyclic $\Rightarrow$ A, B, K, M are cyclic.

Step 21. A, B, K, M are cyclic $\Rightarrow \measuredangle KAM = \measuredangle KBM$.

Step 22. A, K, P are collinear, $\measuredangle KAM = \measuredangle KBM$ and $\measuredangle(AM, KL) = \measuredangle(AP, KM) \Rightarrow \measuredangle KBM = \measuredangle MKL$.

Step 23. $BO = CO$, $CO = LO$, $CO = MO$ and C, K, L, M are cyclic $\Rightarrow$ B, K, L, M are cyclic.

Step 24. B, K, L, M are cyclic and $\measuredangle KBM = \measuredangle MKL \Rightarrow KM = LM$

■

2.16 IMO 2011 P6

Original:

Let ABC be a triangle with circumcircle Gamma. Let l be a tangent line to Gamma, and let la, lb, lc be the lines obtained by reflecting l in the lines BC, CA, and AB, respectively. Show that the circumcircle of the triangle determined by the lines la, lb, and lc is tangent to the circle Gamma.

Translated:

Let ABC be a triangle. Define point O as the circumcenter of triangle CBA. Let P be any point on circle (O, A). Define point Q such that OP is perpendicular to PQ. Define point P_A as the intersection of circles (B, P) and (C, P). Define point P_B as the intersection of circles (A, P) and (C, P). Define point P_C as the intersection of circles (A, P) and (B, P). Define point Q_A as the intersection of circles (B, Q) and (C, Q). Define point Q_B as the intersection of circles (A, Q) and (C, Q). Define point Q_C as the intersection of circles (A, Q) and (B, Q). Define point A_1 as the intersection of lines P_BQ_B and P_CQ_C. Define point B_1 as the intersection of lines P_AQ_A and P_CQ_C. Define point C_1 as the intersection of lines P_AQ_A and P_BQ_B. Define point O_1 as the circumcenter of triangle $B_1A_1C_1$. Define point X as the intersection of circles (O, A) and (O_1, A_1). Prove that X, O, O_1 are collinear

Proof:

Not solved.

2.17 IMO 2012 P1

Original:

Given triangle ABC the point J is the centre of the excircle opposite the vertex A. This excircle is tangent to the side BC at M, and to the lines AB and AC at K and L, respectively. The lines LM and BJ meet at F, and the lines KM and CJ meet at G. Let S be the point of intersection of the lines AF and BC, and let T be the point of intersection of the lines AG and BC. Prove that M is the midpoint of ST.

Translated:

Let ABC be a triangle. Define point J such that AJ is the bisector of $\measuredangle BAC$ and CJ is the bisector of $\measuredangle ACB$. Define point M as the foot of J on line BC. Define point L as the foot of J on line AC. Define point K as the foot of J on line AB. Define point F as the intersection of lines BJ and LM. Define point G as the intersection of lines CJ and KM. Define point S as the intersection of lines AF and BC. Define point T as the intersection of lines AG and BC. Prove that $MS = MT$

Proof:

Step 1. A, C, L are collinear, B, C, M are collinear and CJ is the bisector of $\measuredangle ACB \Rightarrow CJ$ is the bisector of $\measuredangle MCL$.

Step 2. A, C, L are collinear, B, C, M are collinear, AC is perpendicular to JL and BC is perpendicular to $JM \Rightarrow \measuredangle CMJ = \measuredangle JLC$.

Step 3. CJ is the bisector of $\measuredangle MCL$ and $\measuredangle CMJ = \measuredangle JLC \Rightarrow JL = JM$, $CL = CM$ and JC is the bisector of $\measuredangle MJL$.

Step 4. A, B, K are collinear, A, C, L are collinear, AB is perpendicular to JK and AC is perpendicular to $JL \Rightarrow \measuredangle AKJ = \measuredangle JLA$.

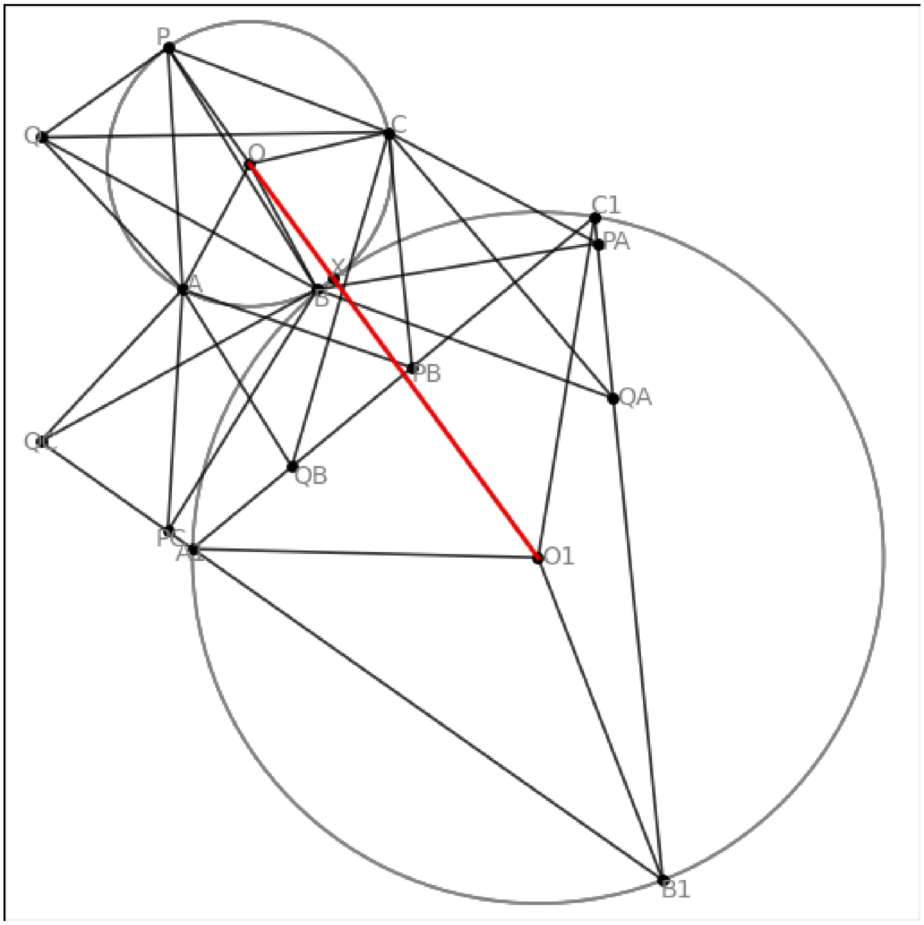

Figure 16: imo 2011 p6

Step 5. A, B, K are collinear, A, C, L are collinear and AJ is the bisector of $\measuredangle BAC \Rightarrow AJ$ is the bisector of $\measuredangle LAK$.

Step 6. AJ is the bisector of $\measuredangle LAK$ and $\measuredangle AKJ = \measuredangle JLA \Rightarrow JK = JL$.

Step 7. $JK = JL$ and $JL = JM \Rightarrow J$ is the circumcenter of KLM.

Step 8. B, C, M are collinear, B, C, S are collinear and BC is perpendicular to $JM \Rightarrow JM$ is perpendicular to MS.

Step 9. J is the circumcenter of KLM and JM is perpendicular to $MS \Rightarrow \measuredangle LKM = \measuredangle LMS$.

Step 10. A, B, K are collinear and AB is perpendicular to $JK \Rightarrow AK$ is perpendicular to JK.

Step 11. J is the circumcenter of KLM and AK is perpendicular to $JK \Rightarrow \measuredangle AKL = \measuredangle KML$.

Step 12. A, B, K are collinear, B, C, M are collinear, AB is perpendicular

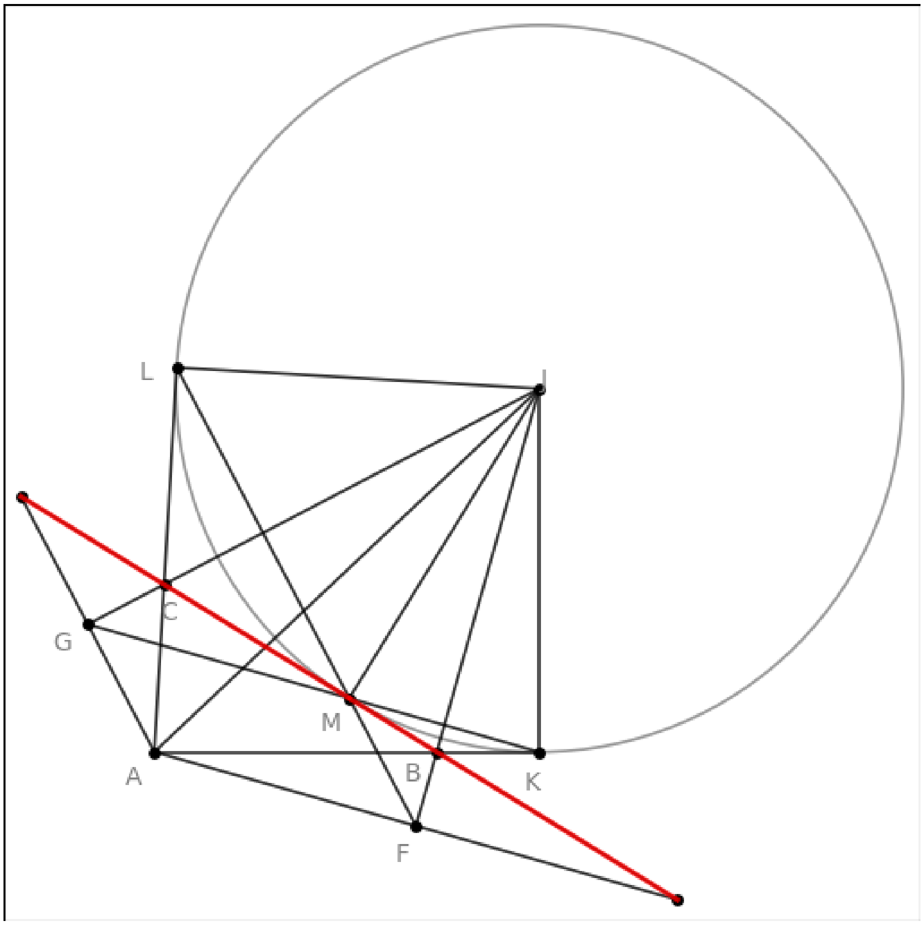

Figure 17: imo 2012 p1

to JK and BC is perpendicular to $JM \Rightarrow \measuredangle BKJ = \measuredangle BMJ$.

Step 13. $\measuredangle BKJ = \measuredangle BMJ \Rightarrow B, J, K, M$ are cyclic.

Step 14. B, J, K, M are cyclic $\Rightarrow \measuredangle JBM = \measuredangle JKM$ and $\measuredangle JBK = \measuredangle JMK$.

Step 15. $JK = JL$ and $JL = JM \Rightarrow JK = JM$.

Step 16. A, B, K are collinear, B, C, M are collinear, B, C, S are collinear, B, F, J are collinear, $\measuredangle JBK = \measuredangle JMK$, $\measuredangle JBM = \measuredangle JKM$, $\measuredangle AKL = \measuredangle KML$ and $\measuredangle LKM = \measuredangle LMS \Rightarrow JF$ is the bisector of $\measuredangle MJK$.

Step 17. $JK = JM$ and JF is the bisector of $\measuredangle MJK \Rightarrow \measuredangle FKJ = \measuredangle JMF$.

Step 18. $CL = CM$ and $JL = JM \Rightarrow CJ$ is perpendicular to LM.

Step 19. F, L, M are collinear and CJ is perpendicular to $LM \Rightarrow CJ$ is perpendicular to FM.

Step 20. AK is perpendicular to JK and CJ is perpendicular to $FM \Rightarrow \measuredangle(AK, CJ) = \measuredangle(JK, FM)$.

Step 21. $\measuredangle(AK, CJ) = \measuredangle(JK, FM)$ and $\measuredangle FKJ = \measuredangle JMF \Rightarrow \measuredangle(AK, CJ) = \measuredangle(FK, JM)$.

Step 22. B, C, S are collinear, F, L, M are collinear, BC is perpendicular to JM and CJ is perpendicular to $LM \Rightarrow \measuredangle(BS, JM) = \measuredangle(FM, CJ)$.

Step 23. $\measuredangle(AK, CJ) = \measuredangle(FK, JM)$ and $\measuredangle(BS, JM) = \measuredangle(FM, CJ) \Rightarrow \measuredangle(AK, FM) = \measuredangle(FK, BS)$.

Step 24. A, C, L are collinear, B, C, M are collinear, AC is perpendicular to JL and BC is perpendicular to $JM \Rightarrow \measuredangle CLJ = \measuredangle CMJ$.

Step 25. $\measuredangle CLJ = \measuredangle CMJ \Rightarrow C, J, L, M$ are cyclic.

Step 26. C, J, L, M are cyclic $\Rightarrow \measuredangle JCM = \measuredangle JLM$.

Step 27. B, C, M are collinear, B, C, S are collinear, C, G, J are collinear, G, K, M are collinear, $\measuredangle JCM = \measuredangle JLM$ and $\measuredangle LKM = \measuredangle LMS \Rightarrow \measuredangle GJL = \measuredangle GKL$.

Step 28. $\measuredangle GJL = \measuredangle GKL \Rightarrow G, J, K, L$ are cyclic.

Step 29. A, B, K are collinear, A, C, L are collinear, AB is perpendicular to JK and AC is perpendicular to $JL \Rightarrow \measuredangle AKJ = \measuredangle ALJ$.

Step 30. $\measuredangle AKJ = \measuredangle ALJ \Rightarrow A, J, K, L$ are cyclic.

Step 31. A, J, K, L are cyclic and G, J, K, L are cyclic $\Rightarrow A, G, J, K$ are cyclic.

Step 32. A, G, J, K are cyclic $\Rightarrow \measuredangle AGJ = \measuredangle AKJ$.

Step 33. B, C, M are collinear, B, C, S are collinear, B, F, J are collinear, F, L, M are collinear, $\measuredangle JBM = \measuredangle JKM$ and $\measuredangle LKM = \measuredangle LMS \Rightarrow \measuredangle FJK = \measuredangle FLK$.

Step 34. $\measuredangle FJK = \measuredangle FLK \Rightarrow F, J, K, L$ are cyclic.

Step 35. A, J, K, L are cyclic and F, J, K, L are cyclic $\Rightarrow A, F, J, K$ are cyclic.

Step 36. A, F, J, K are cyclic and F, J, K, L are cyclic $\Rightarrow A, F, J, L$ are cyclic.

Step 37. A, F, J, L are cyclic $\Rightarrow \measuredangle AFJ = \measuredangle ALJ$.

Step 38. A, F, J, K are cyclic $\Rightarrow \measuredangle AFJ = \measuredangle AKJ$.

Step 39. A, B, K are collinear, B, C, M are collinear, B, C, S are collinear, $\measuredangle JBK = \measuredangle JMK$, $\measuredangle JBM = \measuredangle JKM$, $\measuredangle AKL = \measuredangle KML$ and $\measuredangle LKM = \measuredangle LMS \Rightarrow JB$ is the bisector of $\measuredangle MJK$.

Step 40. B, J, K, M are cyclic and JB is the bisector of $\measuredangle MJK \Rightarrow BK = BM$.

Step 41. $BK = BM$ and $JK = JM \Rightarrow BJ$ is perpendicular to KM.

Step 42. $BK = BM \Rightarrow \measuredangle BKM = \measuredangle KMB$.

Step 43. A, B, K are collinear, A, G, T are collinear, B, C, M are collinear, B, C, S are collinear, B, C, T are collinear, C, G, J are collinear, F, L, M are collinear, $\measuredangle(AK, FM) = \measuredangle(FK, BS)$, $\measuredangle AGJ = \measuredangle AKJ$, AB is perpendicular to JK and CJ is perpendicular to $LM \Rightarrow \measuredangle AKF = \measuredangle GTM$.

Step 44. A, B, K are collinear, B, C, M are collinear, B, C, T are collinear, B, F, J are collinear, G, K, M are collinear, $\measuredangle AFJ = \measuredangle AKJ$, $\measuredangle BKM = \measuredangle KMB$, AB is perpendicular to JK and BJ is perpendicular to $KM \Rightarrow \measuredangle FAK = \measuredangle TMG$.

Step 45. $\measuredangle FAK = \measuredangle TMG$ and $\measuredangle AKF = \measuredangle GTM \Rightarrow \frac{AF}{GM} = \frac{AK}{MT}$.

Step 46. $JK = JM \Rightarrow \measuredangle JKM = \measuredangle KMJ$.

Step 47. A, J, K, L are cyclic $\Rightarrow \measuredangle JAL = \measuredangle JKL$.

Step 48. A, B, K are collinear, B, F, J are collinear, $\measuredangle JBK = \measuredangle JMK$ and $\measuredangle JKM = \measuredangle KMJ \Rightarrow \measuredangle(AK, FJ) = \measuredangle JKM$.

Step 49. A, B, K are collinear, A, C, L are collinear, AJ is the bisector of $\measuredangle BAC$ and $\measuredangle JAL = \measuredangle JKL \Rightarrow \measuredangle KAJ = \measuredangle JKL$.

Step 50. $\measuredangle KAJ = \measuredangle JKL$ and $\measuredangle(AK, FJ) = \measuredangle JKM \Rightarrow \measuredangle AJF = \measuredangle LKM$.

Step 51. B, C, M are collinear, B, C, S are collinear, C, G, J are collinear, $\measuredangle JCM = \measuredangle JLM$, $\measuredangle AJF = \measuredangle LKM$ and $\measuredangle LKM = \measuredangle LMS \Rightarrow \measuredangle AJF = \measuredangle LJG$.

Step 52. A, F, J, K are cyclic, A, G, J, K are cyclic and G, J, K, L are cyclic $\Rightarrow A, F, G, J, L$ are cyclic.

Step 53. A, F, G, J, L are cyclic and $\measuredangle AJF = \measuredangle LJG \Rightarrow AF = GL$.

Step 54. C, G, J are collinear and JC is the bisector of $\measuredangle MJL \Rightarrow JG$ is the bisector of $\measuredangle MJL$.

Step 55. $JL = JM$ and JG is the bisector of $\measuredangle MJL \Rightarrow GL = GM$.

Step 56. B, C, M are collinear and B, C, S are collinear $\Rightarrow B, M, S$ are collinear.

Step 57. A, C, L are collinear, A, F, S are collinear, B, F, J are collinear, $\measuredangle AFJ = \measuredangle ALJ$, AC is perpendicular to JL and BJ is perpendicular to KM $\Rightarrow AS$ is parallel to KM.

Step 58. A, B, K are collinear, B, M, S are collinear and AS is parallel to $KM \Rightarrow \frac{AK}{MS} = \frac{BK}{BM}$.

Step 59. $AF = GL$, $BK = BM$, $GL = GM$, $\frac{AF}{GM} = \frac{AK}{MT}$ and $\frac{AK}{MS} = \frac{BK}{BM} \Rightarrow MS = MT$

■

2.18 IMO 2012 P5

Original:

Let ABC be a triangle with $\measuredangle BCA = 90^o$, and let D be the foot of the altitude from C. Let X be a point in the interior of the segment CD. Let K be the point on the segment AX such that BK = BC. Similarly, let L be the point on the segment BX such that AL = AC. Let $M = AL \cap BK$. Prove that MK = ML

Translated:

Let C and A be any two distinct points. Define point B such that CA is perpendicular to CB. Define point D as the foot of C on line AB. Let X be any point on line CD. Define point K as the intersection of circle (B, C) and line AX. Define point L as the intersection of circle (A, C) and line BX. Define point M as the intersection of lines AL and BK. Prove that $KM = LM$

Proof:

Construct point J such that $AJ = BJ$ and $\measuredangle CAJ = \measuredangle JCA$.

Construct point E such that AJ is perpendicular to DE and AE is perpendicular to BX.

Step 1. $\measuredangle CAJ = \measuredangle JCA \Rightarrow CJ = AJ$.

Step 2. $CJ = AJ$ and $AJ = BJ \Rightarrow CJ = BJ$.

Step 3. $CJ = BJ \Rightarrow \measuredangle CBJ = \measuredangle JCB$.

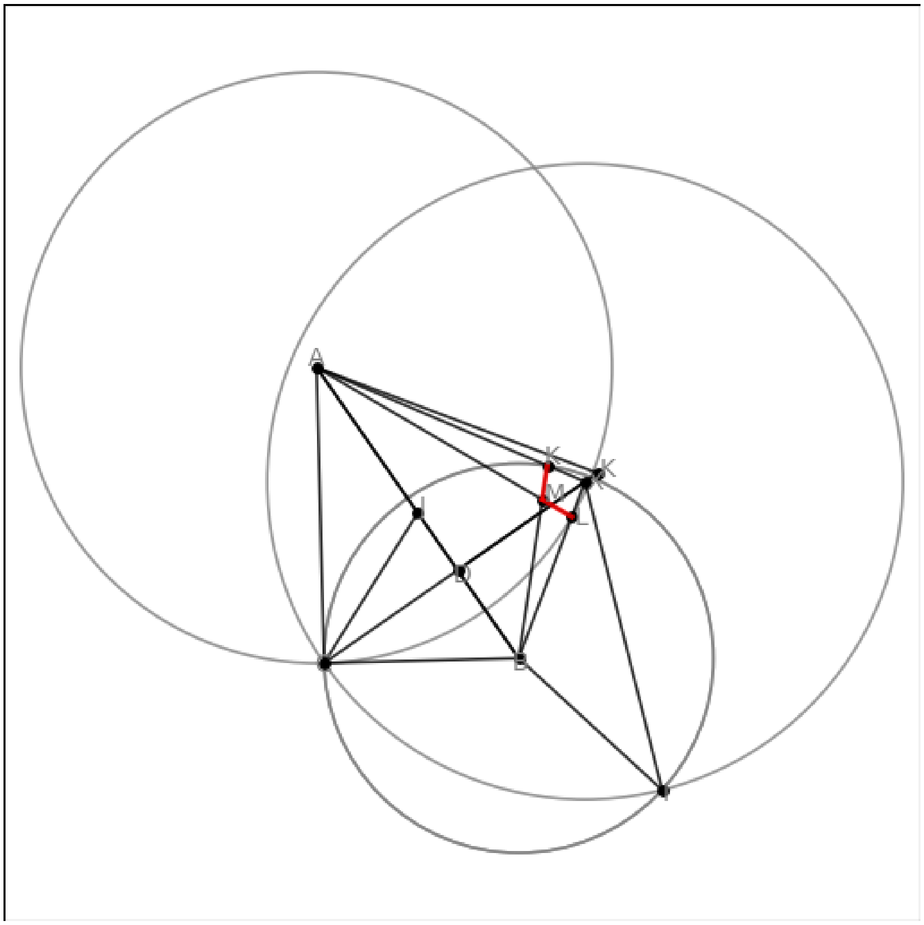

Figure 18: imo 2012 p5

Step 4. CA is perpendicular to CB and AJ is perpendicular to $DE \Rightarrow \measuredangle ACB = \measuredangle(AJ, DE)$.

Step 5. $\measuredangle ACB = \measuredangle(AJ, DE)$ and $\measuredangle CAJ = \measuredangle JCA \Rightarrow \measuredangle(CA, DE) = \measuredangle JCB$.

Step 6. $\measuredangle(CA, DE) = \measuredangle JCB$ and $\measuredangle CBJ = \measuredangle JCB \Rightarrow \measuredangle(CA, DE) = \measuredangle CBJ$.

Step 7. $\measuredangle(CA, DE) = \measuredangle CBJ$ and CA is perpendicular to $CB \Rightarrow BJ$ is perpendicular to DE.

Step 8. C, D, X are collinear, A, B, D are collinear and CD is perpendicular to $AB \Rightarrow AD$ is perpendicular to DX.

Step 9. AD is perpendicular to DX and AJ is perpendicular to $DE \Rightarrow \measuredangle ADE = \measuredangle(DX, AJ)$ and $\measuredangle DAJ = \measuredangle XDE$.

Step 10. B, X, L are collinear and AE is perpendicular to $BX \Rightarrow AE$ is

perpendicular to XL.

Step 11. AD is perpendicular to DX and AE is perpendicular to $XL \Rightarrow \measuredangle DAE = \measuredangle DXL$ and $\measuredangle(AD, XL) = \measuredangle(DX, AE)$.

Step 12. A, B, D are collinear, $\measuredangle ADE = \measuredangle(DX, AJ)$, AJ is perpendicular to DE and BJ is perpendicular to $DE \Rightarrow \measuredangle ADE = \measuredangle XDB$.

Step 13. B, X, L are collinear and $\measuredangle DAE = \measuredangle DXL \Rightarrow \measuredangle EAD = \measuredangle BXD$.

Step 14. $\measuredangle EAD = \measuredangle BXD$ and $\measuredangle ADE = \measuredangle XDB \Rightarrow \frac{DA}{DX} = \frac{DE}{DB}$.

Step 15. A, B, D are collinear, $\measuredangle ADE = \measuredangle(DX, AJ)$, AJ is perpendicular to DE and BJ is perpendicular to $DE \Rightarrow \measuredangle ADX = \measuredangle EDB$.

Step 16. $\measuredangle ADX = \measuredangle EDB$ and $\frac{DA}{DX} = \frac{DE}{DB} \Rightarrow \measuredangle XAD = \measuredangle BED$ and $\measuredangle AXD = \measuredangle EBD$.

Step 17. A, B, D are collinear, $\measuredangle DAJ = \measuredangle XDE$, AJ is perpendicular to DE and BJ is perpendicular to $DE \Rightarrow \measuredangle ADX = \measuredangle ADE$.

Step 18. $\measuredangle ADX = \measuredangle ADE \Rightarrow DX$ is parallel to DE.

Step 19. DX is parallel to $DE \Rightarrow D, X, E$ are collinear.

Step 20. A, B, D are collinear, CA is perpendicular to CB and CD is perpendicular to $AB \Rightarrow \measuredangle BCA = \measuredangle CDB$.

Step 21. A, B, D are collinear $\Rightarrow \measuredangle CBA = \measuredangle CBD$.

Step 22. $\measuredangle BCA = \measuredangle CDB$ and $\measuredangle CBA = \measuredangle CBD \Rightarrow \frac{BC}{BD} = \frac{BA}{BC}$.

Step 23. A, B, D are collinear $\Rightarrow \measuredangle ABK = \measuredangle DBK$.

Step 24. $CB = BK$ and $\frac{BC}{BD} = \frac{BA}{BC} \Rightarrow \frac{BA}{BK} = \frac{BK}{BD}$.

Step 25. $\measuredangle ABK = \measuredangle DBK$ and $\frac{BA}{BK} = \frac{BK}{BD} \Rightarrow \measuredangle BAK = \measuredangle DKB$.

Step 26. A, B, D are collinear, A, X, K are collinear, $\measuredangle BAK = \measuredangle DKB$ and $\measuredangle XAD = \measuredangle BED \Rightarrow \measuredangle BKD = \measuredangle BED$.

Step 27. $\measuredangle BKD = \measuredangle BED \Rightarrow B, D, K, E$ are cyclic.

Step 28. B, D, K, E are cyclic $\Rightarrow \measuredangle DBE = \measuredangle DKE$ and $\measuredangle DBK = \measuredangle DEK$.

Step 29. A, X, K are collinear, D, X, E are collinear, $\measuredangle DBE = \measuredangle DKE$ and $\measuredangle AXD = \measuredangle EBD \Rightarrow \measuredangle DKE = \measuredangle EXK$.

Step 30. D, X, E are collinear $\Rightarrow \measuredangle DEK = \measuredangle XEK$.

Step 31. $\measuredangle DKE = \measuredangle EXK$ and $\measuredangle DEK = \measuredangle XEK \Rightarrow \frac{DK}{DE} = \frac{KX}{KE}$ and $\frac{KD}{KE} = \frac{XK}{XE}$.

Step 32. CA is perpendicular to CB and AD is perpendicular to $DX \Rightarrow \measuredangle(CA, DX) = \measuredangle(CB, AD)$.

Step 33. C, D, X are collinear, A, B, D are collinear and $\measuredangle(CA, DX) = \measuredangle(CB, AD) \Rightarrow \measuredangle ACD = \measuredangle CBA$.

Step 34. A, B, D are collinear, CA is perpendicular to CB and CD is perpendicular to $AB \Rightarrow \measuredangle ACB = \measuredangle CDA$.

Step 35. $\measuredangle ACD = \measuredangle CBA$ and $\measuredangle ACB = \measuredangle CDA \Rightarrow \frac{AC}{AD} = \frac{AB}{AC}$.

Step 36. $CA = AL$ and $\frac{AC}{AD} = \frac{AB}{AC} \Rightarrow \frac{AB}{AL} = \frac{AL}{AD}$.

Step 37. A, B, D are collinear $\Rightarrow \measuredangle BAL = \measuredangle DAL$.

Step 38. $\measuredangle BAL = \measuredangle DAL$ and $\frac{AB}{AL} = \frac{AL}{AD} \Rightarrow \measuredangle ADL = \measuredangle BLA$.

Step 39. B, X, L are collinear and $\measuredangle ADL = \measuredangle BLA \Rightarrow \measuredangle ADL = \measuredangle XLA$.

Step 40. $\measuredangle(AD, XL) = \measuredangle(DX, AE) \Rightarrow \measuredangle ADX = \measuredangle(XL, AE)$.

Step 41. $\measuredangle ADX = \measuredangle(XL, AE)$ and $\measuredangle ADL = \measuredangle XLA \Rightarrow \measuredangle EAL = \measuredangle XDL$.

Step 42. $\measuredangle EAL = \measuredangle XDL$ and DX is parallel to $DE \Rightarrow \measuredangle ALD = \measuredangle AED$.

Step 43. $\measuredangle ALD = \measuredangle AED \Rightarrow A, D, L, E$ are cyclic.

Step 44. A, D, L, E are cyclic $\Rightarrow \measuredangle ADL = \measuredangle AEL$ and $\measuredangle ADE = \measuredangle ALE$.

Step 45. D, X, E are collinear, $\measuredangle DAE = \measuredangle DXL$ and $\measuredangle ADL = \measuredangle AEL \Rightarrow \measuredangle DLE = \measuredangle EXL$.

Step 46. D, X, E are collinear $\Rightarrow \measuredangle DEL = \measuredangle XEL$.

Step 47. $\measuredangle DLE = \measuredangle EXL$ and $\measuredangle DEL = \measuredangle XEL \Rightarrow \frac{DL}{DE} = \frac{LX}{LE}$ and $\frac{LD}{LE} = \frac{XL}{XE}$.

Step 48. $\frac{DK}{DE} = \frac{KX}{KE}$, $\frac{DL}{DE} = \frac{LX}{LE}$, $\frac{KD}{KE} = \frac{XK}{XE}$ and $\frac{LD}{LE} = \frac{XL}{XE} \Rightarrow$ by ratio chasing: $KE = LE$.

Step 49. $KE = LE \Rightarrow \measuredangle KLE = \measuredangle EKL$.

Step 50. A, B, D are collinear, A, L, M are collinear, B, K, M are collinear, $\measuredangle DBK = \measuredangle DEK$ and $\measuredangle ADE = \measuredangle ALE \Rightarrow \measuredangle MKE = \measuredangle MLE$.

Step 51. $\measuredangle MKE = \measuredangle MLE \Rightarrow K, L, M, E$ are cyclic.

Step 52. K, L, M, E are cyclic $\Rightarrow \measuredangle LKM = \measuredangle LEM$ and $\measuredangle KLM = \measuredangle KEM$.

Step 53. C, D, X are collinear, A, B, D are collinear, B, K, M are collinear, D, X, E are collinear, $\measuredangle DBK = \measuredangle DEK$ and CD is perpendicular to $AB \Rightarrow KM$ is perpendicular to KE.

Step 54. C, D, X are collinear, A, B, D are collinear, D, X, E are collinear, $\measuredangle ADE = \measuredangle ALE$ and CD is perpendicular to $AB \Rightarrow AL$ is perpendicular to LE.

Step 55. AL is perpendicular to LE and KM is perpendicular to $KE \Rightarrow \measuredangle(AL, KE) = \measuredangle(LE, KM)$.

Step 56. $\measuredangle LKM = \measuredangle LEM$, $\measuredangle KLM = \measuredangle KEM$ and $\measuredangle KLE = \measuredangle EKL \Rightarrow ME$ is the bisector of $\measuredangle KML$.

Step 57. A, L, M are collinear and $\measuredangle(AL, KE) = \measuredangle(LE, KM) \Rightarrow \measuredangle EKM = \measuredangle MLE$.

Step 58. $\measuredangle EKM = \measuredangle MLE$ and ME is the bisector of $\measuredangle KML \Rightarrow \frac{MK}{ML} = \frac{EK}{EL}$.

Step 59. $KE = LE$ and $\frac{MK}{ML} = \frac{EK}{EL} \Rightarrow KM = LM$

■

2.19 IMO 2013 P4

Original:

Let ABC be a triangle with orthocenter H, and let W be a point on the side BC, between B and C. The points M and N are the feet of the altitudes drawn from B and C, respectively. Suppose W1 is the circumcircle of triangle BW N and X is a point such that W X is a diameter of W1. Similarly, W2 is the circumcircle of triangle CWM and Y is a point such that W Y is a diameter of W2. Show that the points X, Y, and H are collinear.

Translated:

Let ABC be a triangle. Define point H as the orthocenter of triangle CBA. Define point M as the intersection of lines AC and BH. Define point N as the intersection of lines AB and CH. Define point W on line BC such that BC is parallel to BW. Define point O_1 as the circumcenter of triangle WBN. Define

point O_2 as the circumcenter of triangle WCM. Define point X as the mirror of W through O_1. Define point Y as the mirror of W through O_2. Prove that H, X, Y are collinear

Proof:

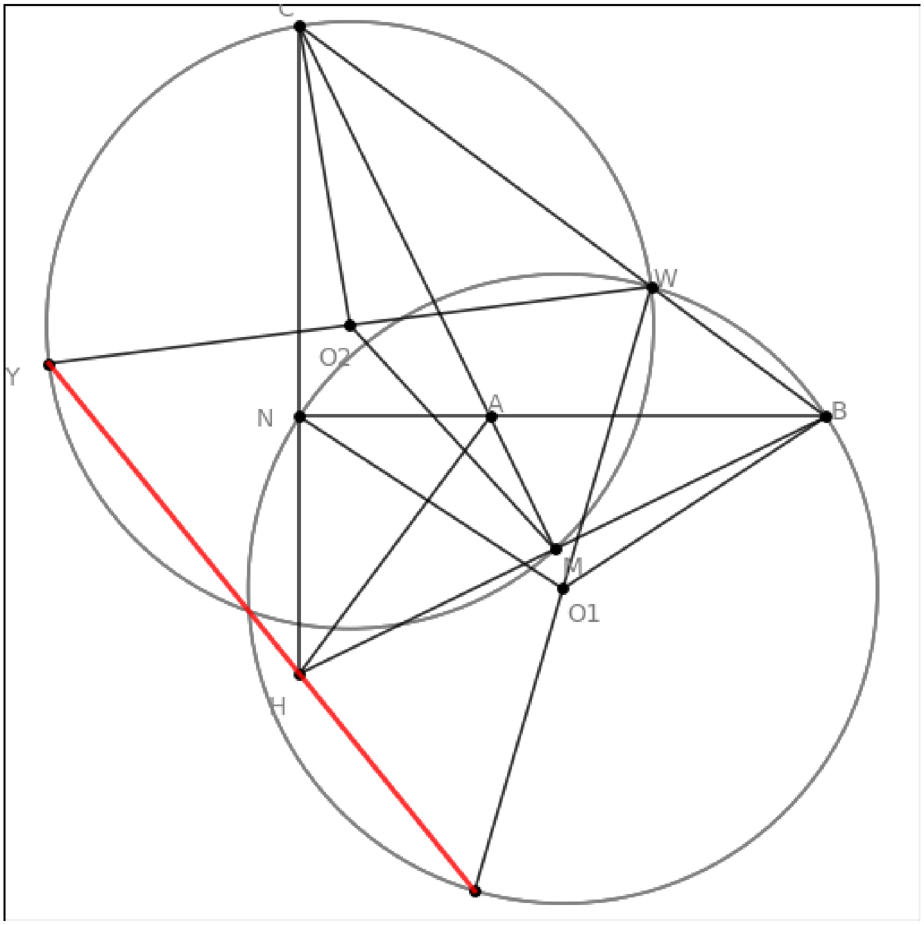

Figure 19: imo 2013 p4

Step 1. B, C, W are collinear and AH is perpendicular to $BC \Rightarrow AH$ is perpendicular to CW.

Step 2. AC is perpendicular to BH and AH is perpendicular to $CW \Rightarrow \measuredangle CAH = \measuredangle(BH, CW)$.

Step 3. A, C, M are collinear, B, C, W are collinear, B, H, M are collinear and $\measuredangle CAH = \measuredangle(BH, CW) \Rightarrow \measuredangle HAM = \measuredangle CBM$.

Step 4. A, C, M are collinear, B, H, M are collinear and AC is perpendicular to $BH \Rightarrow \measuredangle AMH = \measuredangle BMC$.

Step 5. $\measuredangle HAM = \measuredangle CBM$ and $\measuredangle AMH = \measuredangle BMC \Rightarrow \frac{MA}{MB} = \frac{MH}{MC}$.

Step 6. A, C, M are collinear, B, H, M are collinear and AC is perpendicular to $BH \Rightarrow \measuredangle AMB = \measuredangle HMC$.

Step 7. $\measuredangle AMB = \measuredangle HMC$ and $\frac{MA}{MB} = \frac{MH}{MC} \Rightarrow \measuredangle ABM = \measuredangle HCM$ and $\measuredangle BAM = \measuredangle CHM$.

Step 8. $NO_1 = O_1W$ and $O_1W = O_1X \Rightarrow O_1$ is the circumcenter of NWX.

Step 9. O_1 is the circumcenter of NWX and O_1, W, X are collinear $\Rightarrow NW$ is perpendicular to NX.

Step 10. AC is perpendicular to BH and NW is perpendicular to $NX \Rightarrow \measuredangle(AC, BH) = \measuredangle XNW$.

Step 11. $BO_1 = NO_1$, $NO_1 = O_1W$ and $O_1W = O_1X \Rightarrow O_1$ is the circumcenter of BWX.

Step 12. O_1 is the circumcenter of BWX and O_1, W, X are collinear $\Rightarrow BW$ is perpendicular to BX.

Step 13. $BO_1 = NO_1$, $NO_1 = O_1W$ and $O_1W = O_1X \Rightarrow B, N, W, X$ are cyclic.

Step 14. B, N, W, X are cyclic $\Rightarrow \measuredangle BNW = \measuredangle BXW$.

Step 15. A, B, N are collinear, A, C, M are collinear, B, H, M are collinear, C, H, N are collinear, $\measuredangle(AC, BH) = \measuredangle XNW$ and $\measuredangle ABM = \measuredangle HCM \Rightarrow \measuredangle ANH = \measuredangle WNX$.

Step 16. A, B, N are collinear, $\measuredangle BNW = \measuredangle BXW$, BC is parallel to BW, AH is perpendicular to BC and BW is perpendicular to $BX \Rightarrow \measuredangle HAN = \measuredangle XWN$.

Step 17. $\measuredangle HAN = \measuredangle XWN$ and $\measuredangle ANH = \measuredangle WNX \Rightarrow \frac{NA}{NW} = \frac{NH}{NX}$.

Step 18. A, B, N are collinear, A, C, M are collinear, B, H, M are collinear, $\measuredangle BAM = \measuredangle CHM$ and $\measuredangle ABM = \measuredangle HCM \Rightarrow AN$ is perpendicular to CH.

Step 19. AN is perpendicular to CH and NW is perpendicular to $NX \Rightarrow \measuredangle ANW = \measuredangle(CH, NX)$.

Step 20. C, H, N are collinear and $\measuredangle ANW = \measuredangle(CH, NX) \Rightarrow \measuredangle ANW = \measuredangle HNX$.

Step 21. $\measuredangle ANW = \measuredangle HNX$ and $\frac{NA}{NW} = \frac{NH}{NX} \Rightarrow \measuredangle(AW, HX) = \measuredangle WNX$.

Step 22. $CO_2 = MO_2$, $MO_2 = O_2W$ and $O_2W = O_2Y \Rightarrow O_2$ is the circumcenter of CWY.

Step 23. O_2 is the circumcenter of CWY and O_2, W, Y are collinear $\Rightarrow CW$ is perpendicular to CY.

Step 24. $CO_2 = MO_2$, $MO_2 = O_2W$ and $O_2W = O_2Y \Rightarrow C, M, W, Y$ are cyclic.

Step 25. C, M, W, Y are cyclic $\Rightarrow \measuredangle CMW = \measuredangle CYW$.

Step 26. $MO_2 = O_2W$ and $O_2W = O_2Y \Rightarrow O_2$ is the circumcenter of MWY.

Step 27. O_2 is the circumcenter of MWY and O_2, W, Y are collinear $\Rightarrow MW$ is perpendicular to MY.

Step 28. AC is perpendicular to BH and MW is perpendicular to $MY \Rightarrow \measuredangle(AC, MW) = \measuredangle(BH, MY)$ and $\measuredangle(AC, BH) = \measuredangle YMW$.

Step 29. A, C, M are collinear, B, C, W are collinear, $\measuredangle CMW = \measuredangle CYW$, AH is perpendicular to BC and CW is perpendicular to $CY \Rightarrow \measuredangle HAM = \measuredangle YWM$.

Step 30. A, C, M are collinear, B, H, M are collinear, AC is perpendicular to BH and MW is perpendicular to $MY \Rightarrow \measuredangle AMH = \measuredangle WMY$.

Step 31. $\measuredangle HAM = \measuredangle YWM$ and $\measuredangle AMH = \measuredangle WMY \Rightarrow \frac{MA}{MW} = \frac{MH}{MY}$.

Step 32. A, C, M are collinear, B, H, M are collinear and $\measuredangle(AC, MW) = \measuredangle(BH, MY) \Rightarrow \measuredangle AMW = \measuredangle HMY$.

Step 33. $\measuredangle AMW = \measuredangle HMY$ and $\frac{MA}{MW} = \frac{MH}{MY} \Rightarrow \measuredangle MAW = \measuredangle MHY$ and $\measuredangle(AW, HY) = \measuredangle WMY$.

Step 34. A, C, M are collinear, B, H, M are collinear, $\measuredangle(AC, BH) = \measuredangle YMW$, $\measuredangle MAW = \measuredangle MHY$ and $\measuredangle(AW, HY) = \measuredangle WMY \Rightarrow AW$ is perpendicular to HY.

Step 35. AW is perpendicular to HY and NW is perpendicular to $NX \Rightarrow \measuredangle(AW, HY) = \measuredangle WNX$.

Step 36. $\measuredangle(AW, HX) = \measuredangle WNX$ and $\measuredangle(AW, HY) = \measuredangle WNX \Rightarrow \measuredangle(AW, HX) = \measuredangle(AW, HY)$.

Step 37. $\measuredangle(AW, HX) = \measuredangle(AW, HY) \Rightarrow HX$ is parallel to HY.

Step 38. HX is parallel to $HY \Rightarrow H, X, Y$ are collinear

■

2.20 IMO 2014 P4

Original:

Let P and Q be on segment BC of a triangle ABC such that $\angle PAB = \angle BCA$ and $\angle CAQ = \angle ABC$. Let M and N be the points on AP and AQ, respectively, such that P is the midpoint of AM and Q is the midpoint of AN. Prove that the intersection of BM and CN is on the circumference of triangle ABC.

Translated:

Let ABC be a triangle. Define point P on line BC such that $\measuredangle BAC = \measuredangle(AP, BC)$. Define point Q on line BC such that $\measuredangle CAB = \measuredangle(AQ, BC)$ and $\measuredangle ABC = \measuredangle CAQ$. Define point M as the mirror of A through P. Define point N as the mirror of A through Q. Define point X as the intersection of lines BM and CN. Define point O as the circumcenter of triangle CBA. Prove that $AO = XO$

Proof:

Construct point L as the mirror of C through O.

Step 1. B, C, P are collinear, B, C, Q are collinear, $\measuredangle BAC = \measuredangle(AP, BC)$ and $\measuredangle CAB = \measuredangle(AQ, BC) \Rightarrow \measuredangle AQC = \measuredangle BPA$.

Step 2. B, C, P are collinear and $\measuredangle ABC = \measuredangle CAQ \Rightarrow \measuredangle ABP = \measuredangle CAQ$.

Step 3. $\measuredangle ABP = \measuredangle CAQ$ and $\measuredangle AQC = \measuredangle BPA \Rightarrow \frac{QA}{QC} = \frac{PB}{PA}$.

Step 4. $AP = PM$, $AQ = QN$ and $\frac{QA}{QC} = \frac{PB}{PA} \Rightarrow \frac{PB}{PM} = \frac{QN}{QC}$.

Step 5. A, P, M are collinear, A, Q, N are collinear, B, C, P are collinear, B, C, Q are collinear, $\measuredangle BAC = \measuredangle(AP, BC)$ and $\measuredangle CAB = \measuredangle(AQ, BC) \Rightarrow \measuredangle BPM = \measuredangle NQC$.

Step 6. $\measuredangle BPM = \measuredangle NQC$ and $\frac{PB}{PM} = \frac{QN}{QC} \Rightarrow \measuredangle BMP = \measuredangle NCQ$.

Step 7. A, P, M are collinear, B, C, Q are collinear, B, M, X are collinear, C, N, X are collinear, $\measuredangle BAC = \measuredangle(AP, BC)$ and $\measuredangle BMP = \measuredangle NCQ \Rightarrow \measuredangle BAC =$

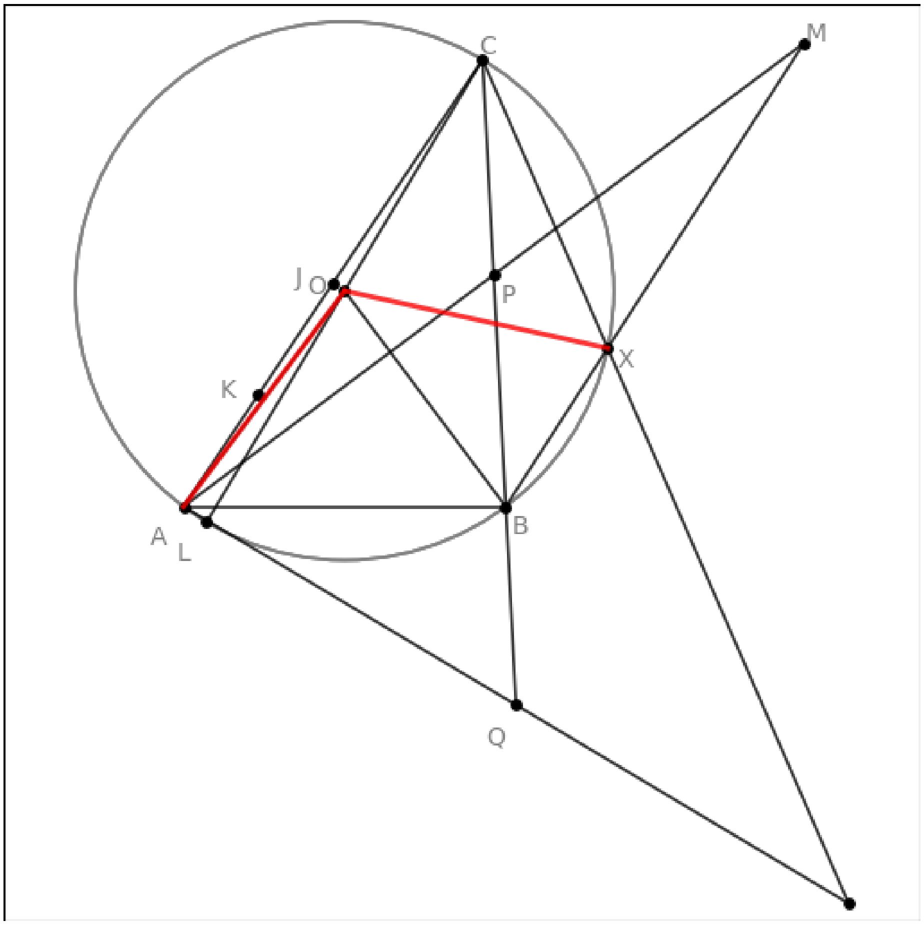

Figure 20: imo 2014 p4

$\measuredangle BXC$.

Step 8. $\measuredangle BAC = \measuredangle BXC \Rightarrow A, B, C, X$ are cyclic.

Step 9. $AO = BO$, $BO = CO$, $CO = OL$ and A, B, C, X are cyclic $\Rightarrow$ A, C, X, L are cyclic.

Step 10. A, C, X, L are cyclic $\Rightarrow \measuredangle ACX = \measuredangle ALX$.

Step 11. $AO = BO$, $BO = CO$, $CO = OL$ and A, B, C, X are cyclic $\Rightarrow$ B, C, X, L are cyclic.

Step 12. B, C, X, L are cyclic $\Rightarrow \measuredangle BCX = \measuredangle BLX$.

Step 13. $BO = CO$ and $CO = OL \Rightarrow O$ is the circumcenter of BCL.

Step 14. O is the circumcenter of BCL and C, O, L are collinear $\Rightarrow BC$ is perpendicular to BL.

Step 15. $AO = BO$, $BO = CO$ and $CO = OL \Rightarrow O$ is the circumcenter of ACL.

Step 16. O is the circumcenter of ACL and C, O, L are collinear $\Rightarrow AC$ is perpendicular to AL.

Step 17. B, C, Q are collinear and BC is perpendicular to $BL \Rightarrow BQ$ is perpendicular to BL.

Step 18. AC is perpendicular to AL and BQ is perpendicular to $BL \Rightarrow \measuredangle(AC, BL) = \measuredangle(AL, BQ)$.

Step 19. B, C, Q are collinear, $\measuredangle(AC, BL) = \measuredangle(AL, BQ)$, $\measuredangle ACX = \measuredangle ALX$ and $\measuredangle BCX = \measuredangle BLX \Rightarrow CX$ is perpendicular to XL.

Step 20. C, O, L are collinear and $CO = OL \Rightarrow O$ is the midpoint of CL.

Step 21. O is the midpoint of CL and CX is perpendicular to $XL \Rightarrow CO = XO$.

Step 22. $AO = BO$, $BO = CO$ and $CO = XO \Rightarrow AO = XO$

■

2.21 IMO 2015 P3

Original:

Let ABC be a triangle with AB ¿ AC. Let Gamma be its circumcircle, H its orthocenter, and F the foot of the altitude from A. Let M be the midpoint of BC. Let Q be the point on Gamma such that $\measuredangle HQA = 90^o$ and let K be the point on Gamma such that $\measuredangle HKQ = 90^o$. Assume that the points A, B, C, K and Q are all different and lie on Gamma in this order. Prove that the circumcircles of triangles KQH and FKM are tangent to each other.

Translated:

Let ABC be a triangle. Define point H as the orthocenter of triangle CBA. Define point F as the intersection of lines AH and BC. Define point M as the midpoint of BC. Define point O as the circumcenter of triangle CBA. Define point Q on circle (O, A) such that AQ is perpendicular to HQ. Define point K on circle (O, A) such that HK is perpendicular to KQ. Define point O_1 as the circumcenter of triangle KHQ. Define point O_2 as the circumcenter of triangle FMK. Prove that K, O_1, O_2 are collinear

Proof:

Construct point X as the midpoint of CH.

Construct point Y as the midpoint of KM.

Construct point Z as the midpoint of BH.

Step 1. B, C, M are collinear and AH is perpendicular to $BC \Rightarrow AH$ is perpendicular to CM.

Step 2. B, H, Z are collinear and AC is perpendicular to $BH \Rightarrow AC$ is perpendicular to HZ.

Step 3. AC is perpendicular to HZ and AH is perpendicular to $CM \Rightarrow \measuredangle HAC = \measuredangle(CM, HZ)$ and $\measuredangle AHZ = \measuredangle MCA$.

Step 4. A, F, H are collinear, B, C, F are collinear, B, C, M are collinear, B, H, Z are collinear and $\measuredangle HAC = \measuredangle(CM, HZ) \Rightarrow \measuredangle CAF = \measuredangle HBF$.

Step 5. A, F, H are collinear, B, C, F are collinear and AH is perpendicular to $BC \Rightarrow \measuredangle AFC = \measuredangle BFH$.

Step 6. $\measuredangle CAF = \measuredangle HBF$ and $\measuredangle AFC = \measuredangle BFH \Rightarrow \frac{FA}{FB} = \frac{FC}{FH}$.

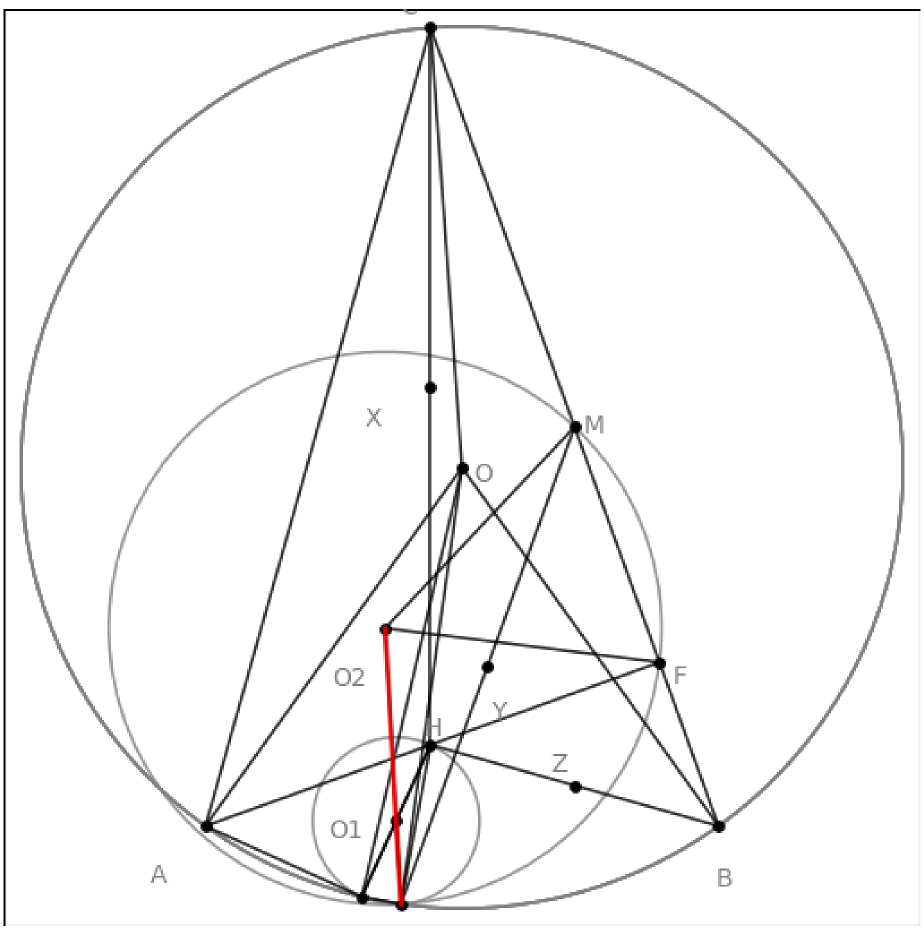

Figure 21: imo 2015 p3

Step 7. A, F, H are collinear, B, C, F are collinear and AH is perpendicular to $BC \Rightarrow \measuredangle AFB = \measuredangle CFH$.

Step 8. $\measuredangle AFB = \measuredangle CFH$ and $\frac{FA}{FB} = \frac{FC}{FH} \Rightarrow \measuredangle BAF = \measuredangle HCF$ and $\measuredangle ABF = \measuredangle CHF$.

Step 9. $BM = CM$ and $BO = CO \Rightarrow BC$ is perpendicular to MO.

Step 10. B, C, M are collinear and $BM = CM \Rightarrow M$ is the midpoint of BC.

Step 11. C, H, X are collinear and $CX = HX \Rightarrow X$ is the midpoint of CH.

Step 12. M is the midpoint of BC and X is the midpoint of $CH \Rightarrow BH$ is parallel to MX.

Step 13. B, H, Z are collinear and $BZ = HZ \Rightarrow Z$ is the midpoint of BH.

Step 14. M is the midpoint of BC and Z is the midpoint of $BH \Rightarrow CH$ is parallel to MZ.

Step 15. $HO_1 = O_1Q$ and $KO_1 = O_1Q \Rightarrow O_1$ is the circumcenter of HKQ.

Step 16. AQ is perpendicular to HQ and HK is perpendicular to $KQ \Rightarrow \measuredangle AQH = \measuredangle QKH$.

Step 17. O_1 is the circumcenter of HKQ and $\measuredangle AQH = \measuredangle QKH \Rightarrow AQ$ is perpendicular to O_1Q.

Step 18. $HO_1 = O_1Q$, AQ is perpendicular to HQ and AQ is perpendicular to $O_1Q \Rightarrow O_1$ is the midpoint of HQ.

Step 19. O_1 is the midpoint of HQ and X is the midpoint of $CH \Rightarrow CQ$ is parallel to O_1X.

Step 20. O_1 is the midpoint of HQ and Z is the midpoint of $BH \Rightarrow BQ$ is parallel to O_1Z.

Step 21. $AO = BO$, $AO = OQ$ and $BO = CO \Rightarrow A, B, C, Q$ are cyclic.

Step 22. A, B, C, Q are cyclic $\Rightarrow \measuredangle ABQ = \measuredangle ACQ$ and $\measuredangle ACB = \measuredangle AQB$.

Step 23. A, F, H are collinear, B, C, F are collinear, B, C, M are collinear, B, H, Z are collinear, $\measuredangle BAF = \measuredangle HCF$, $\measuredangle HAC = \measuredangle(CM, HZ)$, BH is parallel to MX and CH is parallel to $MZ \Rightarrow \measuredangle(AB, MZ) = \measuredangle(AC, MX)$.

Step 24. $\measuredangle ABQ = \measuredangle ACQ$, BQ is parallel to O_1Z and CQ is parallel to $O_1X \Rightarrow \measuredangle(AB, O_1Z) = \measuredangle(AC, O_1X)$.

Step 25. $\measuredangle(AB, MZ) = \measuredangle(AC, MX)$ and $\measuredangle(AB, O_1Z) = \measuredangle(AC, O_1X) \Rightarrow \measuredangle XMZ = \measuredangle XO_1Z$.

Step 26. $\measuredangle XMZ = \measuredangle XO_1Z \Rightarrow M, O_1, X, Z$ are cyclic.

Step 27. A, F, H are collinear, B, C, F are collinear and AH is perpendicular to $BC \Rightarrow BF$ is perpendicular to FH.

Step 28. Z is the midpoint of BH and BF is perpendicular to $FH \Rightarrow BZ = FZ$.

Step 29. $BZ = FZ \Rightarrow \measuredangle BFZ = \measuredangle ZBF$.

Step 30. X is the midpoint of CH and Z is the midpoint of $BH \Rightarrow BC$ is parallel to XZ.

Step 31. B, C, F are collinear, B, C, M are collinear, B, H, Z are collinear, $\measuredangle BFZ = \measuredangle ZBF$, BC is parallel to XZ and BH is parallel to $MX \Rightarrow \measuredangle MFZ = \measuredangle MXZ$.

Step 32. $\measuredangle MFZ = \measuredangle MXZ \Rightarrow F, M, X, Z$ are cyclic.

Step 33. F, M, X, Z are cyclic and M, O_1, X, Z are cyclic $\Rightarrow F, M, O_1, X$ are cyclic.

Step 34. F, M, O_1, X are cyclic and F, M, X, Z are cyclic $\Rightarrow F, O_1, X, Z$ are cyclic.

Step 35. F, O_1, X, Z are cyclic $\Rightarrow \measuredangle O_1FX = \measuredangle O_1ZX$.

Step 36. AC is perpendicular to HZ and AQ is perpendicular to $HQ \Rightarrow \measuredangle CAQ = \measuredangle ZHQ$ and $\measuredangle(AC, HQ) = \measuredangle(HZ, AQ)$.

Step 37. B, H, Z are collinear, $\measuredangle CAQ = \measuredangle ZHQ$, BH is parallel to MX, AQ is perpendicular to HQ and AQ is perpendicular to $O_1Q \Rightarrow \measuredangle(AC, MX) = \measuredangle(AQ, HO_1)$.

Step 38. B, C, M are collinear and $\measuredangle ACB = \measuredangle AQB \Rightarrow \measuredangle ACM = \measuredangle AQB$.

Step 39. $\measuredangle(AC, MX) = \measuredangle(AQ, HO_1)$ and $\measuredangle ACM = \measuredangle AQB \Rightarrow \measuredangle(BQ, HO_1) = \measuredangle CMX$.

Step 40. $AO = BO$ and $BO = CO \Rightarrow O$ is the circumcenter of ABC.

Step 41. O is the circumcenter of ABC and M is the midpoint of $BC \Rightarrow \measuredangle(AB, MO) = \measuredangle ACO$ and $\measuredangle BAC = \measuredangle MOC$.

Step 42. $AO = BO$ and $BO = CO \Rightarrow AO = CO$.

Step 43. $AO = CO \Rightarrow \measuredangle ACO = \measuredangle OAC$.

Step 44. B, H, Z are collinear, $\measuredangle HAC = \measuredangle(CM, HZ)$ and BH is parallel to $MX \Rightarrow \measuredangle(AC, MX) = \measuredangle(AH, CM)$.

Step 45. $\measuredangle(AB, MO) = \measuredangle ACO$, $\measuredangle ACO = \measuredangle OAC$, AH is perpendicular to BC and BC is perpendicular to $MO \Rightarrow \measuredangle BAH = \measuredangle OAC$.

Step 46. $\measuredangle BAH = \measuredangle OAC$ and $\measuredangle(AC, MX) = \measuredangle(AH, CM) \Rightarrow \measuredangle(AB, CM) = \measuredangle(AO, MX)$.

Step 47. A, F, H are collinear, B, C, F are collinear and AH is perpendicular to $BC \Rightarrow CF$ is perpendicular to FH.

Step 48. X is the midpoint of CH and CF is perpendicular to $FH \Rightarrow FX = HX$.

Step 49. $FX = HX \Rightarrow \measuredangle FHX = \measuredangle XFH$.

Step 50. B, C, M are collinear, $\measuredangle(BQ, HO_1) = \measuredangle CMX$, $\measuredangle O_1FX = \measuredangle O_1ZX$, BC is parallel to XZ and BQ is parallel to $O_1Z \Rightarrow \measuredangle O_1FX = \measuredangle(HO_1, MX)$.

Step 51. A, F, H are collinear, B, C, F are collinear, B, C, M are collinear, C, H, X are collinear, $\measuredangle(AB, CM) = \measuredangle(AO, MX)$, $\measuredangle ABF = \measuredangle CHF$ and $\measuredangle FHX = \measuredangle XFH \Rightarrow \measuredangle(AH, FX) = \measuredangle(AO, MX)$.

Step 52. $\measuredangle(AH, FX) = \measuredangle(AO, MX)$ and $\measuredangle O_1FX = \measuredangle(HO_1, MX) \Rightarrow \measuredangle(AH, FO_1) = \measuredangle(AO, HO_1)$.

Step 53. B, C, M are collinear, B, H, Z are collinear, $\measuredangle ACB = \measuredangle AQB$, $\measuredangle AHZ = \measuredangle MCA$ and BH is parallel to $MX \Rightarrow \measuredangle(AH, MX) = \measuredangle BQA$.

Step 54. B, H, Z are collinear, $\measuredangle(AC, HQ) = \measuredangle(HZ, AQ)$, BH is parallel to MX, AQ is perpendicular to HQ and AQ is perpendicular to $O_1Q \Rightarrow \measuredangle(AC, MX) = \measuredangle(HO_1, AQ)$.

Step 55. $\measuredangle(AC, MX) = \measuredangle(HO_1, AQ)$ and $\measuredangle(AH, MX) = \measuredangle BQA \Rightarrow \measuredangle HAC = \measuredangle(BQ, HO_1)$.

Step 56. $AO = BO$, $AO = OQ$ and $BO = CO \Rightarrow O$ is the circumcenter of BCQ.

Step 57. O is the circumcenter of BCQ and M is the midpoint of $BC \Rightarrow \measuredangle(BQ, MO) = \measuredangle QCO$.

Step 58. $\measuredangle HAC = \measuredangle(BQ, HO_1)$, $\measuredangle(BQ, MO) = \measuredangle QCO$, CQ is parallel to O_1X, AH is perpendicular to BC and BC is perpendicular to $MO \Rightarrow \measuredangle(AC, HO_1) = \measuredangle(CO, O_1X)$.

Step 59. $\measuredangle BAC = \measuredangle MOC$, AH is perpendicular to BC and BC is perpendicular to $MO \Rightarrow \measuredangle BAC = \measuredangle(AH, CO)$.

Step 60. $\measuredangle BAC = \measuredangle(AH, CO)$ and $\measuredangle(AC, HO_1) = \measuredangle(CO, O_1X) \Rightarrow \measuredangle(AB, HO_1) = \measuredangle(AH, O_1X)$.

Step 61. A, F, H are collinear, B, C, F are collinear, B, C, M are collinear, $\measuredangle BAF = \measuredangle HCF$ and CH is parallel to $MZ \Rightarrow \measuredangle(AB, MZ) = \measuredangle(AH, CM)$.

Step 62. $\measuredangle(AB, HO_1) = \measuredangle(AH, O_1X)$ and $\measuredangle(AB, MZ) = \measuredangle(AH, CM) \Rightarrow \measuredangle(CM, O_1X) = \measuredangle(MZ, HO_1)$.

Step 63. M, O_1, X, Z are cyclic $\Rightarrow \measuredangle O_1MZ = \measuredangle O_1XZ$.

Step 64. B, C, M are collinear, $\measuredangle(CM, O_1X) = \measuredangle(MZ, HO_1)$, $\measuredangle O_1MZ = \measuredangle O_1XZ$ and BC is parallel to $XZ \Rightarrow \measuredangle(HO_1, MZ) = \measuredangle O_1MZ$.

Step 65. $\measuredangle(HO_1, MZ) = \measuredangle O_1MZ \Rightarrow HO_1$ is parallel to MO_1.

Step 66. HO_1 is parallel to $MO_1 \Rightarrow H, M, O_1$ are collinear.

Step 67. AH is perpendicular to CM and AQ is perpendicular to $HQ \Rightarrow \measuredangle HAQ = \measuredangle(CM, HQ)$.

Step 68. A, F, H are collinear, B, C, F are collinear, B, C, M are collinear, H, M, O_1 are collinear, $\measuredangle HAQ = \measuredangle(CM, HQ)$, AQ is perpendicular to HQ and AQ is perpendicular to $O_1Q \Rightarrow \measuredangle AFM = \measuredangle AQM$.

Step 69. $\measuredangle AFM = \measuredangle AQM \Rightarrow A, F, M, Q$ are cyclic.

Step 70. A, F, M, Q are cyclic $\Rightarrow \measuredangle AFQ = \measuredangle AMQ$.

Step 71. $\measuredangle(AH, FO_1) = \measuredangle(AO, HO_1)$, AH is perpendicular to BC, AQ is perpendicular to HQ, AQ is perpendicular to O_1Q and BC is perpendicular to $MO \Rightarrow \measuredangle AOM = \measuredangle QO_1F$.

Step 72. A, F, H are collinear, H, M, O_1 are collinear, $\measuredangle AFQ = \measuredangle AMQ$, AH is perpendicular to BC, AQ is perpendicular to HQ, AQ is perpendicular to O_1Q and BC is perpendicular to $MO \Rightarrow \measuredangle AMO = \measuredangle O_1QF$.

Step 73. $\measuredangle AMO = \measuredangle O_1QF$ and $\measuredangle AOM = \measuredangle QO_1F \Rightarrow \frac{OA}{OM} = \frac{O_1F}{O_1Q}$.

Step 74. F, M, O_1, X are cyclic $\Rightarrow \measuredangle MFO_1 = \measuredangle MXO_1$.

Step 75. $AO = KO$ and $AO = OQ \Rightarrow KO = OQ$.

Step 76. $KO = OQ$ and $KO_1 = O_1Q \Rightarrow O_1O$ is the bisector of $\measuredangle KO_1Q$.

Step 77. $KO = OQ$ and $KO_1 = O_1Q \Rightarrow KQ$ is perpendicular to OO_1.

Step 78. $AO = BO$, $AO = KO$ and $BO = CO \Rightarrow A, B, C, K$ are cyclic.

Step 79. A, B, C, K are cyclic and A, B, C, Q are cyclic $\Rightarrow A, C, K, Q$ are cyclic.

Step 80. A, C, K, Q are cyclic $\Rightarrow \measuredangle ACK = \measuredangle AQK$ and $\measuredangle CAK = \measuredangle CQK$.

Step 81. AC is perpendicular to HZ and HK is perpendicular to $KQ \Rightarrow \measuredangle(AC, HK) = \measuredangle(HZ, KQ)$.

Step 82. $\measuredangle ACK = \measuredangle AQK$, O_1O is the bisector of $\measuredangle KO_1Q$, AQ is perpendicular to O_1Q and KQ is perpendicular to $OO_1 \Rightarrow \measuredangle ACK = \measuredangle OO_1K$.

Step 83. B, H, Z are collinear, $\measuredangle(AC, HK) = \measuredangle(HZ, KQ)$, BH is parallel to MX, HK is perpendicular to KQ and KQ is perpendicular to $OO_1 \Rightarrow \measuredangle(AC, MX) = \measuredangle(OO_1, KQ)$.

Step 84. $\measuredangle(AC, MX) = \measuredangle(OO_1, KQ)$ and $\measuredangle ACK = \measuredangle OO_1K \Rightarrow \measuredangle(CK, MX) = \measuredangle O_1KQ$.

Step 85. A, B, C, K are cyclic $\Rightarrow \measuredangle ACB = \measuredangle AKB$.

Step 86. $AO = BO$, $AO = KO$ and $BO = CO \Rightarrow O$ is the circumcenter of BCK.

Step 87. O is the circumcenter of BCK and M is the midpoint of $BC \Rightarrow \measuredangle KBO = \measuredangle(CK, MO)$.

Step 88. $AO = BO$ and $AO = KO \Rightarrow BO = KO$.

Step 89. $BO = KO \Rightarrow \measuredangle BKO = \measuredangle OBK$.

Step 90. B, C, M are collinear, B, H, Z are collinear, $\measuredangle ACB = \measuredangle AKB$, $\measuredangle AHZ = \measuredangle MCA$ and BH is parallel to $MX \Rightarrow \measuredangle(AH, MX) = \measuredangle BKA$.

Step 91. $\measuredangle KBO = \measuredangle(CK, MO)$, $\measuredangle BKO = \measuredangle OBK$, AH is perpendicular to BC and BC is perpendicular to $MO \Rightarrow \measuredangle(AH, CK) = \measuredangle BKO$.

Step 92. $\measuredangle(AH, CK) = \measuredangle BKO$ and $\measuredangle(AH, MX) = \measuredangle BKA \Rightarrow \measuredangle AKO = \measuredangle(MX, CK)$.

Step 93. $\measuredangle(CK, MX) = \measuredangle O_1KQ$ and $\measuredangle AKO = \measuredangle(MX, CK) \Rightarrow \measuredangle AKO = \measuredangle QKO_1$.

Step 94. $\measuredangle CAK = \measuredangle CQK$ and CQ is parallel to $O_1X \Rightarrow \measuredangle CAK = \measuredangle(O_1X, KQ)$.

Step 95. $\measuredangle CAK = \measuredangle(O_1X, KQ)$ and $\measuredangle AKO = \measuredangle QKO_1 \Rightarrow \measuredangle(AC, KO) = \measuredangle XO_1K$.

Step 96. B, C, F are collinear, B, C, M are collinear, B, H, Z are collinear, $\measuredangle HAC = \measuredangle(CM, HZ)$, $\measuredangle MFO_1 = \measuredangle MXO_1$ and BH is parallel to $MX \Rightarrow \measuredangle CAH = \measuredangle XO_1F$.

Step 97. $\measuredangle CAH = \measuredangle XO_1F$ and $\measuredangle(AC, KO) = \measuredangle XO_1K \Rightarrow \measuredangle(AH, KO) = \measuredangle FO_1K$.

Step 98. $AO = KO$, $KO_1 = O_1Q$ and $\frac{OA}{OM} = \frac{O_1F}{O_1Q} \Rightarrow \frac{O_1F}{O_1K} = \frac{OK}{OM}$.

Step 99. $\measuredangle(AH, KO) = \measuredangle FO_1K$, AH is perpendicular to BC and BC is perpendicular to $MO \Rightarrow \measuredangle FO_1K = \measuredangle MOK$.

Step 100. $\measuredangle FO_1K = \measuredangle MOK$ and $\frac{O_1F}{O_1K} = \frac{OK}{OM} \Rightarrow \measuredangle FKO_1 = \measuredangle OMK$.

Step 101. $KO_2 = MO_2$ and $KY = MY \Rightarrow KM$ is perpendicular to O_2Y.

Step 102. $FO_2 = KO_2$ and $KO_2 = MO_2 \Rightarrow O_2$ is the circumcenter of FKM.

Step 103. K, M, Y are collinear and $KY = MY \Rightarrow Y$ is the midpoint of KM.

Step 104. O_2 is the circumcenter of FKM and Y is the midpoint of $KM \Rightarrow \measuredangle KFM = \measuredangle KO_2Y$.

Step 105. B, C, M are collinear, K, M, Y are collinear, AH is perpendicular to BC and KM is perpendicular to $O_2Y \Rightarrow \measuredangle(AH, CM) = \measuredangle KYO_2$.

Step 106. B, C, F are collinear, B, C, M are collinear and $\measuredangle KFM = \measuredangle KO_2Y \Rightarrow \measuredangle(CM, FK) = \measuredangle YO_2K$.

Step 107. $\measuredangle(AH, CM) = \measuredangle KYO_2$ and $\measuredangle(CM, FK) = \measuredangle YO_2K \Rightarrow \measuredangle(AH, FK) = \measuredangle YKO_2$.

Step 108. K, M, Y are collinear, $\measuredangle(AH, FK) = \measuredangle YKO_2$, $\measuredangle FKO_1 = \measuredangle OMK$, AH is perpendicular to BC and BC is perpendicular to $MO \Rightarrow \measuredangle FKO_1 = \measuredangle FKO_2$.

Step 109. $\measuredangle FKO_1 = \measuredangle FKO_2 \Rightarrow KO_1$ is parallel to KO_2.

Step 110. KO_1 is parallel to $KO_2 \Rightarrow K, O_1, O_2$ are collinear

■

2.22 IMO 2015 P4

Original:

Triangle ABC has circumcircle Omega and circumcenter O. A circle Gamma with center A intersects the segment BC at points D and E, such that B, D, E, and C are all different and lie on line BC in this order. Let F and G be the points of intersection of Gamma and Omega, such that A, F, B, C, and G lie on Omega in this order. Let $K = (BDF) \cap AB \neq B$ and $L = (CGE) \cap AC \neq C$

and assume these points do not lie on line F G. Define $X = FK \cap GL$. Prove that X lies on the line AO.

Translated:

Let ABC be a triangle. Define point O as the circumcenter of triangle CBA. Let D be any point on line BC. Define point E as the intersection of circle (A, D) and line BC. Define point F as the intersection of circles (A, D) and (O, A). Define point G as the intersection of circles (A, D) and (O, A). Define point O_1 as the circumcenter of triangle FBD. Define point O_2 as the circumcenter of triangle ECG. Define point K as the intersection of circle (O_1, B) and line AB. Define point L as the intersection of circle (O_2, C) and line AC. Define point X as the intersection of lines FK and GL. Prove that A, O, X are collinear

Proof:

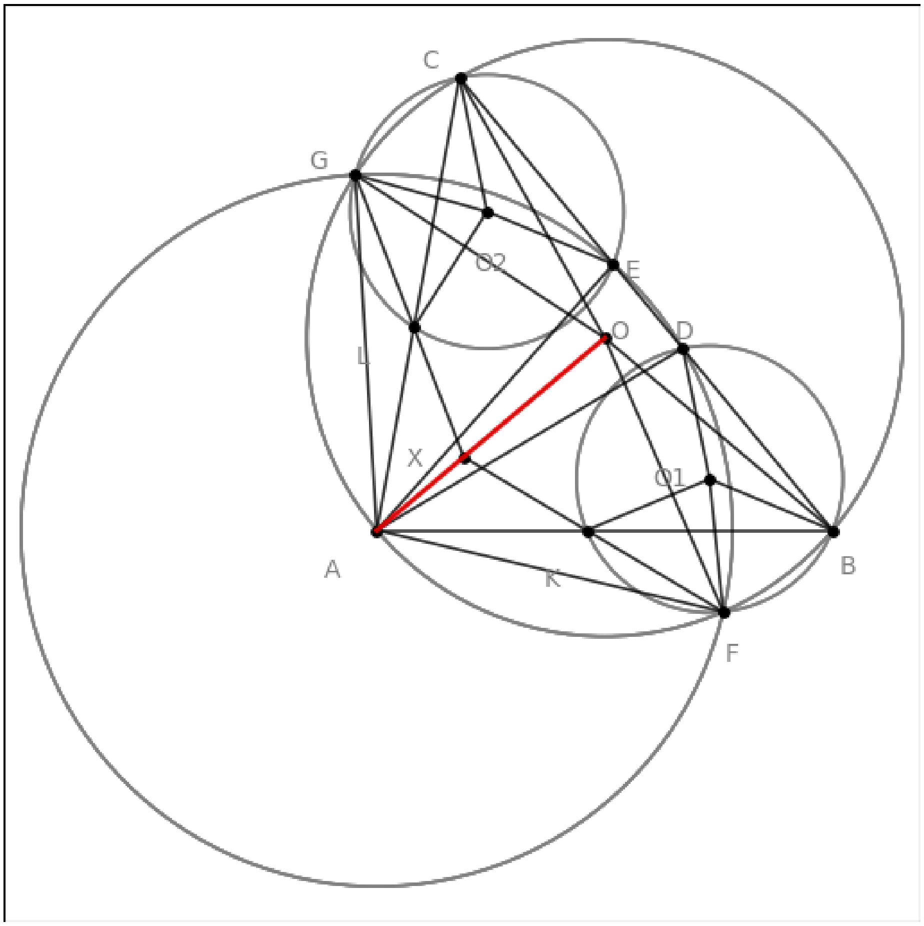

Figure 22: imo 2015 p4

Step 1. $AO = BO$, $AO = FO$, $AO = GO$ and $BO = CO \Rightarrow A, C, F, G$ are

cyclic.

Step 2. $AO = BO$, $AO = FO$, $BO = CO$ and A, C, F, G are cyclic $\Rightarrow$ A, B, F, G are cyclic.

Step 3. A, B, F, G are cyclic $\Rightarrow \measuredangle ABF = \measuredangle AGF$.

Step 4. A, C, F, G are cyclic $\Rightarrow \measuredangle ACG = \measuredangle AFG$.

Step 5. $BO_1 = DO_1$, $BO_1 = FO_1$ and $BO_1 = KO_1 \Rightarrow B, D, F, K$ are cyclic.

Step 6. B, D, F, K are cyclic $\Rightarrow \measuredangle BFD = \measuredangle BKD$ and $\measuredangle BDK = \measuredangle BFK$.

Step 7. $AD = AF$ and $AD = AG \Rightarrow AF = AG$.

Step 8. $AF = AG \Rightarrow \measuredangle AFG = \measuredangle FGA$.

Step 9. A, B, F, G are cyclic and A, C, F, G are cyclic $\Rightarrow B, C, F, G$ are cyclic.

Step 10. B, C, F, G are cyclic $\Rightarrow \measuredangle CBF = \measuredangle CGF$.

Step 11. A, B, K are collinear, A, C, L are collinear, $\measuredangle ABF = \measuredangle AGF$, $\measuredangle ACG = \measuredangle AFG$, $\measuredangle AFG = \measuredangle FGA$ and $\measuredangle BFD = \measuredangle BKD \Rightarrow \measuredangle(AL, CG) = \measuredangle FDK$.

Step 12. B, C, D are collinear, F, K, X are collinear, $\measuredangle CBF = \measuredangle CGF$ and $\measuredangle BDK = \measuredangle BFK \Rightarrow \measuredangle CGF = \measuredangle(DK, FX)$.

Step 13. $\measuredangle(AL, CG) = \measuredangle FDK$ and $\measuredangle CGF = \measuredangle(DK, FX) \Rightarrow \measuredangle(AL, FG) = \measuredangle DFX$.

Step 14. $CO_2 = EO_2$, $CO_2 = GO_2$ and $CO_2 = LO_2 \Rightarrow C, E, G, L$ are cyclic.

Step 15. C, E, G, L are cyclic $\Rightarrow \measuredangle ECL = \measuredangle EGL$.

Step 16. $AD = AE$, $AD = AF$ and $AD = AG \Rightarrow D, E, F, G$ are cyclic.

Step 17. D, E, F, G are cyclic $\Rightarrow \measuredangle EDF = \measuredangle EGF$.

Step 18. A, C, L are collinear, B, C, E are collinear, G, L, X are collinear and $\measuredangle ECL = \measuredangle EGL \Rightarrow \measuredangle(AL, BE) = \measuredangle XGE$.

Step 19. B, C, D are collinear, B, C, E are collinear and $\measuredangle EDF = \measuredangle EGF$ $\Rightarrow \measuredangle(BE, DF) = \measuredangle EGF$.

Step 20. $\measuredangle(AL, BE) = \measuredangle XGE$ and $\measuredangle(BE, DF) = \measuredangle EGF \Rightarrow \measuredangle(AL, DF) = \measuredangle XGF$.

Step 21. $\measuredangle(AL, DF) = \measuredangle XGF$ and $\measuredangle(AL, FG) = \measuredangle DFX \Rightarrow \measuredangle FGX = \measuredangle XFG$.

Step 22. $\measuredangle FGX = \measuredangle XFG \Rightarrow FX = GX$.

Step 23. $AF = AG$ and $FX = GX \Rightarrow AX$ is perpendicular to FG.

Step 24. $AO = FO$ and $AO = GO \Rightarrow FO = GO$.

Step 25. $AF = AG$ and $FO = GO \Rightarrow AO$ is perpendicular to FG.

Step 26. AO is perpendicular to FG and AX is perpendicular to $FG \Rightarrow$ A, O, X are collinear

■

2.23 IMO 2016 P1

Original:

In convex pentagon ABCDE with $\measuredangle B > 90^o$, let F be a point on AC such that $\measuredangle FBC = 90^o$. It is given that FA = F B, DA = DC, EA = ED, and rays AC and AD trisect $\measuredangle BAE$. Let M be the midpoint of CF. Let X be the point such that AMXE is a parallelogram. Show that FX, EM, BD are concurrent.

Translated:

Let ABZ be a triangle. Define point F such that $AF = BF$, AF is the bisector of $\measuredangle BAZ$ and $\measuredangle ABF = \measuredangle FAB$. Define point C on line AF such that BC is perpendicular to BF. Define point D on line AZ such that $AD = CD$ and $\measuredangle ACD = \measuredangle DAC$. Define point E such that $AE = DE$, AD is the bisector of $\measuredangle CAE$ and $\measuredangle ADE = \measuredangle EAD$. Define point M as the midpoint of CF. Define point X such that AE is parallel to MX and AM is parallel to EX. Define point Y as the intersection of lines EM and FX. Prove that B, D, Y are collinear

Proof:

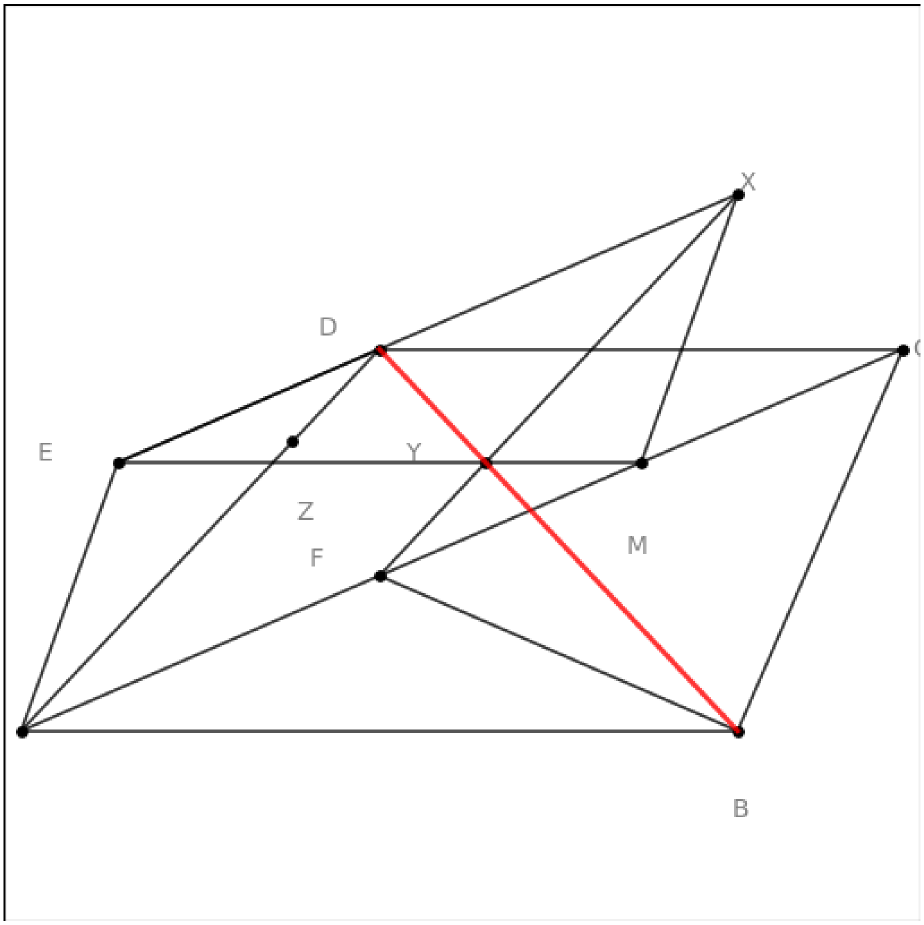

Figure 23: imo 2016 p1

Step 1. A, C, F are collinear, A, D, Z are collinear, AD is the bisector of $\measuredangle CAE$ and AE is parallel to $MX \Rightarrow \measuredangle(CF, DZ) = \measuredangle(DZ, MX)$.

Step 2. A, C, F are collinear, A, D, Z are collinear and $\measuredangle ACD = \measuredangle DAC \Rightarrow \measuredangle DCF = \measuredangle(CF, DZ)$.

Step 3. $\measuredangle DCF = \measuredangle(CF, DZ)$ and $\measuredangle(CF, DZ) = \measuredangle(DZ, MX) \Rightarrow \measuredangle CDZ = \measuredangle(CF, MX)$.

Step 4. A, C, F are collinear, A, D, Z are collinear, AF is the bisector of $\measuredangle BAZ$ and $\measuredangle ACD = \measuredangle DAC \Rightarrow \measuredangle(AB, CF) = \measuredangle DCF$.

Step 5. $\measuredangle(AB, CF) = \measuredangle DCF \Rightarrow AB$ is parallel to CD.

Step 6. A, C, F are collinear, AD is the bisector of $\measuredangle CAE$ and $\measuredangle ADE = \measuredangle EAD \Rightarrow \measuredangle(CF, DZ) = \measuredangle EDZ$.

Step 7. $\measuredangle(CF, DZ) = \measuredangle EDZ \Rightarrow CF$ is parallel to DE.

Step 8. A, C, F are collinear, A, D, Z are collinear, $\measuredangle CDZ = \measuredangle(CF, MX)$, AB is parallel to CD and AE is parallel to $MX \Rightarrow \measuredangle BAF = \measuredangle DAE$.

Step 9. A, C, F are collinear, $\measuredangle BAF = \measuredangle DAE$, $\measuredangle ADE = \measuredangle EAD$ and CF is parallel to $DE \Rightarrow \measuredangle BAF = \measuredangle CAD$.

Step 10. A, C, F are collinear, A, D, Z are collinear, AF is the bisector of $\measuredangle BAZ$, $\measuredangle ABF = \measuredangle FAB$ and $\measuredangle ACD = \measuredangle DAC \Rightarrow \measuredangle ABF = \measuredangle ACD$.

Step 11. $\measuredangle BAF = \measuredangle CAD$ and $\measuredangle ABF = \measuredangle ACD \Rightarrow \frac{AB}{AC} = \frac{BF}{CD}$.

Step 12. A, C, F are collinear, $\measuredangle BAF = \measuredangle DAE$, $\measuredangle ADE = \measuredangle EAD$ and CF is parallel to $DE \Rightarrow \measuredangle BAC = \measuredangle FAD$.

Step 13. $AD = CD$, $AF = BF$ and $\frac{AB}{AC} = \frac{BF}{CD} \Rightarrow \frac{AB}{AC} = \frac{AF}{AD}$.

Step 14. $\measuredangle BAC = \measuredangle FAD$ and $\frac{AB}{AC} = \frac{AF}{AD} \Rightarrow \measuredangle ABC = \measuredangle AFD$ and $\frac{BA}{BC} = \frac{FA}{FD}$.

Step 15. $\measuredangle ABC = \measuredangle AFD$, $\measuredangle ABF = \measuredangle FAB$ and AB is parallel to $CD \Rightarrow \measuredangle(BC, DF) = \measuredangle(BF, CD)$.

Step 16. $\measuredangle(BC, DF) = \measuredangle(BF, CD)$ and BC is perpendicular to $BF \Rightarrow \measuredangle BCD = \measuredangle BFD$ and $\measuredangle CBF = \measuredangle CDF$.

Step 17. $\measuredangle BCD = \measuredangle BFD$ and AB is parallel to $CD \Rightarrow \measuredangle ABC = \measuredangle DFB$.

Step 18. $AF = BF$ and $\frac{BA}{BC} = \frac{FA}{FD} \Rightarrow \frac{BA}{BC} = \frac{FB}{FD}$.

Step 19. $\measuredangle ABC = \measuredangle DFB$ and $\frac{BA}{BC} = \frac{FB}{FD} \Rightarrow \measuredangle ABD = \measuredangle(AC, BF)$, $\measuredangle(AC, DF) = \measuredangle CBD$ and $\measuredangle BAC = \measuredangle DBF$.

Step 20. A, C, F are collinear, A, D, Z are collinear, AF is the bisector of $\measuredangle BAZ$, AD is the bisector of $\measuredangle CAE$, $\measuredangle ABF = \measuredangle FAB$ and $\measuredangle ADE = \measuredangle EAD \Rightarrow \measuredangle ABF = \measuredangle ADE$.

Step 21. $\measuredangle BAF = \measuredangle DAE$ and $\measuredangle ABF = \measuredangle ADE \Rightarrow \frac{AB}{AD} = \frac{BF}{DE}$.

Step 22. A, C, F are collinear, A, D, Z are collinear, $\measuredangle CDZ = \measuredangle(CF, MX)$, AB is parallel to CD and AE is parallel to $MX \Rightarrow \measuredangle BAD = \measuredangle FAE$.

Step 23. $AE = DE$, $AF = BF$ and $\frac{AB}{AD} = \frac{BF}{DE} \Rightarrow \frac{AB}{AD} = \frac{AF}{AE}$.

Step 24. $\measuredangle BAD = \measuredangle FAE$ and $\frac{AB}{AD} = \frac{AF}{AE} \Rightarrow \measuredangle ABD = \measuredangle AFE$.

Step 25. C, F, M are collinear and $CM = FM \Rightarrow M$ is the midpoint of CF.

Step 26. M is the midpoint of CF and BC is perpendicular to $BF \Rightarrow BM = FM$ and $BM = CM$.

Step 27. $BM = FM \Rightarrow \measuredangle BFM = \measuredangle MBF$.

Step 28. A, C, F are collinear, A, D, Z are collinear, $\measuredangle BAF = \measuredangle DAE$ and AE is parallel to $MX \Rightarrow \measuredangle(AB, CF) = \measuredangle(DZ, MX)$.

Step 29. A, C, F are collinear, A, D, Z are collinear, AF is the bisector of $\measuredangle BAZ$ and $\measuredangle ABF = \measuredangle FAB \Rightarrow \measuredangle ABF = \measuredangle(DZ, CF)$.

Step 30. $\measuredangle(AB, CF) = \measuredangle(DZ, MX)$ and $\measuredangle ABF = \measuredangle(DZ, CF) \Rightarrow \measuredangle BFC = \measuredangle(CF, MX)$.

Step 31. A, C, F are collinear, C, F, M are collinear, $\measuredangle ABD = \measuredangle(AC, BF)$, $\measuredangle ABD = \measuredangle AFE$ and $\measuredangle BFM = \measuredangle MBF \Rightarrow \measuredangle AFE = \measuredangle FBM$.

Step 32. A, C, F are collinear, C, F, M are collinear, $\measuredangle BFC = \measuredangle(CF, MX)$ and AE is parallel to $MX \Rightarrow \measuredangle FAE = \measuredangle BFM$.

Step 33. $\measuredangle FAE = \measuredangle BFM$ and $\measuredangle AFE = \measuredangle FBM \Rightarrow \frac{AE}{FM} = \frac{FA}{FB}$.

Step 34. A, C, F are collinear, $\measuredangle ABC = \measuredangle AFD$ and AB is parallel to CD $\Rightarrow \measuredangle BCD = \measuredangle DFC$.

Step 35. A, C, F are collinear, $\measuredangle ABF = \measuredangle ACD$ and AB is parallel to CD $\Rightarrow \measuredangle(BF, CD) = \measuredangle DCF$.

Step 36. $\measuredangle(BF, CD) = \measuredangle DCF$ and $\measuredangle BCD = \measuredangle DFC \Rightarrow \measuredangle FBC = \measuredangle CDF$.

Step 37. $\measuredangle CBF = \measuredangle CDF$ and $\measuredangle FBC = \measuredangle CDF \Rightarrow CD$ is perpendicular to DF.

Step 38. M is the midpoint of CF and CD is perpendicular to $DF \Rightarrow DM = FM$.

Step 39. $\measuredangle ABC = \measuredangle AFD$ and $\measuredangle ABC = \measuredangle DFB \Rightarrow FD$ is the bisector of $\measuredangle AFB$.

Step 40. $AF = BF$ and FD is the bisector of $\measuredangle AFB \Rightarrow AD = BD$ and $\measuredangle DAF = \measuredangle FBD$.

Step 41. $AD = BD$ and $AD = CD \Rightarrow BD = CD$.

Step 42. $BD = CD$ and $BM = CM \Rightarrow BC$ is perpendicular to DM.

Step 43. $AE = DE$, $AF = BF$, $DM = FM$ and $\frac{AE}{FM} = \frac{FA}{FB} \Rightarrow DE = DM$.

Step 44. A, C, F are collinear, $\measuredangle DAF = \measuredangle FBD$, CF is parallel to DE, BC is perpendicular to BF and BC is perpendicular to $DM \Rightarrow \measuredangle ADM = \measuredangle EDB$.

Step 45. $AD = BD$, $DE = DM$ and $\measuredangle ADM = \measuredangle EDB \Rightarrow AM = BE$.

Step 46. AM is parallel to $EX \Rightarrow \measuredangle AME = \measuredangle XEM$.

Step 47. AE is parallel to $MX \Rightarrow \measuredangle AEM = \measuredangle XME$.

Step 48. $\measuredangle AEM = \measuredangle XME$ and $\measuredangle AME = \measuredangle XEM \Rightarrow AM = EX$.

Step 49. A, C, F are collinear, C, F, M are collinear and AM is parallel to $EX \Rightarrow EX$ is parallel to FM.

Step 50. E, M, Y are collinear, F, X, Y are collinear and EX is parallel to $FM \Rightarrow \frac{EX}{EY} = \frac{MF}{MY}$.

Step 51. A, C, F are collinear, $\measuredangle ABD = \measuredangle AFE$ and AB is parallel to CD $\Rightarrow \measuredangle BDC = \measuredangle EFC$.

Step 52. $\measuredangle BCD = \measuredangle DFC$ and $\measuredangle BDC = \measuredangle EFC \Rightarrow \measuredangle CBD = \measuredangle DFE$.

Step 53. A, C, F are collinear, $\measuredangle(AC, DF) = \measuredangle CBD$, $\measuredangle ABC = \measuredangle AFD$, $\measuredangle CBD = \measuredangle DFE$ and AB is parallel to $CD \Rightarrow \measuredangle BCD = \measuredangle EFD$.

Step 54. $\measuredangle BCD = \measuredangle EFD$ and CD is perpendicular to $DF \Rightarrow BC$ is perpendicular to EF.

Step 55. $\measuredangle(BF, CD) = \measuredangle DCF$ and $\measuredangle BDC = \measuredangle EFC \Rightarrow \measuredangle FBD = \measuredangle(CD, EF)$.

Step 56. A, C, F are collinear, $\measuredangle BAC = \measuredangle DBF$, $\measuredangle ABF = \measuredangle FAB$, $\measuredangle FBD = \measuredangle(CD, EF)$ and AB is parallel to $CD \Rightarrow \measuredangle(BF, CD) = \measuredangle(EF, CD)$.

Step 57. $\measuredangle(BF, CD) = \measuredangle(EF, CD) \Rightarrow BF$ is parallel to EF.

Step 58. BF is parallel to $EF \Rightarrow B, E, F$ are collinear.

Step 59. $AM = BE$, $AM = EX$, $DM = FM$ and $\frac{EX}{EY} = \frac{MF}{MY} \Rightarrow \frac{EB}{EY} = \frac{MD}{MY}$.

Step 60. B, E, F are collinear, E, M, Y are collinear, BC is perpendicular to DM and BC is perpendicular to $EF \Rightarrow \measuredangle BEY = \measuredangle DMY$.

Step 61. $\measuredangle BEY = \measuredangle DMY$ and $\frac{EB}{EY} = \frac{MD}{MY} \Rightarrow \measuredangle BYE = \measuredangle DYM$.

Step 62. E, M, Y are collinear and $\measuredangle BYE = \measuredangle DYM \Rightarrow BY$ is parallel to DY.

Step 63. BY is parallel to $DY \Rightarrow B, D, Y$ are collinear

■

2.24 IMO 2017 P4

Original:

Let R and S be different points on a circle Omega such that RS is not a diameter. Let l be the tangent line to Omega at R. Point T is such that S is the midpoint of RT. Point J is chosen on minor arc RS of Omega so that the circumcircle Gamma of triangle JST intersects l at two distinct points. Let A be the common point of Gamma and l closer to R. Line AJ meets Omega again at K. Prove that line KT is tangent to Gamma.

Translated:

Let R and S be any two distinct points. Define point T as the mirror of R through S. Define point O such that $OR = OS$. Let J be any point on circle (O, S). Define point O_1 as the circumcenter of triangle SJT. Define point A on circle (O_1, S) such that AR is perpendicular to OR. Define point K as the intersection of circle (O, S) and line AJ. Prove that KT is perpendicular to O_1T

Proof:

Step 1. $JO = OS$, $KO = OS$ and $OR = OS \Rightarrow J, K, R, S$ are cyclic.

Step 2. J, K, R, S are cyclic $\Rightarrow \measuredangle JKR = \measuredangle JSR$.

Step 3. $AO_1 = O_1S$, $JO_1 = O_1S$ and $O_1S = O_1T \Rightarrow A, J, S, T$ are cyclic.

Step 4. A, J, S, T are cyclic $\Rightarrow \measuredangle JAT = \measuredangle JST$ and $\measuredangle SAT = \measuredangle SJT$.

Step 5. $KO = OS$ and $OR = OS \Rightarrow O$ is the circumcenter of KRS.

Step 6. O is the circumcenter of KRS and AR is perpendicular to $OR \Rightarrow \measuredangle ARS = \measuredangle RKS$.

Step 7. A, J, K are collinear, R, S, T are collinear, $\measuredangle JAT = \measuredangle JST$ and $\measuredangle JKR = \measuredangle JSR \Rightarrow \measuredangle ATR = \measuredangle KRS$.

Step 8. R, S, T are collinear and $\measuredangle ARS = \measuredangle RKS \Rightarrow \measuredangle ART = \measuredangle RKS$.

Step 9. $\measuredangle ART = \measuredangle RKS$ and $\measuredangle ATR = \measuredangle KRS \Rightarrow \frac{AT}{RS} = \frac{RT}{RK}$.

Step 10. $RS = ST$ and $\frac{AT}{RS} = \frac{RT}{RK} \Rightarrow \frac{TA}{TS} = \frac{RT}{RK}$.

Step 11. A, J, K are collinear, R, S, T are collinear, $\measuredangle JAT = \measuredangle JST$ and $\measuredangle JKR = \measuredangle JSR \Rightarrow \measuredangle ATS = \measuredangle KRT$.

Step 12. $\measuredangle ATS = \measuredangle KRT$ and $\frac{TA}{TS} = \frac{RT}{RK} \Rightarrow \measuredangle TAS = \measuredangle KTR$.

Step 13. R, S, T are collinear, $\measuredangle SAT = \measuredangle SJT$ and $\measuredangle TAS = \measuredangle KTR \Rightarrow \measuredangle TJS = \measuredangle KTS$.

Step 14. $JO_1 = O_1S$ and $O_1S = O_1T \Rightarrow O_1$ is the circumcenter of JST.

Step 15. O_1 is the circumcenter of JST and $\measuredangle TJS = \measuredangle KTS \Rightarrow KT$ is perpendicular to O_1T

■

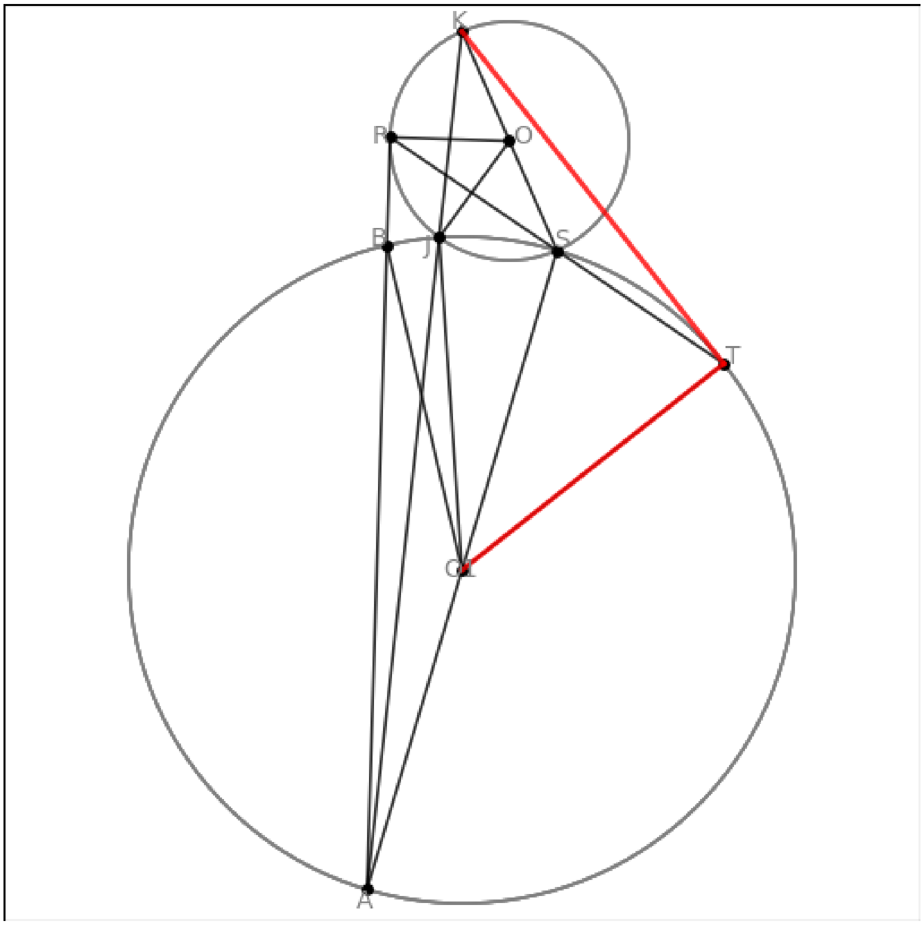

Figure 24: imo 2017 p4

2.25 IMO 2018 P1

Original:

Let Gamma be the circumcircle of triangle ABC. Points D and E lie on segments AB and AC, respectively, such that AD = AE. The perpendicular bisectors of BD and CE intersect the minor arcs AB and AC of Gamma at points F and G, respectively. Prove that the lines DE and F G are parallel

Translated:

Let ABC be a triangle. Define point O as the circumcenter of triangle CBA. Let D be any point on line AB. Define point E as the intersection of circle (A, D) and line AC. Define point F on circle (O, A) such that $\measuredangle BDF = \measuredangle FBD$. Define point G on circle (O, A) such that $\measuredangle CEG = \measuredangle GCE$. Prove that DE is parallel to FG

Proof:

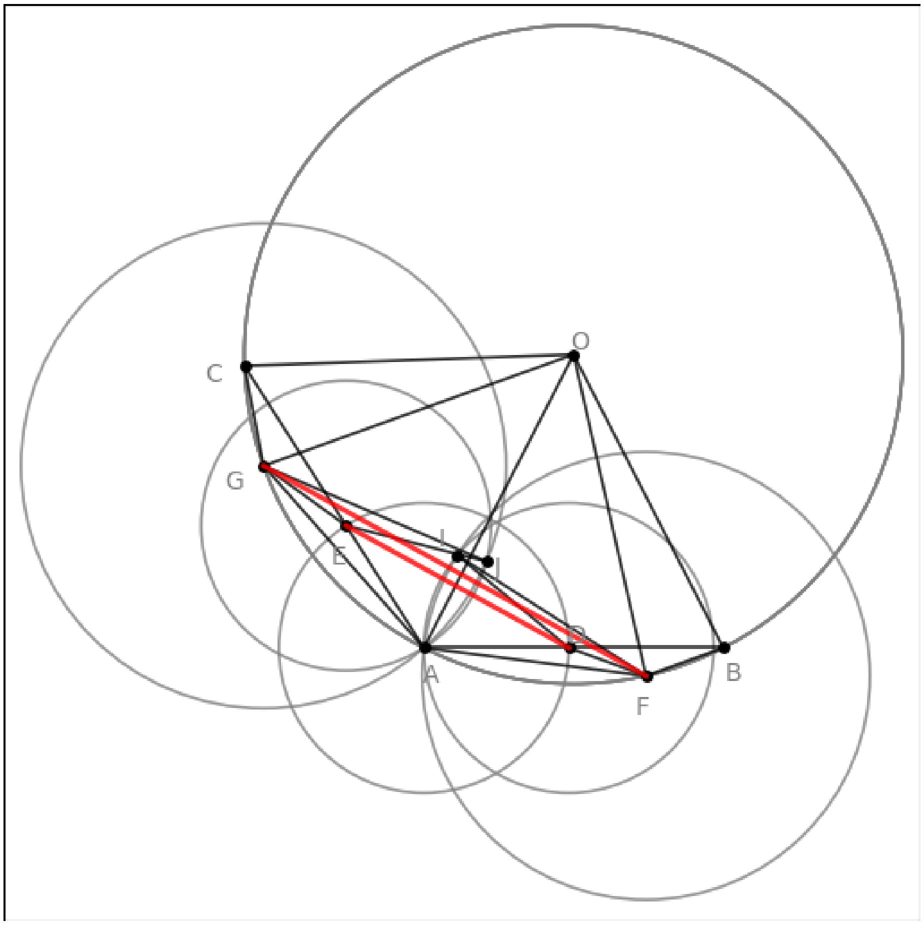

Figure 25: imo 2018 p1

Construct point I as the intersection of circles (D, A) and (F, A).
Construct point J as the intersection of circles (E, A) and (G, A).
Step 1. $AO = BO$, $AO = OF$ and $AO = OG \Rightarrow A, B, F, G$ are cyclic.
Step 2. $AO = BO$, $AO = OF$ and $BO = CO \Rightarrow A, B, C, F$ are cyclic.
Step 3. A, B, C, F are cyclic and A, B, F, G are cyclic $\Rightarrow B, C, F, G$ are cyclic.
Step 4. B, C, F, G are cyclic $\Rightarrow \measuredangle BCF = \measuredangle BGF$ and $\measuredangle BFC = \measuredangle BGC$.
Step 5. A, B, F, G are cyclic $\Rightarrow \measuredangle AFB = \measuredangle AGB$.
Step 6. A, B, C, F are cyclic $\Rightarrow \measuredangle BAF = \measuredangle BCF$ and $\measuredangle BAC = \measuredangle BFC$.
Step 7. $AO = OF$ and $AO = OG \Rightarrow OF = OG$.
Step 8. $OF = OG \Rightarrow \measuredangle OFG = \measuredangle FGO$.
Step 9. $AD = DI$ and $AF = FI \Rightarrow AI$ is perpendicular to DF.
Step 10. $AO = OF \Rightarrow \measuredangle OAF = \measuredangle AFO$.
Step 11. $AO = OG \Rightarrow \measuredangle OAG = \measuredangle AGO$.

Step 12. A, B, D are collinear and $\angle BDF = \angle FBD \Rightarrow \angle(AB, DF) = \angle FBA$.

Step 13. $\angle BAF = \angle BCF$, $\angle(AB, DF) = \angle FBA$, $\angle OAF = \angle AFO$, $\angle OAG = \angle AGO$, $\angle BCF = \angle BGF$, $\angle AFB = \angle AGB$, $\angle OFG = \angle FGO$ and AI is perpendicular to $DF \Rightarrow$ by angle chasing: $\angle AFO = \angle IAB$.

Step 14. $AD = DI \Rightarrow \angle DAI = \angle AID$.

Step 15. A, B, D are collinear and $\angle DAI = \angle AID \Rightarrow \angle BAI = \angle AID$.

Step 16. $\angle BAF = \angle BCF$, $\angle BAI = \angle AID$, $\angle(AB, DF) = \angle FBA$, $\angle OAF = \angle AFO$, $\angle OAG = \angle AGO$, $\angle BCF = \angle BGF$, $\angle AFB = \angle AGB$, $\angle OFG = \angle FGO$ and AI is perpendicular to $DF \Rightarrow$ by angle chasing: $\angle FAO = \angle AID$.

Step 17. A, B, D are collinear and $\angle AFO = \angle IAB \Rightarrow \angle AFO = \angle IAD$.

Step 18. $\angle FAO = \angle AID$ and $\angle AFO = \angle IAD \Rightarrow \frac{AD}{OF} = \frac{AI}{AF}$.

Step 19. $AO = BO$, $AD = AE$ and $AE = EJ \Rightarrow$ by ratio chasing: $\frac{OA}{OB} = \frac{AD}{EJ}$.

Step 20. $AE = EJ$ and $AG = GJ \Rightarrow AJ$ is perpendicular to EG.

Step 21. A, C, E are collinear and $\angle CEG = \angle GCE \Rightarrow \angle(AC, EG) = \angle GCA$.

Step 22. $\angle BAC = \angle BFC$, $\angle BAF = \angle BCF$, $\angle(AC, EG) = \angle GCA$, $\angle OAF = \angle AFO$, $\angle OAG = \angle AGO$, $\angle BCF = \angle BGF$, $\angle BFC = \angle BGC$, $\angle OFG = \angle FGO$ and AJ is perpendicular to $EG \Rightarrow$ by angle chasing: $\angle AGO = \angle JAC$.

Step 23. $AE = EJ \Rightarrow \angle EAJ = \angle AJE$.

Step 24. A, C, E are collinear and $\angle EAJ = \angle AJE \Rightarrow \angle CAJ = \angle AJE$.

Step 25. $\angle BAC = \angle BFC$, $\angle BAF = \angle BCF$, $\angle CAJ = \angle AJE$, $\angle(AC, EG) = \angle GCA$, $\angle OAF = \angle AFO$, $\angle OAG = \angle AGO$, $\angle BCF = \angle BGF$, $\angle BFC = \angle BGC$, $\angle OFG = \angle FGO$ and AJ is perpendicular to $EG \Rightarrow$ by angle chasing: $\angle GAO = \angle AJE$.

Step 26. A, C, E are collinear and $\angle AGO = \angle JAC \Rightarrow \angle AGO = \angle JAE$.

Step 27. $\angle GAO = \angle AJE$ and $\angle AGO = \angle JAE \Rightarrow \frac{AE}{OG} = \frac{AJ}{AG}$.

Step 28. $AO = BO$, $AE = EJ$, $OF = OG$, $\frac{AD}{OF} = \frac{AI}{AF}$, $\frac{AE}{OG} = \frac{AJ}{AG}$ and $\frac{OA}{OB} = \frac{AD}{EJ} \Rightarrow \frac{AF}{AI} = \frac{AG}{AJ}$.

Step 29. $AF = FI$, $AG = GJ$ and $\frac{AF}{AI} = \frac{AG}{AJ} \Rightarrow \frac{IA}{IF} = \frac{JA}{JG}$.

Step 30. $\frac{AF}{AI} = \frac{AG}{AJ}$ and $\frac{IA}{IF} = \frac{JA}{JG} \Rightarrow \angle AGJ = \angle IFA$.

Step 31. $AD = AE \Rightarrow \angle ADE = \angle DEA$.

Step 32. $AF = FI \Rightarrow \angle FAI = \angle AIF$.

Step 33. $AG = GJ \Rightarrow \angle GAJ = \angle AJG$.

Step 34. A, B, D are collinear, A, C, E are collinear and $\angle ADE = \angle DEA \Rightarrow \angle(AB, DE) = \angle(DE, AC)$.

Step 35. $\angle BAC = \angle BFC$, $\angle BAF = \angle BCF$, $\angle(AB, DE) = \angle(DE, AC)$, $\angle(AB, DF) = \angle FBA$, $\angle(AC, EG) = \angle GCA$, $\angle FAI = \angle AIF$, $\angle GAJ = \angle AJG$, $\angle BCF = \angle BGF$, $\angle AFB = \angle AGB$, $\angle BFC = \angle BGC$, $\angle AGJ = \angle IFA$, AI is perpendicular to DF and AJ is perpendicular to $EG \Rightarrow$ by angle chasing: DE is parallel to FG

■

2.26 IMO 2019 P2

Original:

In triangle ABC, point A1 lies on side BC and point B1 lies on side AC. Let P and Q be points on segments AA1 and BB1, respectively, such that P Q is parallel to AB. Let P1 be a point on ray PB1 beyond B1 such that angle PP1C = angle BAC. Similarly, let Q1 be a point on ray QA1 beyond A1 such that angle CQ1Q = angle CBA. Prove that points P, Q, P1, and Q1 are concyclic.

Translated:

Let ABC be a triangle. Let A_1 be any point on line BC. Let B_1 be any point on line AC. Let P be any point on line AA_1. Define point Q on line BB_1 such that AB is parallel to PQ. Define point P_1 on line B_1P such that $\measuredangle BAC = \measuredangle PP_1C$. Define point Q_1 on line A_1Q such that $\measuredangle ABC = \measuredangle QQ_1C$. Prove that P, P_1, Q, Q_1 are cyclic

Proof:

Construct point O as the circumcenter of triangle CBA.

Construct point A_2 as the intersection of circle (O, A) and line AA_1.

Construct point B_2 as the intersection of circle (O, B) and line BB_1.

Step 1. $AO = A_2O$, $AO = BO$ and $BO = CO \Rightarrow A, A_2, B, C$ are cyclic.

Step 2. A, A_2, B, C are cyclic $\Rightarrow \measuredangle AA_2C = \measuredangle ABC$.

Step 3. A, A_1, A_2 are collinear, A_1, Q, Q_1 are collinear, $\measuredangle AA_2C = \measuredangle ABC$ and $\measuredangle ABC = \measuredangle QQ_1C \Rightarrow \measuredangle A_1A_2C = \measuredangle A_1Q_1C$.

Step 4. $\measuredangle A_1A_2C = \measuredangle A_1Q_1C \Rightarrow A_1, A_2, C, Q_1$ are cyclic.

Step 5. A_1, A_2, C, Q_1 are cyclic $\Rightarrow \measuredangle A_1A_2Q_1 = \measuredangle A_1CQ_1$.

Step 6. A, A_1, A_2 are collinear, A, A_1, P are collinear, A_1, B, C are collinear, $\measuredangle A_1A_2Q_1 = \measuredangle A_1CQ_1$, $\measuredangle ABC = \measuredangle QQ_1C$ and AB is parallel to $PQ \Rightarrow \measuredangle PA_2Q_1 = \measuredangle PQQ_1$.

Step 7. $\measuredangle PA_2Q_1 = \measuredangle PQQ_1 \Rightarrow A_2, P, Q, Q_1$ are cyclic.

Step 8. $AO = BO$, $BO = B_2O$ and $BO = CO \Rightarrow A, B, B_2, C$ are cyclic.

Step 9. A, A_2, B, C are cyclic and A, B, B_2, C are cyclic $\Rightarrow A, A_2, B, B_2$ are cyclic.

Step 10. A, A_2, B, B_2 are cyclic $\Rightarrow \measuredangle AA_2B_2 = \measuredangle ABB_2$.

Step 11. A, A_1, A_2 are collinear, A, A_1, P are collinear, B, B_1, B_2 are collinear, B, B_1, Q are collinear, $\measuredangle AA_2B_2 = \measuredangle ABB_2$ and AB is parallel to $PQ \Rightarrow \measuredangle B_2A_2P = \measuredangle B_2QP$.

Step 12. $\measuredangle B_2A_2P = \measuredangle B_2QP \Rightarrow A_2, B_2, P, Q$ are cyclic.

Step 13. A, B, B_2, C are cyclic $\Rightarrow \measuredangle ABB_2 = \measuredangle ACB_2$.

Step 14. B, B_1, B_2 are collinear, B_1, P, P_1 are collinear, $\measuredangle BAC = \measuredangle PP_1C$ and $\measuredangle ABB_2 = \measuredangle ACB_2 \Rightarrow \measuredangle B_1B_2C = \measuredangle B_1P_1C$.

Step 15. $\measuredangle B_1B_2C = \measuredangle B_1P_1C \Rightarrow B_1, B_2, C, P_1$ are cyclic.

Step 16. B_1, B_2, C, P_1 are cyclic $\Rightarrow \measuredangle B_1B_2P_1 = \measuredangle B_1CP_1$.

Step 17. A, B_1, C are collinear, B, B_1, B_2 are collinear, B, B_1, Q are collinear, $\measuredangle BAC = \measuredangle PP_1C$, $\measuredangle B_1B_2P_1 = \measuredangle B_1CP_1$ and AB is parallel to $PQ \Rightarrow \measuredangle P_1B_2Q = \measuredangle P_1PQ$.

Step 18. $\measuredangle P_1B_2Q = \measuredangle P_1PQ \Rightarrow B_2, P, P_1, Q$ are cyclic.

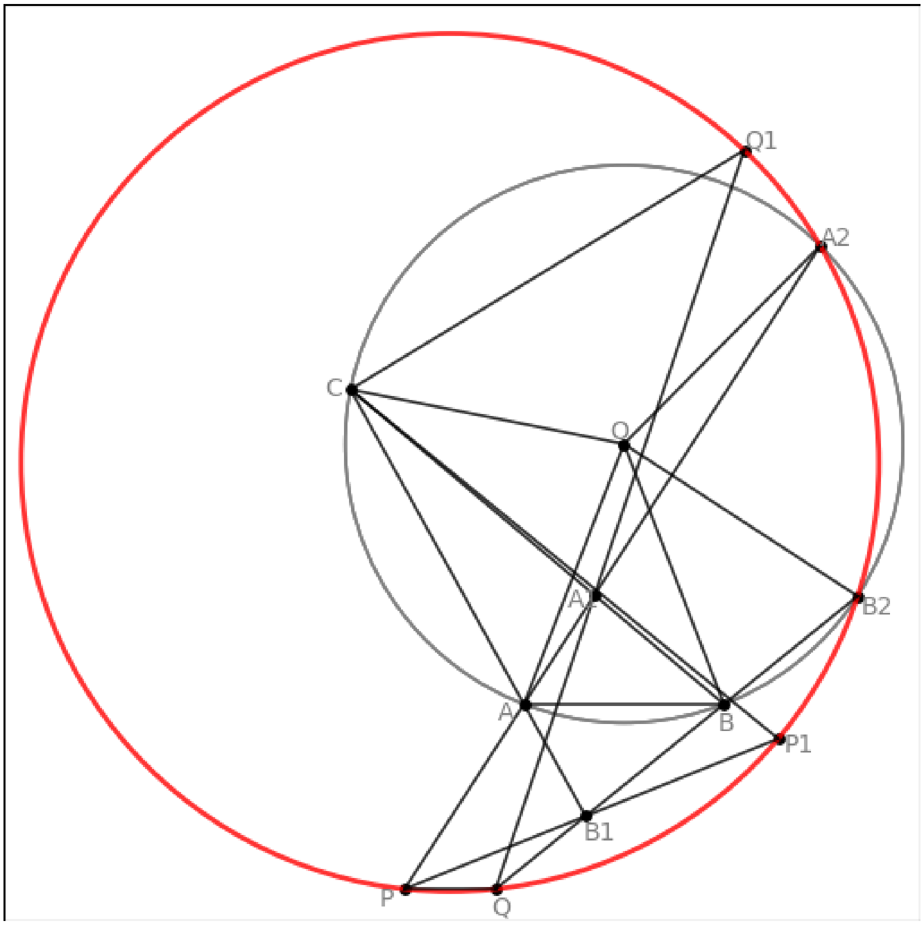

Figure 26: imo 2019 p2

Step 19. A_2, B_2, P, Q are cyclic, A_2, P, Q, Q_1 are cyclic and B_2, P, P_1, Q are cyclic $\Rightarrow P, P_1, Q, Q_1$ are cyclic

■

2.27 IMO 2019 P6

Original:

Let I be the incentre of triangle ABC with $AB \neq AC$. The incircle W of ABC is tangent to sides BC, CA, and AB at D, E, and F, respectively. The line through D perpendicular to EF meets W again at R. Line AR meets W again at P. The circumcircles of triangles P CE and P BF meet again at Q. Prove that lines DI and PQ meet on the line through A perpendicular to AI.

Translated:

Let ABC be a triangle. Define point I such that AI is the bisector of $\measuredangle BAC$ and CI is the bisector of $\measuredangle ACB$. Define point D as the foot of I on line BC. Define point E as the foot of I on line AC. Define point F as the foot of I on line AB. Define point R on circle (I, D) such that DR is perpendicular to EF. Define point P as the intersection of circle (I, D) and line AR. Define point O_1 as the circumcenter of triangle ECP. Define point O_2 as the circumcenter of triangle FBP. Define point Q as the intersection of circles (O_1, P) and (O_2, P). Define point T as the intersection of lines DI and PQ. Prove that AI is perpendicular to AT

Proof:

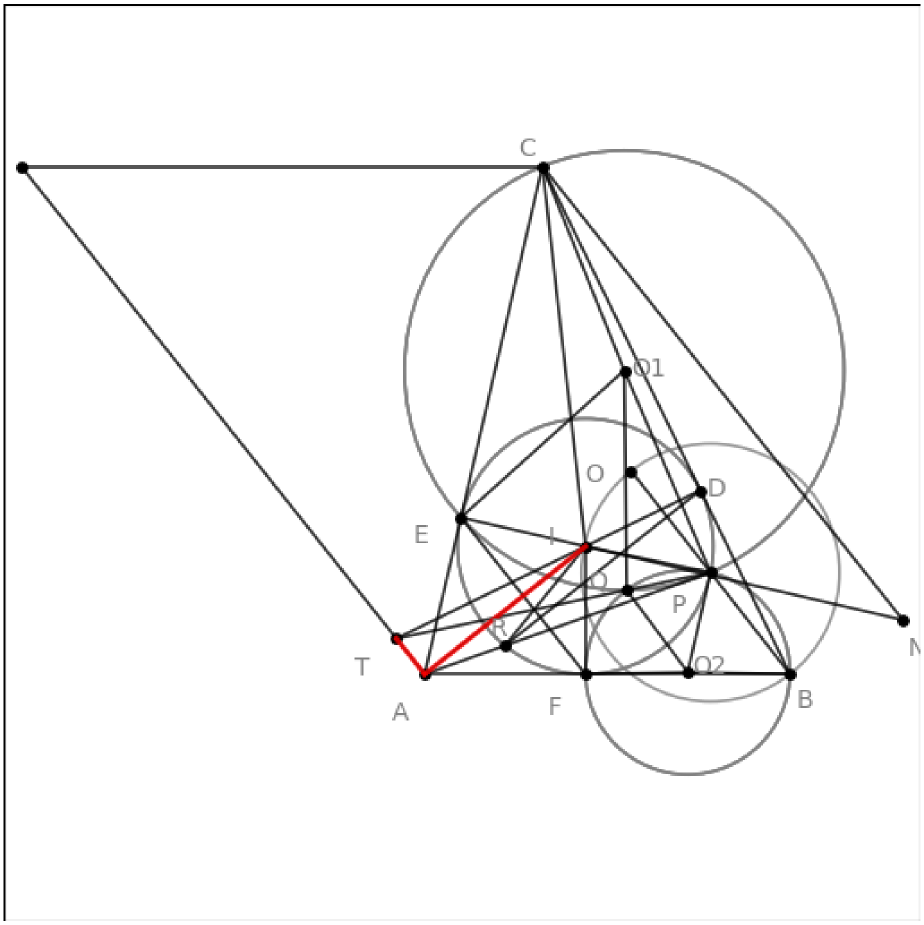

Figure 27: imo 2019 p6

Construct point N as the orthocenter of triangle CAI.

Construct point G such that AB is parallel to CG and AG is parallel to CN.

Step 1. A, C, E are collinear, B, C, D are collinear, AC is perpendicular to EI and BC is perpendicular to $DI \Rightarrow \measuredangle CDI = \measuredangle IEC$.

Step 2. A, C, E are collinear, B, C, D are collinear and CI is the bisector of $\measuredangle ACB \Rightarrow CI$ is the bisector of $\measuredangle DCE$.

Step 3. CI is the bisector of $\measuredangle DCE$ and $\measuredangle CDI = \measuredangle IEC \Rightarrow DI = EI$ and $CD = CE$.

Step 4. $DI = EI$ and $DI = IR \Rightarrow I$ is the circumcenter of DER.

Step 5. B, C, D are collinear and BC is perpendicular to $DI \Rightarrow CD$ is perpendicular to DI.

Step 6. I is the circumcenter of DER and CD is perpendicular to $DI \Rightarrow \measuredangle CDR = \measuredangle DER$.

Step 7. D, I, T are collinear and BC is perpendicular to $DI \Rightarrow BC$ is perpendicular to IT.

Step 8. BC is perpendicular to IT and DR is perpendicular to $EF \Rightarrow \measuredangle (BC, EF) = \measuredangle (IT, DR)$ and $\measuredangle (BC, DR) = \measuredangle (IT, EF)$.

Step 9. $DI = IR \Rightarrow \measuredangle IDR = \measuredangle DRI$.

Step 10. $\measuredangle (BC, EF) = \measuredangle (IT, DR) \Rightarrow \measuredangle (BC, IT) = \measuredangle (EF, DR)$.

Step 11. D, I, T are collinear and $\measuredangle IDR = \measuredangle DRI \Rightarrow \measuredangle (DR, IT) = \measuredangle IRD$.

Step 12. $\measuredangle (BC, IT) = \measuredangle (EF, DR)$ and $\measuredangle (DR, IT) = \measuredangle IRD \Rightarrow \measuredangle (BC, DR) = \measuredangle (EF, IR)$.

Step 13. A, B, F are collinear, A, C, E are collinear, AB is perpendicular to FI and AC is perpendicular to $EI \Rightarrow \measuredangle AEI = \measuredangle IFA$.

Step 14. A, B, F are collinear, A, C, E are collinear and AI is the bisector of $\measuredangle BAC \Rightarrow AI$ is the bisector of $\measuredangle EAF$.

Step 15. AI is the bisector of $\measuredangle EAF$ and $\measuredangle AEI = \measuredangle IFA \Rightarrow EI = FI$, $AE = AF$ and $\frac{EA}{EI} = \frac{FA}{FI}$.

Step 16. $AE = AF$ and $EI = FI \Rightarrow AI$ is perpendicular to EF.

Step 17. $DI = EI$ and $EI = FI \Rightarrow I$ is the circumcenter of DEF.

Step 18. A, B, F are collinear and AB is perpendicular to $FI \Rightarrow AF$ is perpendicular to FI.

Step 19. I is the circumcenter of DEF and AF is perpendicular to $FI \Rightarrow \measuredangle AFD = \measuredangle FED$.

Step 20. B, C, D are collinear, $\measuredangle (BC, DR) = \measuredangle (EF, IR)$, $\measuredangle CDR = \measuredangle DER$, AI is perpendicular to CN and AI is perpendicular to $EF \Rightarrow \measuredangle (CN, IR) = \measuredangle DER$.

Step 21. $\measuredangle AFD = \measuredangle FED$, AI is perpendicular to CN and AI is perpendicular to $EF \Rightarrow \measuredangle (AF, CN) = \measuredangle FDE$.

Step 22. $\measuredangle (AF, CN) = \measuredangle FDE$ and $\measuredangle (CN, IR) = \measuredangle DER \Rightarrow \measuredangle (AF, IR) = \measuredangle (DF, ER)$.

Step 23. $DI = EI$, $DI = IR$ and $EI = FI \Rightarrow I$ is the circumcenter of EFR.

Step 24. I is the circumcenter of EFR and AF is perpendicular to $FI \Rightarrow \measuredangle AFR = \measuredangle FER$.

Step 25. A, B, F are collinear, A, C, E are collinear, AB is perpendicular to FI and AC is perpendicular to $EI \Rightarrow \measuredangle AEI = \measuredangle AFI$.

Step 26. $\measuredangle AEI = \measuredangle AFI \Rightarrow A, E, F, I$ are cyclic.

Step 27. A, E, F, I are cyclic $\Rightarrow \measuredangle AFE = \measuredangle AIE$ and $\measuredangle AEF = \measuredangle AIF$.

Step 28. $DI = EI$, $DI = IR$ and $EI = FI \Rightarrow I$ is the circumcenter of DFR.

Step 29. I is the circumcenter of DFR and CD is perpendicular to $DI \Rightarrow \measuredangle CDF = \measuredangle DRF$.

Step 30. $\measuredangle AFE = \measuredangle AIE$, $\measuredangle AFR = \measuredangle FER$, AC is perpendicular to EI and AC is perpendicular to $IN \Rightarrow \measuredangle(AI, EN) = \measuredangle FRE$.

Step 31. B, C, D are collinear, $\measuredangle CDF = \measuredangle DRF$, AI is perpendicular to EF and DR is perpendicular to $EF \Rightarrow \measuredangle(AI, BC) = \measuredangle RFD$.

Step 32. $\measuredangle(AI, BC) = \measuredangle RFD$ and $\measuredangle(AI, EN) = \measuredangle FRE \Rightarrow \measuredangle(BC, EN) = \measuredangle(DF, ER)$.

Step 33. A, B, F are collinear and $\measuredangle(AF, IR) = \measuredangle(DF, ER) \Rightarrow \measuredangle BFD = \measuredangle IRE$.

Step 34. B, C, D are collinear, $\measuredangle(BC, EN) = \measuredangle(DF, ER)$, AC is perpendicular to EI and AC is perpendicular to $IN \Rightarrow \measuredangle BDF = \measuredangle IER$.

Step 35. $\measuredangle BDF = \measuredangle IER$ and $\measuredangle BFD = \measuredangle IRE \Rightarrow \frac{FB}{FD} = \frac{RI}{RE}$.

Step 36. A, C, E are collinear and AC is perpendicular to $EI \Rightarrow AE$ is perpendicular to EI.

Step 37. I is the circumcenter of DEF and AE is perpendicular to $EI \Rightarrow \measuredangle AEF = \measuredangle EDF$.

Step 38. B, C, D are collinear, $\measuredangle(BC, DR) = \measuredangle(EF, IR)$, $\measuredangle CDF = \measuredangle DRF$, AI is perpendicular to CN and AI is perpendicular to $EF \Rightarrow \measuredangle(CN, IR) = \measuredangle DFR$.

Step 39. A, C, E are collinear, $\measuredangle AEF = \measuredangle EDF$, AI is perpendicular to CN and AI is perpendicular to $EF \Rightarrow \measuredangle ACN = \measuredangle EDF$.

Step 40. $\measuredangle ACN = \measuredangle EDF$ and $\measuredangle(CN, IR) = \measuredangle DFR \Rightarrow \measuredangle(AC, DE) = \measuredangle IRF$.

Step 41. I is the circumcenter of EFR and AE is perpendicular to $EI \Rightarrow \measuredangle AEF = \measuredangle ERF$.

Step 42. $\measuredangle AEF = \measuredangle AIF$ and $\measuredangle AEF = \measuredangle ERF \Rightarrow \measuredangle AIF = \measuredangle ERF$.

Step 43. B, C, D are collinear, $\measuredangle CDR = \measuredangle DER$, AI is perpendicular to EF and DR is perpendicular to $EF \Rightarrow \measuredangle(AI, BC) = \measuredangle RED$.

Step 44. $\measuredangle(AI, BC) = \measuredangle RED$ and $\measuredangle AIF = \measuredangle ERF \Rightarrow \measuredangle(BC, DE) = \measuredangle IFR$.

Step 45. A, C, E are collinear and $\measuredangle(AC, DE) = \measuredangle IRF \Rightarrow \measuredangle CED = \measuredangle IRF$.

Step 46. B, C, D are collinear and $\measuredangle(BC, DE) = \measuredangle IFR \Rightarrow \measuredangle CDE = \measuredangle IFR$.

Step 47. $\measuredangle CDE = \measuredangle IFR$ and $\measuredangle CED = \measuredangle IRF \Rightarrow \frac{DC}{DE} = \frac{FI}{FR}$.

Step 48. $CD = CE$ and $DI = EI \Rightarrow CI$ is perpendicular to DE.

Step 49. AC is perpendicular to EI, AC is perpendicular to IN and CI is perpendicular to $DE \Rightarrow \measuredangle(AC, EN) = \measuredangle(DE, CI)$.

Step 50. $\measuredangle(AC, EN) = \measuredangle(DE, CI)$ and $\measuredangle ACN = \measuredangle EDF \Rightarrow \measuredangle CNE = \measuredangle(DF, CI)$.

Step 51. AC is perpendicular to EI and AC is perpendicular to $IN \Rightarrow AC$ is perpendicular to EN.

Step 52. AC is perpendicular to EN and AI is perpendicular to $CN \Rightarrow \measuredangle ACN = \measuredangle(EN, AI)$.

Step 53. A, C, E are collinear, $\measuredangle ACN = \measuredangle(EN, AI)$, AC is perpendicular to EI and AC is perpendicular to $IN \Rightarrow \measuredangle AIE = \measuredangle NCE$.

Step 54. A, C, E are collinear, AC is perpendicular to EI and AC is perpendicular to $IN \Rightarrow \measuredangle AEI = \measuredangle NEC$.

Step 55. $\measuredangle AEI = \measuredangle NEC$ and $\measuredangle AIE = \measuredangle NCE \Rightarrow \frac{EA}{EN} = \frac{EI}{EC}$, $\frac{EA}{EI} = \frac{EN}{EC}$ and $\frac{AI}{AE} = \frac{NC}{NE}$.

Step 56. A, C, E are collinear, AC is perpendicular to EI and AC is perpendicular to $IN \Rightarrow \measuredangle AEN = \measuredangle IEC$.

Step 57. $\measuredangle AEN = \measuredangle IEC$ and $\frac{EA}{EN} = \frac{EI}{EC} \Rightarrow \measuredangle NAE = \measuredangle CIE$ and $\measuredangle ANE = \measuredangle ICE$.

Step 58. A, C, E are collinear, $\measuredangle NAE = \measuredangle CIE$, $\measuredangle CNE = \measuredangle(DF, CI)$, AC is perpendicular to EI, AC is perpendicular to IN, AI is perpendicular to CN and AI is perpendicular to $EF \Rightarrow \measuredangle CAN = \measuredangle EFD$.

Step 59. $\measuredangle ACN = \measuredangle EDF$ and $\measuredangle CAN = \measuredangle EFD \Rightarrow \frac{CA}{CN} = \frac{DF}{DE}$.

Step 60. AC is perpendicular to EI, AC is perpendicular to IN and AI is perpendicular to $CN \Rightarrow \measuredangle(AC, EN) = \measuredangle(AI, CN)$.

Step 61. A, B, F are collinear and AI is the bisector of $\measuredangle BAC \Rightarrow AI$ is the bisector of $\measuredangle CAF$.

Step 62. AI is the bisector of $\measuredangle CAF$ and $\measuredangle(AC, EN) = \measuredangle(AI, CN) \Rightarrow \measuredangle(AF, CN) = \measuredangle(AI, EN)$.

Step 63. A, B, F are collinear, $\measuredangle(AF, CN) = \measuredangle(AI, EN)$, $\measuredangle ACN = \measuredangle(EN, AI)$, AB is parallel to CG and AG is parallel to $CN \Rightarrow \measuredangle CAG = \measuredangle AGC$.

Step 64. $\measuredangle CAG = \measuredangle AGC \Rightarrow AC = CG$.

Step 65. $DI = EI$, $DI = IP$ and $EI = FI \Rightarrow FI = IP$.

Step 66. $BO_2 = FO_2$ and $BO_2 = PO_2 \Rightarrow FO_2 = PO_2$.

Step 67. $FI = IP$ and $FO_2 = PO_2 \Rightarrow \measuredangle IFO_2 = \measuredangle O_2PI$.

Step 68. $FI = IP$ and $FO_2 = PO_2 \Rightarrow FP$ is perpendicular to IO_2.

Step 69. $BO_2 = FO_2$, $BO_2 = PO_2$ and $PO_2 = O_2Q \Rightarrow B, F, P, Q$ are cyclic.

Step 70. B, F, P, Q are cyclic $\Rightarrow \measuredangle FBQ = \measuredangle FPQ$ and $\measuredangle BFQ = \measuredangle BPQ$.

Step 71. $BO_2 = FO_2$, $BO_2 = PO_2$ and $PO_2 = O_2Q \Rightarrow FO_2 = O_2Q$.

Step 72. $FO_2 = O_2Q \Rightarrow \measuredangle O_2FQ = \measuredangle FQO_2$.

Step 73. $BO_2 = PO_2$ and $PO_2 = O_2Q \Rightarrow BO_2 = O_2Q$.

Step 74. $BO_2 = O_2Q \Rightarrow \measuredangle O_2BQ = \measuredangle BQO_2$.

Step 75. $FO_2 = PO_2 \Rightarrow \measuredangle FPO_2 = \measuredangle O_2FP$.

Step 76. $PO_2 = O_2Q \Rightarrow \measuredangle O_2PQ = \measuredangle PQO_2$.

Step 77. A, B, F are collinear and $\measuredangle FBQ = \measuredangle FPQ \Rightarrow \measuredangle ABQ = \measuredangle FPQ$.

Step 78. A, B, F are collinear and $\measuredangle BFQ = \measuredangle BPQ \Rightarrow \measuredangle(AB, FQ) = \measuredangle BPQ$.

Step 79. $\measuredangle(AB, FQ) = \measuredangle BPQ$, $\measuredangle ABQ = \measuredangle FPQ$, $\measuredangle O_2BQ = \measuredangle BQO_2$, $\measuredangle O_2FQ = \measuredangle FQO_2$, $\measuredangle FPO_2 = \measuredangle O_2FP$, $\measuredangle O_2PQ = \measuredangle PQO_2$ and AB is perpendicular to $FI \Rightarrow$ by angle chasing: $\measuredangle BPF = \measuredangle(BO_2, FI)$ and $\measuredangle O_2BP = \measuredangle IFP$.

Step 80. $\measuredangle ABQ = \measuredangle FPQ$, $\measuredangle O_2BQ = \measuredangle BQO_2$, $\measuredangle FPO_2 = \measuredangle O_2FP$, $\measuredangle O_2PQ = \measuredangle PQO_2$ and AB is perpendicular to $FI \Rightarrow$ by angle chasing: $\measuredangle(BO_2, FI) = \measuredangle IFO_2$.

Step 81. $FI = IP \Rightarrow \measuredangle IFP = \measuredangle FPI$.

Step 82. A, B, F are collinear, AB is perpendicular to FI and FP is perpendicular to $IO_2 \Rightarrow \measuredangle AFI = \measuredangle(IO_2, FP)$.

Step 83. $\measuredangle AFI = \measuredangle(IO_2, FP)$ and $\measuredangle IFP = \measuredangle FPI \Rightarrow \measuredangle AFP = \measuredangle O_2IP$.

Step 84. $\measuredangle(BO_2, FI) = \measuredangle IFO_2$, $\measuredangle IFO_2 = \measuredangle O_2PI$ and $\measuredangle BPF = \measuredangle(BO_2, FI)$ $\Rightarrow \measuredangle BPF = \measuredangle O_2PI$.

Step 85. A, B, F are collinear and $\measuredangle AFP = \measuredangle O_2IP \Rightarrow \measuredangle BFP = \measuredangle O_2IP$.

Step 86. $\measuredangle BFP = \measuredangle O_2IP$ and $\measuredangle BPF = \measuredangle O_2PI \Rightarrow \frac{BF}{BP} = \frac{O_2I}{O_2P}$.

Step 87. $\measuredangle(AB, FQ) = \measuredangle BPQ$, $\measuredangle O_2FQ = \measuredangle FQO_2$, $\measuredangle FPO_2 = \measuredangle O_2FP$, $\measuredangle O_2PQ = \measuredangle PQO_2$ and AB is perpendicular to $FI \Rightarrow$ by angle chasing: $\measuredangle BPO_2 = \measuredangle IFP$.

Step 88. $\measuredangle IFP = \measuredangle FPI$ and $\measuredangle BPO_2 = \measuredangle IFP \Rightarrow \measuredangle BPO_2 = \measuredangle FPI$.

Step 89. $\measuredangle O_2BP = \measuredangle IFP$ and $\measuredangle BPO_2 = \measuredangle FPI \Rightarrow \frac{PB}{PO_2} = \frac{PF}{PI}$.

Step 90. $DI = EI$ and $DI = IP \Rightarrow EI = IP$.

Step 91. $CO_1 = EO_1$ and $CO_1 = PO_1 \Rightarrow EO_1 = PO_1$.

Step 92. $EI = IP$ and $EO_1 = PO_1 \Rightarrow \measuredangle IEO_1 = \measuredangle O_1PI$.

Step 93. $EI = IP$ and $EO_1 = PO_1 \Rightarrow EP$ is perpendicular to IO_1.

Step 94. $CO_1 = EO_1$, $CO_1 = PO_1$ and $PO_1 = O_1Q \Rightarrow C, E, P, Q$ are cyclic.

Step 95. C, E, P, Q are cyclic $\Rightarrow \measuredangle ECQ = \measuredangle EPQ$.

Step 96. $CO_1 = PO_1$ and $PO_1 = O_1Q \Rightarrow CO_1 = O_1Q$.

Step 97. $CO_1 = O_1Q \Rightarrow \measuredangle O_1CQ = \measuredangle CQO_1$.

Step 98. $CO_1 = EO_1 \Rightarrow \measuredangle CEO_1 = \measuredangle O_1CE$.

Step 99. $CO_1 = PO_1 \Rightarrow \measuredangle CPO_1 = \measuredangle O_1CP$.

Step 100. $PO_1 = O_1Q \Rightarrow \measuredangle O_1PQ = \measuredangle PQO_1$.

Step 101. A, C, E are collinear and $\measuredangle ECQ = \measuredangle EPQ \Rightarrow \measuredangle ACQ = \measuredangle EPQ$.

Step 102. A, C, E are collinear and $\measuredangle CEO_1 = \measuredangle O_1CE \Rightarrow \measuredangle(AC, EO_1) = \measuredangle O_1CA$.

Step 103. $\measuredangle(AC, EO_1) = \measuredangle O_1CA$, $\measuredangle ACQ = \measuredangle EPQ$, $\measuredangle O_1CQ = \measuredangle CQO_1$, $\measuredangle CPO_1 = \measuredangle O_1CP$, $\measuredangle O_1PQ = \measuredangle PQO_1$ and AC is perpendicular to $EI \Rightarrow$ by angle chasing: $\measuredangle CPE = \measuredangle IEO_1$.

Step 104. $EI = IP \Rightarrow \measuredangle IEP = \measuredangle EPI$.

Step 105. AC is perpendicular to EI, AC is perpendicular to IN and EP is perpendicular to $IO_1 \Rightarrow \measuredangle(AC, EN) = \measuredangle(IO_1, EP)$.

Step 106. $\measuredangle IEP = \measuredangle EPI$, AC is perpendicular to EI and AC is perpendicular to $IN \Rightarrow \measuredangle EPI = \measuredangle NEP$.

Step 107. $\measuredangle(AC, EN) = \measuredangle(IO_1, EP)$ and $\measuredangle EPI = \measuredangle NEP \Rightarrow \measuredangle(AC, EP) = \measuredangle O_1IP$.

Step 108. $\measuredangle IEO_1 = \measuredangle O_1PI$ and $\measuredangle CPE = \measuredangle IEO_1 \Rightarrow \measuredangle CPE = \measuredangle O_1PI$.

Step 109. A, C, E are collinear and $\measuredangle(AC, EP) = \measuredangle O_1IP \Rightarrow \measuredangle CEP = \measuredangle O_1IP$.

Step 110. $\measuredangle CPE = \measuredangle O_1PI$ and $\measuredangle CEP = \measuredangle O_1IP \Rightarrow \frac{CE}{CP} = \frac{O_1I}{O_1P}$.

Step 111. $\measuredangle ACQ = \measuredangle EPQ$, $\measuredangle O_1CQ = \measuredangle CQO_1$, $\measuredangle CPO_1 = \measuredangle O_1CP$, $\measuredangle O_1PQ = \measuredangle PQO_1$ and AC is perpendicular to $EI \Rightarrow$ by angle chasing: $\measuredangle CPO_1 = \measuredangle IEP$.

Step 112. $\measuredangle IEP = \measuredangle EPI$ and $\measuredangle CPO_1 = \measuredangle IEP \Rightarrow \measuredangle CPO_1 = \measuredangle EPI$.

Step 113. $\measuredangle CPO_1 = \measuredangle O_1CP$ and $\measuredangle CPO_1 = \measuredangle IEP \Rightarrow \measuredangle O_1CP = \measuredangle IEP$.

Step 114. $\measuredangle CPO_1 = \measuredangle EPI$ and $\measuredangle O_1CP = \measuredangle IEP \Rightarrow \frac{CO_1}{CP} = \frac{EI}{EP}$.

Step 115. $DI = EI$, $DI = IR$ and $DI = IP \Rightarrow I$ is the circumcenter of ERP.

Step 116. I is the circumcenter of ERP and AE is perpendicular to $EI \Rightarrow \measuredangle AER = \measuredangle EPR$.

Step 117. A, C, E are collinear, A, R, P are collinear and $\measuredangle AER = \measuredangle EPR \Rightarrow \measuredangle(AC, ER) = \measuredangle(EP, AR)$.

Step 118. A, C, E are collinear, A, R, P are collinear and $\measuredangle(AC, ER) = \measuredangle(EP, AR) \Rightarrow \measuredangle AER = \measuredangle EPA$.

Step 119. A, R, P are collinear and $\measuredangle AER = \measuredangle EPR \Rightarrow \measuredangle AEP = \measuredangle ERA$.

Step 120. $\measuredangle AER = \measuredangle EPA$ and $\measuredangle AEP = \measuredangle ERA \Rightarrow \frac{AE}{AP} = \frac{AR}{AE}$ and $\frac{EA}{EP} = \frac{RA}{RE}$.

Step 121. $DI = EI$, $DI = IR$, $DI = IP$ and $EI = FI \Rightarrow I$ is the circumcenter of FRP.

Step 122. I is the circumcenter of FRP and AF is perpendicular to $FI \Rightarrow \measuredangle AFR = \measuredangle FPR$.

Step 123. A, R, P are collinear and $\measuredangle AFR = \measuredangle FPR \Rightarrow \measuredangle AFP = \measuredangle FRA$.

Step 124. A, R, P are collinear $\Rightarrow \measuredangle FAR = \measuredangle FAP$.

Step 125. $\measuredangle AFP = \measuredangle FRA$ and $\measuredangle FAR = \measuredangle FAP \Rightarrow \frac{FA}{FR} = \frac{PA}{PF}$.

Step 126. $DI = EI$, $DI = IR$, $EI = FI$ and $\frac{FB}{FD} = \frac{RI}{RE} \Rightarrow \frac{FB}{FD} = \frac{FI}{ER}$.

Step 127. $CD = CE$ and $\frac{DC}{DE} = \frac{FI}{FR} \Rightarrow \frac{EC}{ED} = \frac{FI}{FR}$.

Step 128. $AC = CG$ and $\frac{CA}{CN} = \frac{DF}{DE} \Rightarrow \frac{CN}{CG} = \frac{DE}{DF}$.

Step 129. $PO_2 = O_2Q$ and $\frac{BF}{BP} = \frac{O_2I}{O_2P} \Rightarrow \frac{BF}{BP} = \frac{O_2I}{O_2Q}$.

Step 130. $FI = IP$, $PO_2 = O_2Q$ and $\frac{PB}{PO_2} = \frac{PF}{PI} \Rightarrow \frac{BP}{O_2Q} = \frac{FP}{FI}$.

Step 131. $PO_1 = O_1Q$ and $\frac{CE}{CP} = \frac{O_1I}{O_1P} \Rightarrow \frac{CE}{CP} = \frac{O_1I}{O_1Q}$.

Step 132. $CO_1 = O_1Q$, $EI = FI$ and $\frac{CO_1}{CP} = \frac{EI}{EP} \Rightarrow \frac{CP}{O_1Q} = \frac{EP}{FI}$.

Step 133. $AE = AF$ and $\frac{AE}{AP} = \frac{AR}{AE} \Rightarrow \frac{AF}{AP} = \frac{AR}{AF}$.

Step 134. $AE = AF$ and $\frac{EA}{EP} = \frac{RA}{RE} \Rightarrow \frac{AF}{EP} = \frac{RA}{RE}$.

Step 135. $\frac{AF}{AP} = \frac{AR}{AF}$, $\frac{AF}{EP} = \frac{RA}{RE}$, $\frac{BF}{BP} = \frac{O_2I}{O_2Q}$, $\frac{BP}{O_2Q} = \frac{FP}{FI}$, $\frac{CE}{CP} = \frac{O_1I}{O_1Q}$, $\frac{CP}{O_1Q} = \frac{EP}{FI}$, $\frac{CN}{CG} = \frac{DE}{DF}$, $\frac{EC}{ED} = \frac{FI}{FR}$, $\frac{FA}{FR} = \frac{PA}{PF}$ and $\frac{FB}{FD} = \frac{FI}{ER} \Rightarrow$ by ratio chasing: $\frac{CN}{CG} = \frac{IO_1}{IO_2}$.

Step 136. $DI = EI$, $DI = IP$ and $EI = FI \Rightarrow I$ is the circumcenter of EFP.

Step 137. I is the circumcenter of EFP and AF is perpendicular to $FI \Rightarrow \measuredangle AFP = \measuredangle FEP$.

Step 138. EP is perpendicular to IO_1 and FP is perpendicular to $IO_2 \Rightarrow \measuredangle(EP, IO_1) = \measuredangle(FP, IO_2)$.

Step 139. $\measuredangle AFP = \measuredangle FEP$, AI is perpendicular to CN and AI is perpendicular to $EF \Rightarrow \measuredangle AFP = \measuredangle(CN, EP)$.

Step 140. $\measuredangle(EP, IO_1) = \measuredangle(FP, IO_2)$ and $\measuredangle AFP = \measuredangle(CN, EP) \Rightarrow \measuredangle(AF, IO_2) = \measuredangle(CN, IO_1)$.

Step 141. A, B, F are collinear, $\measuredangle(AF, IO_2) = \measuredangle(CN, IO_1)$ and AB is parallel to $CG \Rightarrow \measuredangle NCG = \measuredangle O_1IO_2$.

Step 142. $\measuredangle NCG = \measuredangle O_1IO_2$ and $\frac{CN}{CG} = \frac{IO_1}{IO_2} \Rightarrow \measuredangle CNG = \measuredangle IO_1O_2$.

Step 143. I is the circumcenter of DER and AE is perpendicular to $EI \Rightarrow \measuredangle AED = \measuredangle ERD$.

Step 144. A, C, E are collinear, $\measuredangle AED = \measuredangle ERD$, AI is perpendicular to EF and DR is perpendicular to $EF \Rightarrow \measuredangle(AC, DE) = \measuredangle(ER, AI)$.

Step 145. AI is perpendicular to CN and CI is perpendicular to $DE \Rightarrow \measuredangle(AI, CN) = \measuredangle(DE, CI)$.

Step 146. $\measuredangle(AC, DE) = \measuredangle(ER, AI)$ and $\measuredangle(AI, CN) = \measuredangle(DE, CI) \Rightarrow \measuredangle ACI = \measuredangle(ER, CN)$.

Step 147. A, C, E are collinear, $\measuredangle ACI = \measuredangle(ER, CN)$, $\measuredangle ANE = \measuredangle ICE$, AC is perpendicular to EI, AC is perpendicular to IN, AI is perpendicular to CN and AI is perpendicular to $EF \Rightarrow \measuredangle ANI = \measuredangle FER$.

Step 148. $\measuredangle AFE = \measuredangle AIE$, $\measuredangle AFR = \measuredangle FER$, AC is perpendicular to EI and AC is perpendicular to $IN \Rightarrow \measuredangle AIN = \measuredangle FRE$.

Step 149. $\measuredangle AIN = \measuredangle FRE$ and $\measuredangle ANI = \measuredangle FER \Rightarrow \frac{AN}{AI} = \frac{FE}{FR}$.

Step 150. A, C, E are collinear, AG is parallel to CN, AI is perpendicular to CN and AI is perpendicular to $EF \Rightarrow \measuredangle CAG = \measuredangle AEF$.

Step 151. A, B, F are collinear, A, C, E are collinear and AB is parallel to $CG \Rightarrow \measuredangle ACG = \measuredangle EAF$.

Step 152. $\measuredangle CAG = \measuredangle AEF$ and $\measuredangle ACG = \measuredangle EAF \Rightarrow \frac{FA}{FE} = \frac{GC}{GA}$.

Step 153. $EI = FI$ and $\frac{EA}{EI} = \frac{FA}{FI} \Rightarrow \frac{AE}{FI} = \frac{FA}{FI}$.

Step 154. $AE = AF$, $EI = FI$ and $\frac{EA}{EI} = \frac{EN}{EC} \Rightarrow \frac{FA}{FI} = \frac{EN}{EC}$.

Step 155. $AE = AF$ and $\frac{AI}{AE} = \frac{NC}{NE} \Rightarrow \frac{AI}{AF} = \frac{NC}{NE}$.

Step 156. $\frac{AE}{FI} = \frac{FA}{FI}$, $\frac{AI}{AF} = \frac{NC}{NE}$, $\frac{AN}{AI} = \frac{FE}{FR}$, $\frac{CN}{CG} = \frac{DE}{DF}$, $\frac{EC}{ED} = \frac{FI}{FR}$, $\frac{FA}{FE} = \frac{GC}{GA}$ and $\frac{FA}{FI} = \frac{EN}{EC} \Rightarrow$ by ratio chasing: $\frac{AE}{DF} = \frac{AN}{AG}$.

Step 157. A, C, E are collinear, $\measuredangle NAE = \measuredangle CIE$ and AC is perpendicular to $EI \Rightarrow AN$ is perpendicular to CI.

Step 158. AN is perpendicular to CI and CI is perpendicular to $DE \Rightarrow AN$ is parallel to DE.

Step 159. $AE = AF$ and $\frac{AE}{DF} = \frac{AN}{AG} \Rightarrow \frac{FA}{FD} = \frac{AN}{AG}$.

Step 160. $\measuredangle AFD = \measuredangle FED$, AN is parallel to DE, AG is parallel to CN, AI is perpendicular to CN and AI is perpendicular to $EF \Rightarrow \measuredangle AFD = \measuredangle GAN$.

Step 161. $\measuredangle AFD = \measuredangle GAN$ and $\frac{FA}{FD} = \frac{AN}{AG} \Rightarrow \measuredangle ADF = \measuredangle AGN$.

Step 162. $DI = EI$, $DI = IP$ and $EI = FI \Rightarrow I$ is the circumcenter of DFP.

Step 163. I is the circumcenter of DFP and AF is perpendicular to $FI \Rightarrow \measuredangle AFD = \measuredangle FPD$.

Step 164. $DI = EI$ and $DI = IP \Rightarrow I$ is the circumcenter of DEP.

Step 165. I is the circumcenter of DEP and AE is perpendicular to $EI \Rightarrow \measuredangle AED = \measuredangle EPD$.

Step 166. A, B, F are collinear, P, Q, T are collinear, $\measuredangle FBQ = \measuredangle FPQ$ and $\measuredangle AFD = \measuredangle FPD \Rightarrow \measuredangle BQT = \measuredangle FDP$.

Step 167. A, C, E are collinear, P, Q, T are collinear, $\measuredangle ECQ = \measuredangle EPQ$ and $\measuredangle AED = \measuredangle EPD \Rightarrow \measuredangle CQT = \measuredangle EDP$.

Step 168. $\measuredangle BQT = \measuredangle FDP$ and $\measuredangle CQT = \measuredangle EDP \Rightarrow \measuredangle BQC = \measuredangle FDE$.

Step 169. $DI = EI$ and $EI = FI \Rightarrow DI = FI$.

Step 170. $DI = FI \Rightarrow \measuredangle DFI = \measuredangle IDF$.

Step 171. A, B, F are collinear, B, C, D are collinear, AB is perpendicular to FI and BC is perpendicular to $DI \Rightarrow \measuredangle BDI = \measuredangle BFI$.

Step 172. $\measuredangle BDI = \measuredangle BFI \Rightarrow B, D, F, I$ are cyclic.

Step 173. B, D, F, I are cyclic $\Rightarrow \measuredangle DBI = \measuredangle DFI$.

Step 174. A, C, E are collinear, B, C, D are collinear, AC is perpendicular to EI and BC is perpendicular to $DI \Rightarrow \measuredangle CDI = \measuredangle CEI$.

Step 175. $\measuredangle CDI = \measuredangle CEI \Rightarrow C, D, E, I$ are cyclic.

Step 176. C, D, E, I are cyclic $\Rightarrow \measuredangle ECI = \measuredangle EDI$.

Step 177. B, C, D are collinear, D, I, T are collinear, $\measuredangle DBI = \measuredangle DFI$ and $\measuredangle DFI = \measuredangle IDF \Rightarrow \measuredangle CBI = \measuredangle(IT, DF)$.

Step 178. A, C, E are collinear, D, I, T are collinear, CI is the bisector of $\measuredangle ACB$ and $\measuredangle ECI = \measuredangle EDI \Rightarrow \measuredangle BCI = \measuredangle(IT, DE)$.

Step 179. $\measuredangle CBI = \measuredangle(IT, DF)$ and $\measuredangle BCI = \measuredangle(IT, DE) \Rightarrow \measuredangle BIC = \measuredangle FDE$.

Step 180. $\measuredangle BIC = \measuredangle FDE$ and $\measuredangle BQC = \measuredangle FDE \Rightarrow \measuredangle BIC = \measuredangle BQC$.

Step 181. $\measuredangle BIC = \measuredangle BQC \Rightarrow B, C, I, Q$ are cyclic.

Step 182. B, C, I, Q are cyclic $\Rightarrow \measuredangle CBI = \measuredangle CQI$ and $\measuredangle BCQ = \measuredangle BIQ$.

Step 183. B, C, D are collinear, $\measuredangle(BC, DR) = \measuredangle(IT, EF)$, $\measuredangle CDF = \measuredangle DRF$, AI is perpendicular to CN and AI is perpendicular to $EF \Rightarrow \measuredangle(CN, IT) = \measuredangle RFD$.

Step 184. A, C, E are collinear, $\measuredangle AEF = \measuredangle ERF$, AI is perpendicular to CN and AI is perpendicular to $EF \Rightarrow \measuredangle ACN = \measuredangle ERF$.

Step 185. $\measuredangle ACN = \measuredangle ERF$ and $\measuredangle(CN, IT) = \measuredangle RFD \Rightarrow \measuredangle(AC, IT) = \measuredangle(ER, DF)$.

Step 186. B, C, D are collinear and $\measuredangle DBI = \measuredangle DFI \Rightarrow \measuredangle(BC, DF) = \measuredangle BIF$.

Step 187. D, I, T are collinear and $\measuredangle DFI = \measuredangle IDF \Rightarrow \measuredangle(DF, IT) = \measuredangle IFD$.

Step 188. $\measuredangle(BC, DF) = \measuredangle BIF$ and $\measuredangle(DF, IT) = \measuredangle IFD \Rightarrow \measuredangle(BC, IT) = \measuredangle(BI, DF)$.

Step 189. $\measuredangle(AC, IT) = \measuredangle(ER, DF)$ and $\measuredangle(BC, IT) = \measuredangle(BI, DF) \Rightarrow \measuredangle(AC, ER) = \measuredangle CBI$.

Step 190. $PO_1 = O_1Q$ and $PO_2 = O_2Q \Rightarrow PQ$ is perpendicular to O_1O_2.

Step 191. P, Q, T are collinear, EP is perpendicular to IO_1 and PQ is perpendicular to $O_1O_2 \Rightarrow \measuredangle(EP, IO_1) = \measuredangle(QT, O_1O_2)$.

Step 192. A, C, E are collinear, P, Q, T are collinear and $\measuredangle ECQ = \measuredangle EPQ$ $\Rightarrow \measuredangle(AC, EP) = \measuredangle CQT$.

Step 193. $\measuredangle(AC, EP) = \measuredangle CQT$ and $\measuredangle(EP, IO_1) = \measuredangle(QT, O_1O_2) \Rightarrow \measuredangle(AC, IO_1) = \measuredangle(CQ, O_1O_2)$.

Step 194. $\measuredangle(AC, ER) = \measuredangle CBI$ and $\measuredangle CBI = \measuredangle CQI \Rightarrow \measuredangle(AC, ER) = \measuredangle CQI$.

Step 195. $\measuredangle(AC, ER) = \measuredangle CQI$ and $\measuredangle(AC, IO_1) = \measuredangle(CQ, O_1O_2) \Rightarrow \measuredangle(ER, IQ) = \measuredangle IO_1O_2$.

Step 196. $\measuredangle(BC, IT) = \measuredangle(BI, DF)$ and $\measuredangle BCQ = \measuredangle BIQ \Rightarrow \measuredangle(CQ, IT) = \measuredangle(IQ, DF)$.

Step 197. $\measuredangle ADF = \measuredangle AGN$, $\measuredangle(ER, IQ) = \measuredangle IO_1O_2$, $\measuredangle CNG = \measuredangle IO_1O_2$ and AG is parallel to $CN \Rightarrow \measuredangle ADF = \measuredangle(ER, IQ)$.

Step 198. $\measuredangle(CQ, IT) = \measuredangle(IQ, DF) \Rightarrow \measuredangle CQI = \measuredangle(IT, DF)$.

Step 199. $\measuredangle ADF = \measuredangle(ER, IQ)$ and $\measuredangle CQI = \measuredangle(IT, DF) \Rightarrow \measuredangle(AD, IT) = \measuredangle(ER, CQ)$.

Step 200. P, Q, T are collinear and $\measuredangle ACQ = \measuredangle EPQ \Rightarrow \measuredangle ACQ = \measuredangle(EP, QT)$.

Step 201. $\measuredangle(AC, ER) = \measuredangle(EP, AR)$ and $\measuredangle ACQ = \measuredangle(EP, QT) \Rightarrow \measuredangle(AR, QT) = \measuredangle(ER, CQ)$.

Step 202. A, R, P are collinear, D, I, T are collinear, P, Q, T are collinear, $\measuredangle(AD, IT) = \measuredangle(ER, CQ)$ and $\measuredangle(AR, QT) = \measuredangle(ER, CQ) \Rightarrow \measuredangle ADT = \measuredangle APT$.

Step 203. $\measuredangle ADT = \measuredangle APT \Rightarrow A, D, P, T$ are cyclic.

Step 204. A, D, P, T are cyclic $\Rightarrow \measuredangle ADP = \measuredangle ATP$.

Step 205. A, B, F are collinear, P, Q, T are collinear, $\measuredangle FBQ = \measuredangle FPQ$, $\measuredangle AFP = \measuredangle FEP$, AI is perpendicular to CN and AI is perpendicular to EF $\Rightarrow \measuredangle BQT = \measuredangle(CN, EP)$.

Step 206. $\measuredangle(EP, IO_1) = \measuredangle(QT, O_1O_2)$ and $\measuredangle BQT = \measuredangle(CN, EP) \Rightarrow \measuredangle(BQ, CN) = \measuredangle O_2O_1I$.

Step 207. $\measuredangle(BQ, CN) = \measuredangle O_2O_1I$, $\measuredangle ADF = \measuredangle AGN$, $\measuredangle CNG = \measuredangle IO_1O_2$ and AG is parallel to $CN \Rightarrow \measuredangle ADF = \measuredangle(CN, BQ)$.

Step 208. $\measuredangle ADF = \measuredangle(CN, BQ)$ and $\measuredangle BQT = \measuredangle FDP \Rightarrow \measuredangle ADP = \measuredangle(CN, QT)$.

Step 209. P, Q, T are collinear, $\measuredangle ADP = \measuredangle(CN, QT)$ and $\measuredangle ADP = \measuredangle ATP$ $\Rightarrow \measuredangle ATQ = \measuredangle(CN, QT)$.

Step 210. $\measuredangle ATQ = \measuredangle(CN, QT) \Rightarrow AT$ is parallel to CN.

Step 211. AT is parallel to CN and AI is perpendicular to $CN \Rightarrow AI$ is perpendicular to AT

■

2.28 IMO 2020 P1

Original:

Consider the convex quadrilateral ABCD. The point P is in the interior of ABCD. The following ratio equalities hold: $\measuredangle PAD : \measuredangle PBA : \measuredangle DPA = 1 : 2 : 3 = \measuredangle CBP : \measuredangle BAP : \measuredangle BPC$. Prove that the following three lines meet in a point: the internal bisectors of angles $\measuredangle ADP$ and $\measuredangle PCB$ and the perpendicular bisector of segment AB.

Translated:

Let PAB be a triangle. Define point X such that BX is the bisector of $\measuredangle PBA$. Define point Y such that AY is the bisector of $\measuredangle PAB$. Define point Z such that $\measuredangle(PA, BX) = \measuredangle ZAB$ and $\measuredangle PAZ = \measuredangle XBA$. Define point T such that $\measuredangle PAZ = \measuredangle TPA$. Define point D on line AZ such that $\measuredangle PBA = \measuredangle DPT$. Define point U such that $\measuredangle PBU = \measuredangle YAB$. Define point V such that $\measuredangle PBU = \measuredangle VPB$. Define point C on line BU such that $\measuredangle PAB = \measuredangle CPV$. Define point O such that DO is the bisector of $\measuredangle PDA$ and CO is the bisector of $\measuredangle PCB$. Prove that $AO = BO$

Proof:

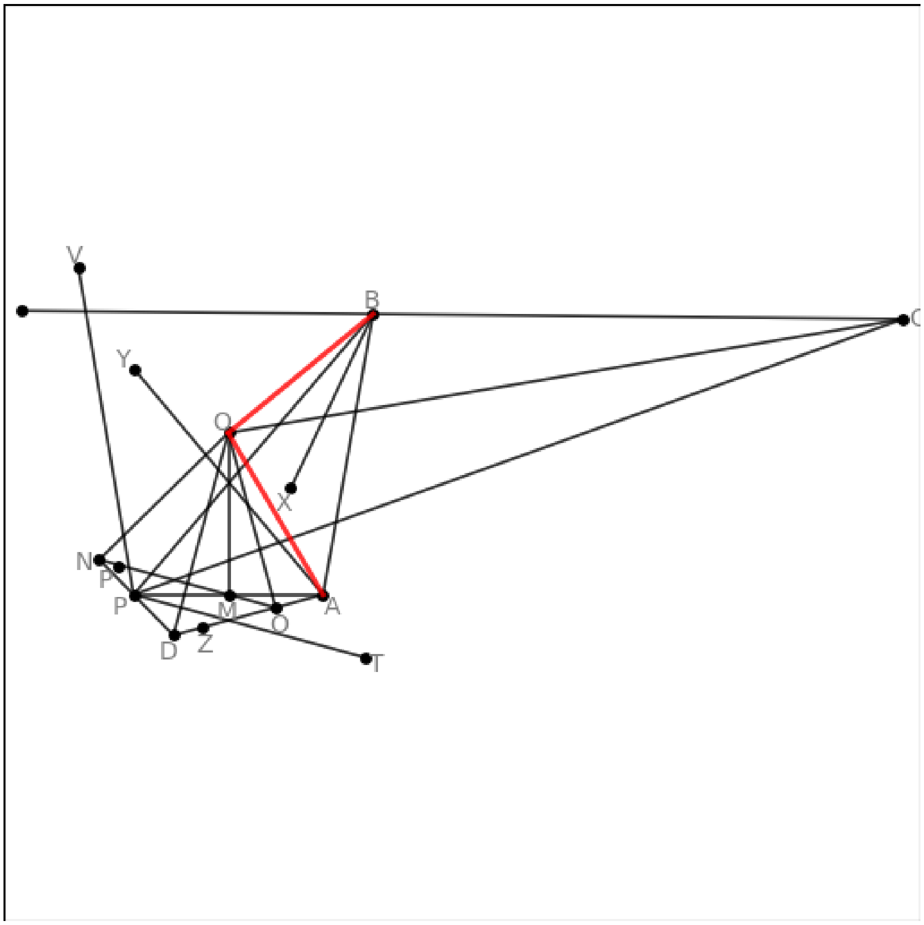

Figure 28: imo 2020 p1

Construct point M as the foot of O on line PA.

Construct point N as the foot of O on line PD.

Construct point E as the foot of O on line AD.

Step 1. P, D, N are collinear, A, D, E are collinear, PD is perpendicular to ON and AD is perpendicular to $OE \Rightarrow \measuredangle DNO = \measuredangle DEO$.

Step 2. $\measuredangle DNO = \measuredangle DEO \Rightarrow D, O, N, E$ are cyclic.

Step 3. D, O, N, E are cyclic $\Rightarrow \measuredangle ODE = \measuredangle ONE$.

Step 4. P, A, M are collinear, P, D, N are collinear, PA is perpendicular to OM and PD is perpendicular to $ON \Rightarrow \measuredangle PMO = \measuredangle PNO$.

Step 5. $\measuredangle PMO = \measuredangle PNO \Rightarrow P, O, M, N$ are cyclic.

Step 6. P, O, M, N are cyclic $\Rightarrow \measuredangle OPM = \measuredangle ONM$.

Step 7. P, A, M are collinear and $\measuredangle(PA, BX) = \measuredangle ZAB \Rightarrow \measuredangle ZAB = \measuredangle(AM, BX)$.

Step 8. $\measuredangle ZAB = \measuredangle(AM, BX)$ and BX is the bisector of $\measuredangle PBA \Rightarrow \measuredangle(PB, AM) = \measuredangle(BX, AZ)$.

Step 9. P, A, M are collinear, A, Z, D are collinear, A, D, E are collinear, $\measuredangle OPM = \measuredangle ONM$ and $\measuredangle ODE = \measuredangle ONE \Rightarrow \measuredangle(PO, AM) = \measuredangle(DO, AZ)$.

Step 10. $\measuredangle(PB, AM) = \measuredangle(BX, AZ)$ and $\measuredangle(PO, AM) = \measuredangle(DO, AZ) \Rightarrow \measuredangle BPO = \measuredangle(BX, DO)$.

Step 11. P, A, M are collinear, AY is the bisector of $\measuredangle PAB$, $\measuredangle PBU = \measuredangle VPB$ and $\measuredangle PBU = \measuredangle YAB \Rightarrow \measuredangle BPV = \measuredangle YAM$.

Step 12. B, U, C are collinear and $\measuredangle PBU = \measuredangle YAB \Rightarrow \measuredangle(PB, UC) = \measuredangle YAB$.

Step 13. $\measuredangle BPV = \measuredangle YAM$ and $\measuredangle(PB, UC) = \measuredangle YAB \Rightarrow \measuredangle(PV, UC) = \measuredangle MAB$.

Step 14. P, A, M are collinear and $\measuredangle PAZ = \measuredangle TPA \Rightarrow \measuredangle(PT, AM) = \measuredangle MAZ$.

Step 15. $\measuredangle(PT, AM) = \measuredangle MAZ$ and $\measuredangle ZAB = \measuredangle(AM, BX) \Rightarrow \measuredangle(PT, BX) = \measuredangle MAB$.

Step 16. B, U, C are collinear and CO is the bisector of $\measuredangle PCB \Rightarrow \measuredangle PCO = \measuredangle(CO, BU)$.

Step 17. $\measuredangle PAB = \measuredangle CPV$, AY is the bisector of $\measuredangle PAB$, $\measuredangle PBU = \measuredangle VPB$, $\measuredangle PBU = \measuredangle YAB$ and $\measuredangle PCO = \measuredangle(CO, BU) \Rightarrow$ by angle chasing: PV is perpendicular to CO.

Step 18. A, Z, D are collinear and DO is the bisector of $\measuredangle PDA \Rightarrow \measuredangle PDO = \measuredangle(DO, AZ)$.

Step 19. $\measuredangle PAZ = \measuredangle TPA$, $\measuredangle PAZ = \measuredangle XBA$, $\measuredangle PBA = \measuredangle DPT$, BX is the bisector of $\measuredangle PBA$ and $\measuredangle PDO = \measuredangle(DO, AZ) \Rightarrow$ by angle chasing: $\measuredangle(DO, PT) = \frac{1\pi}{2}$.

Step 20. $\measuredangle(PT, BX) = \measuredangle MAB$ and $\measuredangle(PV, UC) = \measuredangle MAB \Rightarrow \measuredangle(PT, BX) = \measuredangle(PV, UC)$.

Step 21. $\measuredangle(DO, PT) = \frac{1\pi}{2}$ and PV is perpendicular to $CO \Rightarrow \measuredangle(PT, DO) = \measuredangle(PV, CO)$.

Step 22. $\measuredangle(PT, BX) = \measuredangle(PV, UC)$ and $\measuredangle(PT, DO) = \measuredangle(PV, CO) \Rightarrow \measuredangle(BX, DO) = \measuredangle UCO$.

Step 23. B, U, C are collinear, $\measuredangle BPO = \measuredangle(BX, DO)$ and $\measuredangle(BX, DO) = \measuredangle UCO \Rightarrow \measuredangle BPO = \measuredangle BCO$.

Step 24. $\measuredangle BPO = \measuredangle BCO \Rightarrow P, B, C, O$ are cyclic.

Step 25. P, B, C, O are cyclic and CO is the bisector of $\measuredangle PCB \Rightarrow PO = BO$.

Step 26. P, D, N are collinear, A, D, E are collinear, PD is perpendicular to ON and AD is perpendicular to $OE \Rightarrow \measuredangle DEO = \measuredangle OND$.

Step 27. P, D, N are collinear, A, Z, D are collinear, A, D, E are collinear and $\measuredangle PDO = \measuredangle(DO, AZ) \Rightarrow DO$ is the bisector of $\measuredangle EDN$.

Step 28. DO is the bisector of $\measuredangle EDN$ and $\measuredangle DEO = \measuredangle OND \Rightarrow ON = OE$.

Step 29. $ON = OE \Rightarrow \measuredangle ONE = \measuredangle NEO$.

Step 30. P, A, M are collinear, A, D, E are collinear, PA is perpendicular to OM and AD is perpendicular to $OE \Rightarrow \measuredangle AMO = \measuredangle AEO$.

Step 31. $\measuredangle AMO = \measuredangle AEO \Rightarrow A, O, M, E$ are cyclic.

Step 32. A, O, M, E are cyclic $\Rightarrow \measuredangle AOE = \measuredangle AME$.

Step 33. P, A, M are collinear, $\measuredangle OPM = \measuredangle ONM$, $\measuredangle AOE = \measuredangle AME$ and $\measuredangle ONE = \measuredangle NEO \Rightarrow \measuredangle PAO = \measuredangle OPA$.

Step 34. $\measuredangle PAO = \measuredangle OPA \Rightarrow PO = AO$.

Step 35. $PO = AO$ and $PO = BO \Rightarrow AO = BO$

■

2.29 IMO 2021 P3

Original:

Let D be an interior point of the triangle ABC with AB ¿ AC so that $\measuredangle DAB = \measuredangle CAD$. The point E on the segment AC satisfies $\measuredangle ADE = \measuredangle BCD$, the point F on the segment AB satisfies $\measuredangle FDA = \measuredangle DBC$, and the point X on the line AC satisfies CX = BX. Let O1 and O2 be the circumcenters of the triangles ADC and EXD, respectively. Prove that the lines BC, EF, and O1O2 are concurrent.

Translated:

Let ABC be a triangle. Define point D such that AD is the bisector of $\measuredangle BAC$. Define point E on line AC such that $\measuredangle ADE = \measuredangle BCD$. Define point F on line AB such that $\measuredangle ADF = \measuredangle CBD$. Define point X on line AC such that $BX = CX$ and $\measuredangle BCX = \measuredangle XBC$. Define point O_1 as the circumcenter of triangle CDA. Define point O_2 as the circumcenter of triangle EDX. Define point Y as the intersection of lines BC and EF. Prove that O_1, O_2, Y are collinear

Proof:

Not solved.

2.30 IMO 2022 P4

Original:

Let ABCDE be a convex pentagon such that BC = DE. Assume that there is a point T inside ABCDE with TB = TD, TC = TE and $\measuredangle ABT = \measuredangle TEA$. Let line AB intersect lines CD and CT at points P and Q, respectively. Assume that the points P, B, A, Q occur on their line in that order. Let line AE intersect CD and DT at points R and S, respectively. Assume that the points R, E, A, S occur on their line in that order. Prove that the points P, S, Q, R lie on a circle.

Translated:

Let BCD be a triangle. Define point E such that $BC = DE$. Define point T such that $BT = DT$ and $CT = ET$. Define point A such that $\measuredangle AET = \measuredangle TBA$. Define point P as the intersection of lines AB and CD. Define point Q as the intersection of lines AB and CT. Define point R as the intersection of lines AE and CD. Define point S as the intersection of lines AE and DT. Prove that P, Q, R, S are cyclic

Proof:

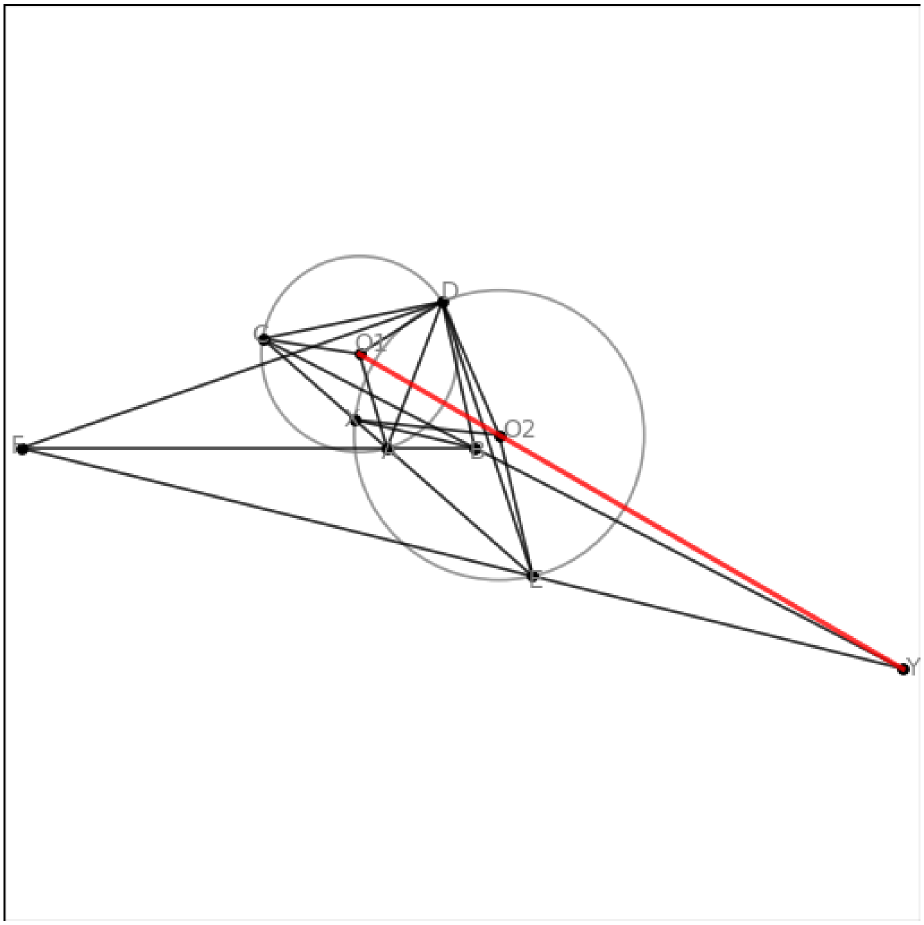

Figure 29: imo 2021 p3

Step 1. $BC = DE$, $BT = DT$ and $CT = ET \Rightarrow \measuredangle BTC = \measuredangle DTE$, $\measuredangle CBT = \measuredangle EDT$ and $\measuredangle BCT = \measuredangle DET$.

Step 2. C, Q, T are collinear, D, S, T are collinear and $\measuredangle BTC = \measuredangle DTE \Rightarrow \measuredangle BTQ = \measuredangle STE$.

Step 3. A, B, Q are collinear, A, E, S are collinear and $\measuredangle AET = \measuredangle TBA \Rightarrow \measuredangle QBT = \measuredangle TES$.

Step 4. $\measuredangle QBT = \measuredangle TES$ and $\measuredangle BTQ = \measuredangle STE \Rightarrow \frac{TB}{TE} = \frac{TQ}{TS}$.

Step 5. $BT = DT$, $CT = ET$ and $\frac{TB}{TE} = \frac{TQ}{TS} \Rightarrow \frac{TC}{TD} = \frac{TS}{TQ}$.

Step 6. C, Q, T are collinear and D, S, T are collinear $\Rightarrow \measuredangle CTD = \measuredangle QTS$.

Step 7. $\measuredangle CTD = \measuredangle QTS$ and $\frac{TC}{TD} = \frac{TS}{TQ} \Rightarrow \measuredangle DCT = \measuredangle TSQ$.

Step 8. C, Q, T are collinear, D, S, T are collinear and $\measuredangle DCT = \measuredangle TSQ \Rightarrow \measuredangle DCQ = \measuredangle DSQ$.

Step 9. D, S, T are collinear and $\measuredangle CBT = \measuredangle EDT \Rightarrow \measuredangle CBT = \measuredangle EDS$.

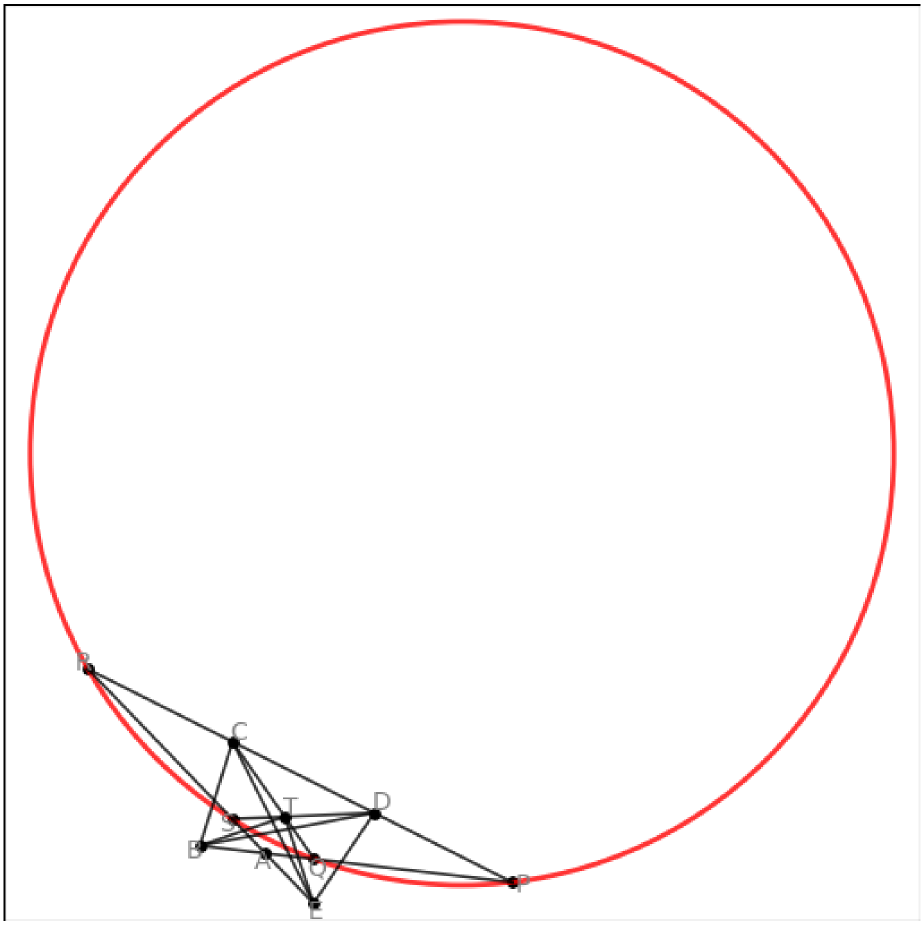

Figure 30: imo 2022 p4

Step 10. C, Q, T are collinear and $\measuredangle BCT = \measuredangle DET \Rightarrow \measuredangle BCQ = \measuredangle DET$.

Step 11. $\measuredangle CBT = \measuredangle EDS$, $\measuredangle BCQ = \measuredangle DET$, $\measuredangle DCQ = \measuredangle DSQ$ and $\measuredangle AET = \measuredangle TBA \Rightarrow$ by angle chasing: $\measuredangle(AB, QS) = \measuredangle(CD, AE)$.

Step 12. A, B, P are collinear, A, B, Q are collinear, A, E, R are collinear, A, E, S are collinear, C, D, P are collinear, C, D, R are collinear and $\measuredangle(AB, QS) = \measuredangle(CD, AE) \Rightarrow \measuredangle PQS = \measuredangle PRS$.

Step 13. $\measuredangle PQS = \measuredangle PRS \Rightarrow P, Q, R, S$ are cyclic

■
